# Supplementary material for: Boosting the performance of single-atom catalysts via external electric field polarization
Source: Nat Commun. 2022 Jun 2;13:3063. doi: 10.1038/s41467-022-30766-x (PMC9163078; doi:10.1038/s41467-022-30766-x)
Supplement: Supplementary file 1 — Supplementary Information [file 41467_2022_30766_MOESM1_ESM.pdf]

Supplementary Information

Boosting the performance of single-atom catalysts via external electric field polarization

Yanghang Pan[1#], Xinzhu Wang[1,2#], Weiyang Zhang[3], Lingyu Tang[1], Zhangyan Mu[1], Cheng Liu[1], Bailin Tian[1], Muchun Fei[1], Yamei Sun[1], Huanhuan Su[4], Libo Gao[4], Peng Wang[3,5], Xiangfeng Duan[6*], Jing Ma[1,2*] and Mengning Ding[1*]

[1] Key Laboratory of Mesoscopic Chemistry, School of Chemistry and Chemical Engineering, Nanjing University, Nanjing, Jiangsu, 210023, China

[2] Jiangsu Key Laboratory of Advanced Organic Materials, School of Chemistry and Chemical Engineering, Nanjing University, Nanjing, Jiangsu, 210023, China

[3] National Laboratory of Solid State Microstructures, Jiangsu Key Laboratory of Artificial Functional Materials, College of Engineering and Applied Sciences, Collaborative Innovation Center of Advanced Microstructures, Nanjing University, Nanjing, Jiangsu, 210093, China.

[4] National Laboratory of Solid State Microstructures, School of Physics, Collaborative Innovation Center of Advanced Microstructures, Nanjing University, Nanjing, Jiangsu, 210093, China.

[5] Department of Physics, University of Warwick, Coventry CV4 7AL, UK.

[6] Department of Chemistry and Biochemistry, University of California, Los Angeles, CA 90095, USA.

[*]Corresponding Authors: mding@nju.edu.cn; majing@nju.edu.cn; xduan@chem.ucla.edu

Characterizations of MoS_2 and Pt SAs-MoS_2

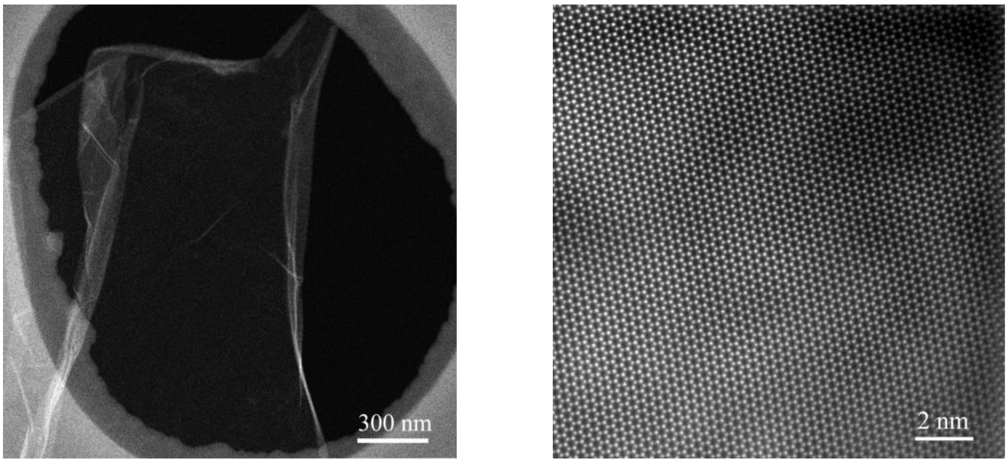

Supplementary Figure 1. HAADF-STEM images of mechanically exfoliated MoS_2.

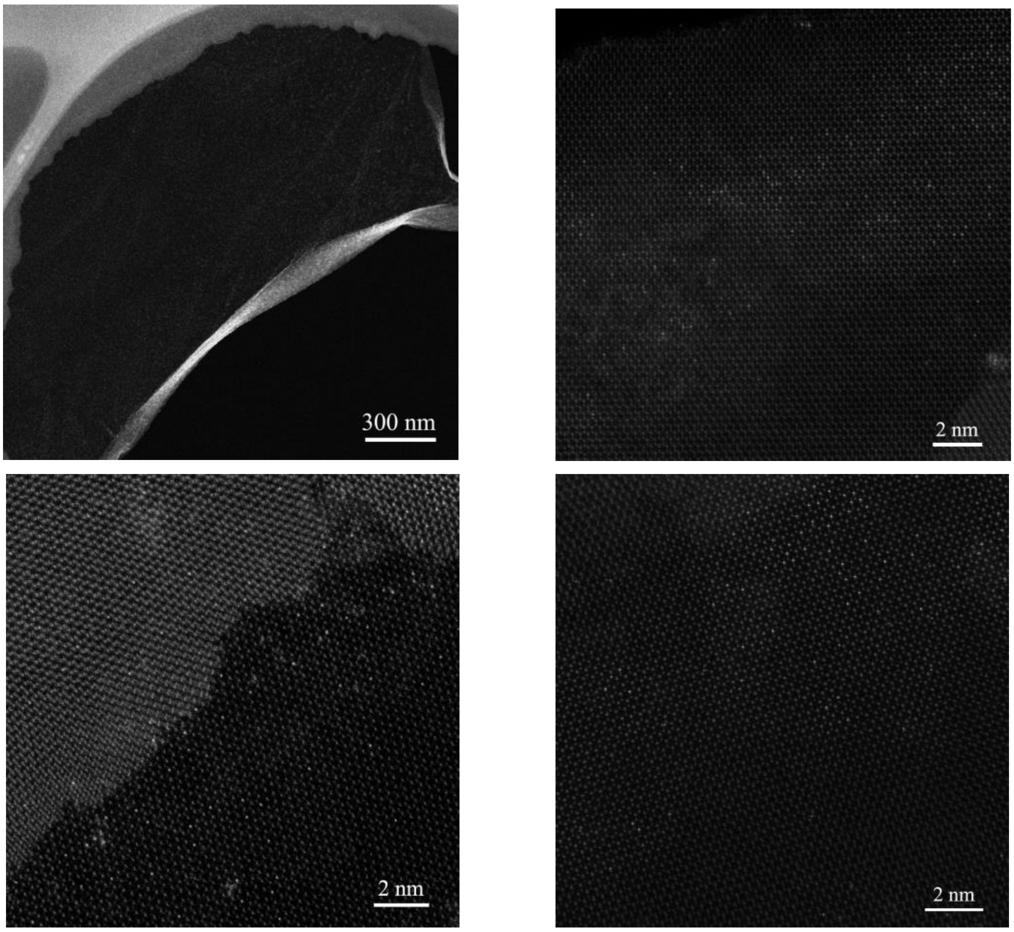

Supplementary Figure 2. HAADF-STEM images of Pt SAs on mechanically exfoliated MoS_2.

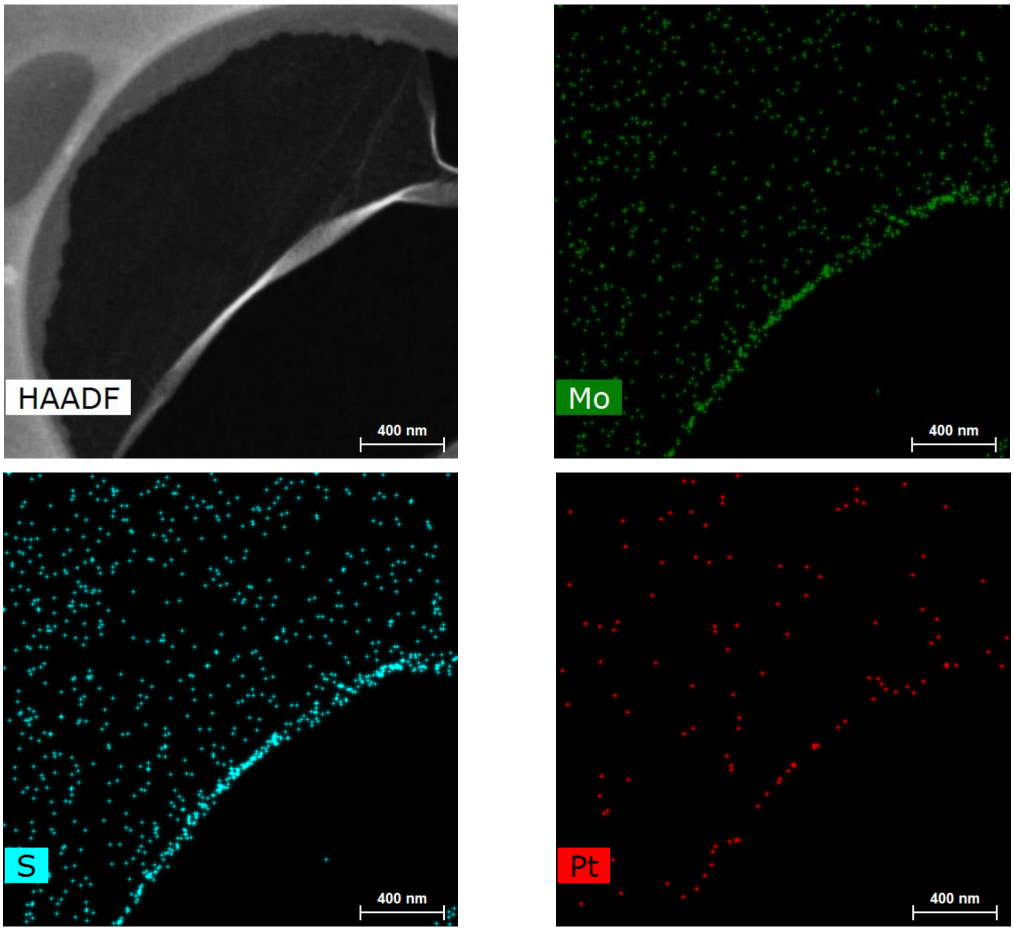

Supplementary Figure 3. STEM-EDS elemental mappings (Mo, S and Pt) of Pt SAs on mechanically exfoliated MoS_2.

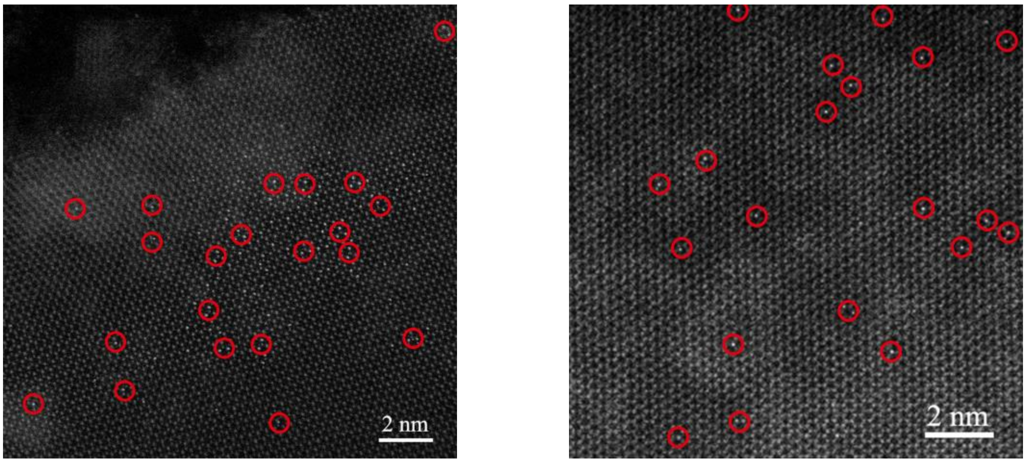

Supplementary Figure 4. HAADF-STEM images of Pt SAs on mechanically exfoliated MoS_2 after HER stability test.

To verify that the UV-assisted reduction process is necessary for synthesizing 2D SACs proposed in this work, we performed HER tests (**Supplementary Figure 5**) and STEM characterizations (**Supplementary Figure 6**) on Pt-MoS_2 samples without adding any reactants, without UV radiation and under UV radiation.

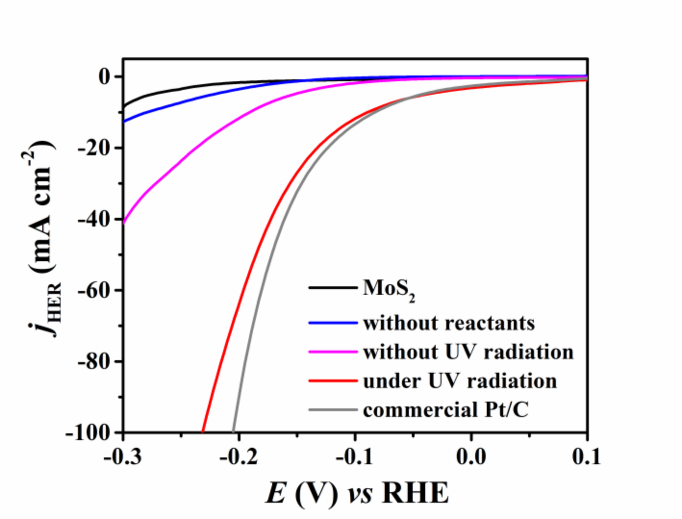

Supplementary Figure 5. HER polarization curves of MoS_2 (black), commercial Pt/C (gray), Pt-MoS_2 without reactants (blue), without UV radiation (magenta) and under UV radiation (red).

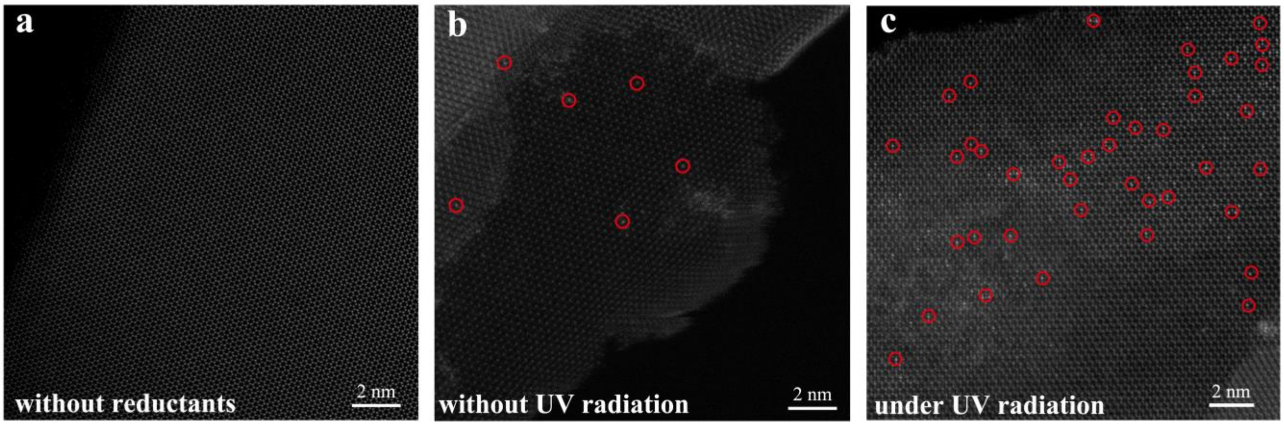

Supplementary Figure 6. HAADF-STEM images of Pt-MoS_2 without reactants (**a**), without UV radiation (**b**) and under UV radiation (**c**).

Supplementary Table 1. Area densities of Pt SAs on MoS_2 samples from HAADF-STEM images.

serial number	area (nm^2)	number of Pt SAs	area density (atom nm^{-2})
1	246.5	88	0.357
2	196.0	87	0.444
3	412.1	135	0.328
4	193.2	97	0.502
5	278.9	94	0.337
6	295.8	125	0.423
7	234.1	91	0.389
8	231.0	124	0.537
9	234.1	128	0.547
10	327.6	142	0.433
11	246.5	98	0.398
12	176.9	63	0.356
13	110.3	44	0.399
14	121.0	66	0.545
15	139.2	55	0.395
total	3443.2	1437	0.417
standard deviation of area density			0.072

Due to the fact that the thickness of MoS_2 nanosheets mainly used in this work is less than 5 nm (~7 layers), the mass loading of Pt SAs anchored on MoS_2 (monolayer and 7 layers) was approximately determined as:

$$\text{mass loading}_{\text{Pt}}\ (\text{monolayer}) = \frac{195.078 \times 0.417}{(195.078 \times 0.417 + 160.07 \times 10) \times 1} = 4.84\ \text{wt\%}$$

$$\text{mass loading}_{\text{Pt}}\ (\text{7 layers}) = \frac{195.078 \times 0.417}{(195.078 \times 0.417 + 160.07 \times 10) \times 7} = 0.69\ \text{wt\%}$$

In order to accurately explore the doping effect of Pt SAs introduced into 2H-MoS_2, we measured FET transfer characteristics (R.T. in vacuum) of the freshly prepared mechanically exfoliated MoS_2 (MoS_2-I), MoS_2 impregnated by the mixed solution (without K_2PtCl_6) under 365 nm UV-light radiation (MoS_2-II), and MoS_2 impregnated by the mixed solution (with K_2PtCl_6) under 365 nm UV-light radiation (Pt SAs-MoS_2). As is shown in **Supplementary Figure 7**, the threshold voltage of Pt SAs-MoS_2 was clearly shifted positively compared to MoS_2-I and MoS_2-II samples, demonstrating the p-doping effect from Pt SAs.

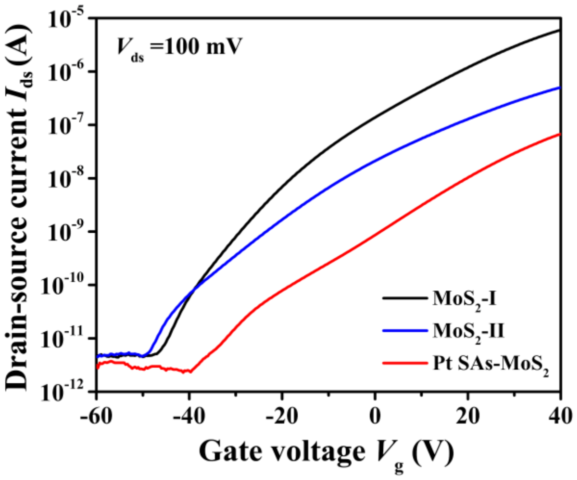

Supplementary Figure 7. Transfer characteristics of MoS_2-I (black), MoS_2-II (blue) and Pt SAs-MoS_2 (red).

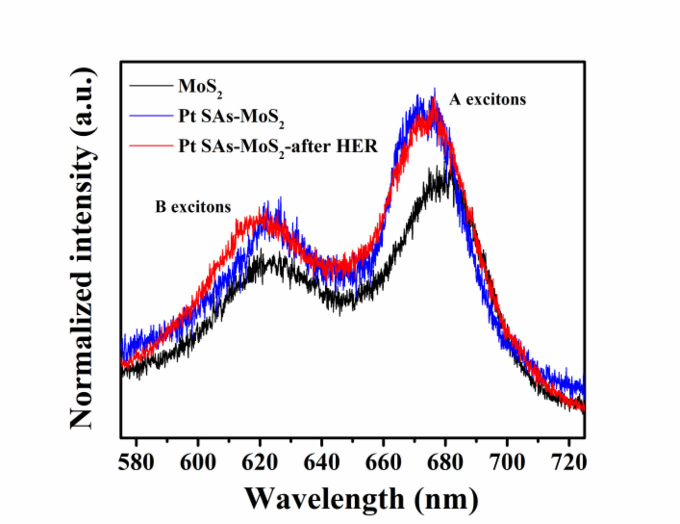

Supplementary Figure 8. PL spectroscopy of mechanically exfoliated MoS_2 (black) and Pt SAs-MoS_2 before (blue) and after HER stability test (red).

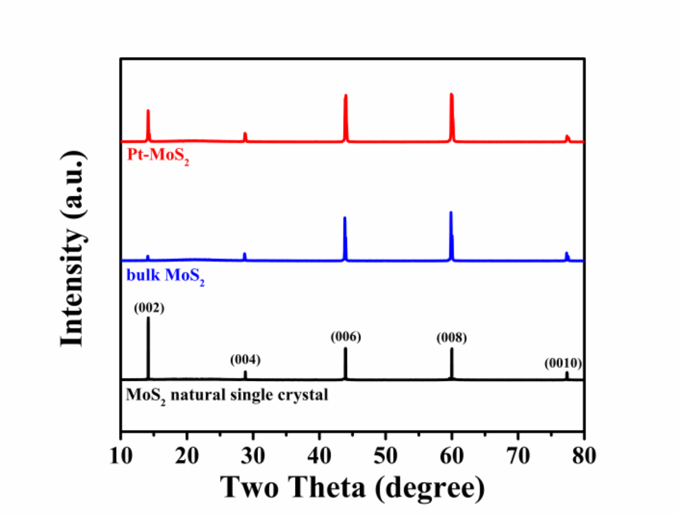

Supplementary Figure 9. XRD patterns of 2H-MoS_2 single crystal (black), bulk MoS_2 (blue) and Pt SAs-MoS_2 (red).

Supplementary Table 2. Atomic ratio (Mo:S) of bulk MoS_2 and Pt SAs-MoS_2 from XPS data.

Atomic concentration (%)	C 1s	S 2p	Mo 3d	Atomic ration (Mo:S)
MoS_2	26.42	48.89	24.69	1:1.98
Pt SAs-MoS_2	24.76	49.82	25.42	1:1.96

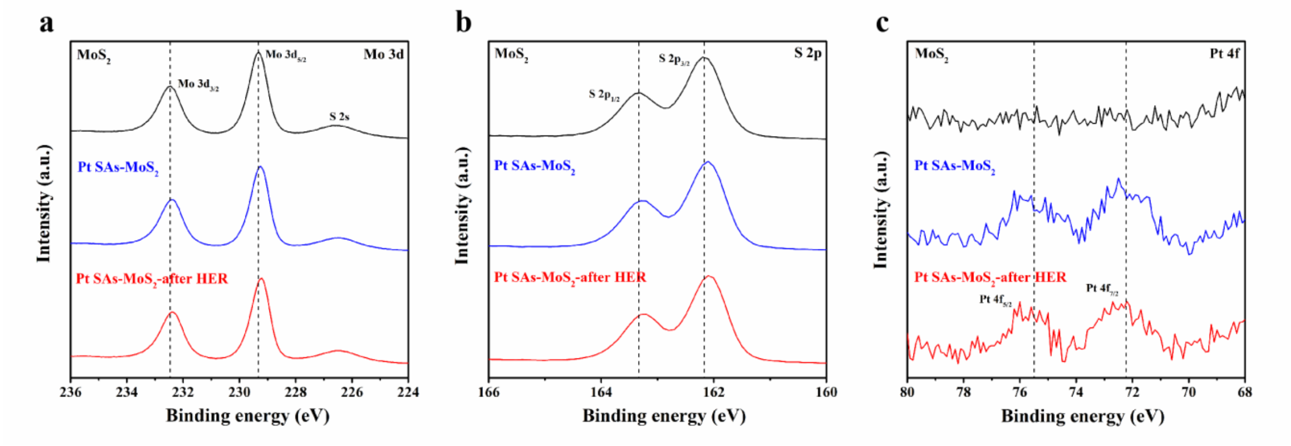

Supplementary Figure 10. XPS spectra showing Mo 3d (**a**), S 2p (**b**) and Pt 4f (**c**) core level peak regions for bulk MoS_2 (black) and Pt SAs-MoS_2 before (blue) and after HER stability test (red).

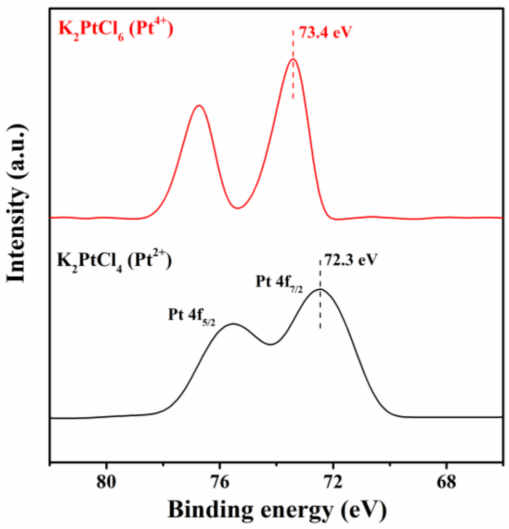

Supplementary Figure 11. XPS spectra showing Pt 4f core level peak regions for K_2PtCl_4 (Pt^{2+}) and K_2PtCl_6 (Pt^{4+}) samples.

Supplementary Table 3. The first shell fitting parameters of the EXAFS results for Pt foil and Pt SAs-MoS_2.

Sample	Scatter	CN	R/Å	σ^2/Å^2	ΔE_0/eV
Pt SAs-MoS_2	Pt-S	4.0	2.292	0.0073	4.964
Pt foil	Pt-Pt	12	2.765	0.0042	8.207
PtO_2	Pt-O	6.7	1.916	0.0028	10.872

R, distance between absorber and backscatter atoms. CN, coordination number. σ^2, Debye-Waller factor. ΔE_0, inner potential correction to account for the difference in the inner potential between the sample and the reference compound.

Characterizations of WSe_2 and Co SAs-WSe_2

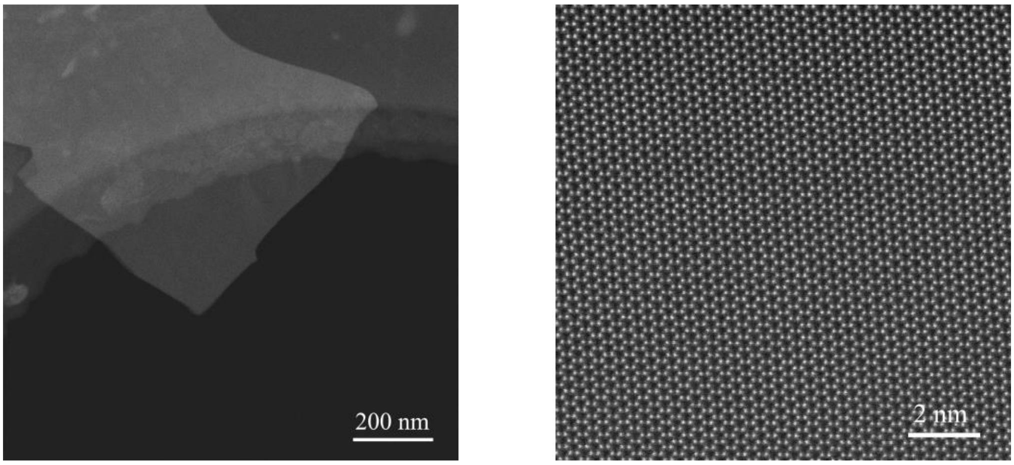

Supplementary Figure 12. HAADF-STEM images of mechanically exfoliated WSe_2.

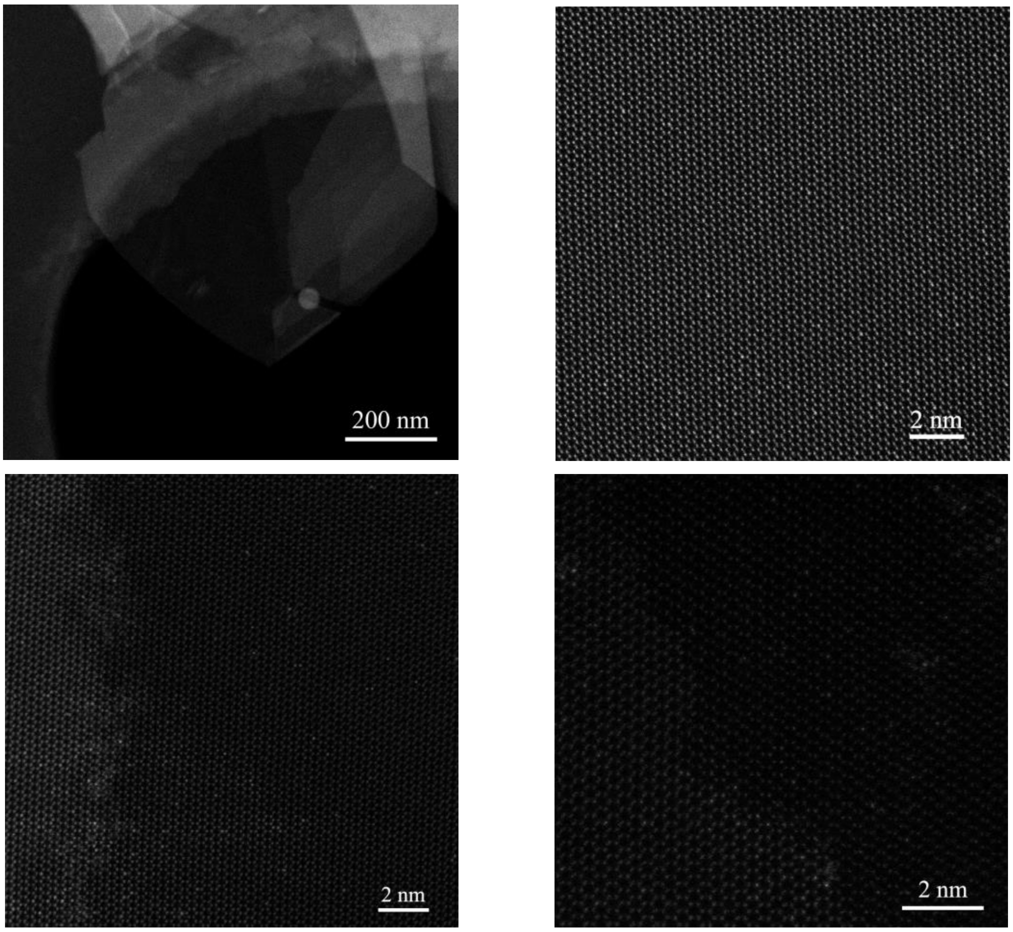

Supplementary Figure 13. HAADF-STEM images of Co SAs on mechanically exfoliated WSe_2.

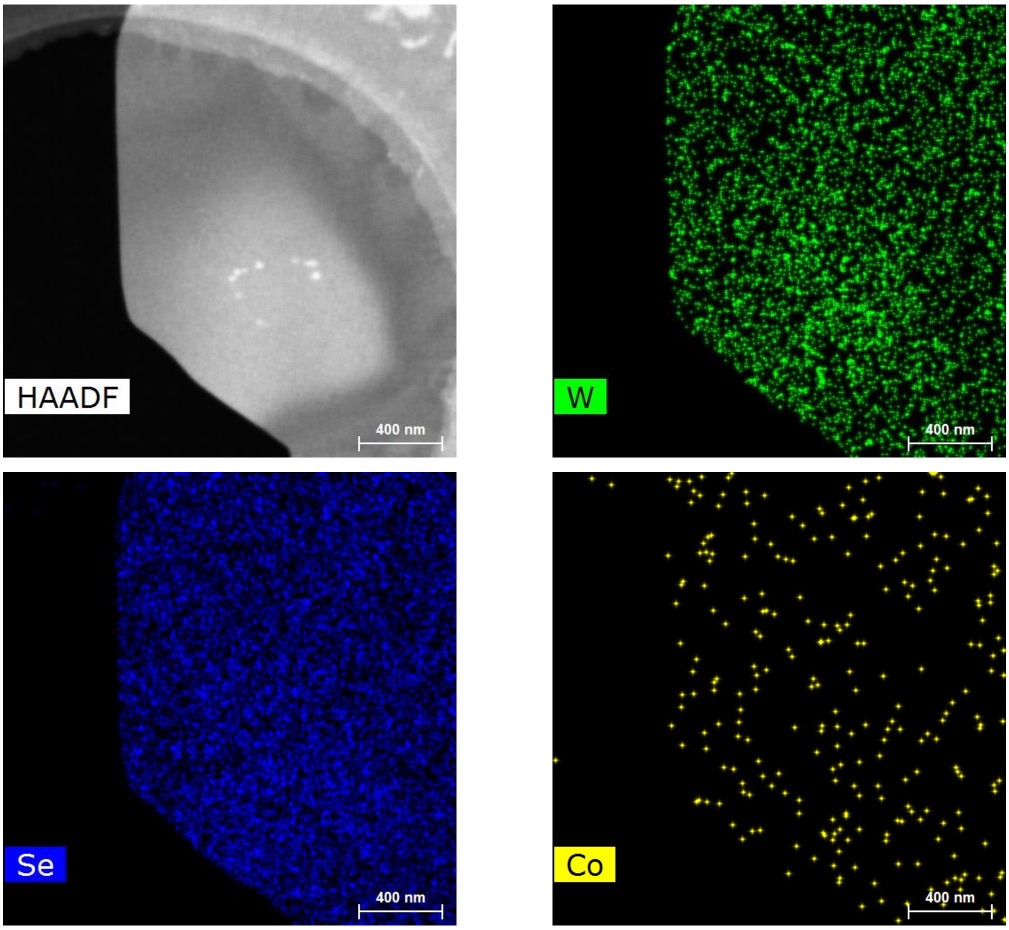

Supplementary Figure 14. STEM-EDS elemental mappings (W, Se and Co) of Co SAs-WSe_2 with mechanically exfoliated WSe_2.

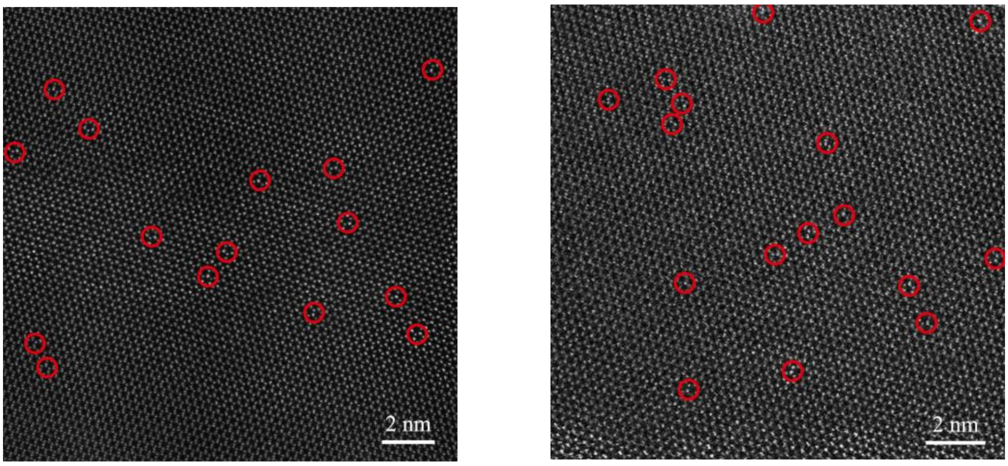

Supplementary Figure 15. HAADF-STEM images of Co SAs on mechanically exfoliated WSe_2 after OER stability test.

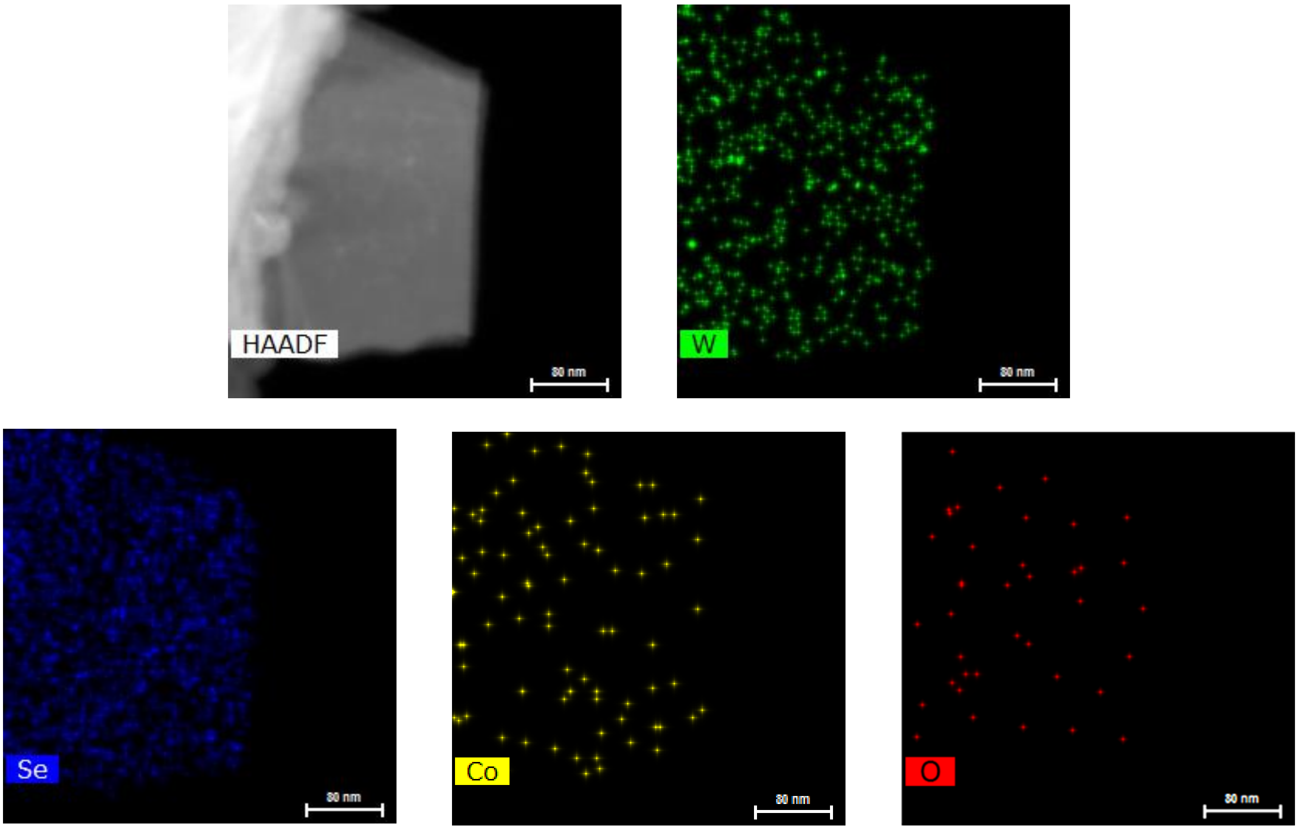

Supplementary Figure 16. STEM-EDS elemental mappings (W, Se, Co and O) of Co SAs on mechanically exfoliated WSe_2 after OER stability test.

Supplementary Table 4. Area densities of Co SAs on WSe_2 samples from HAADF-STEM images.

serial number	area (nm^2)	number of Co SAs	area density (atom nm^{-2})
1	660.5	237	0.359
2	269.2	147	0.546
3	267.6	131	0.490
4	670.8	282	0.420
5	166.9	74	0.443
6	212.5	69	0.325
7	216.1	85	0.393
8	201.6	78	0.387
9	104.0	76	0.731
10	116.6	63	0.540
11	342.3	111	0.324
12	320.4	158	0.493
13	272.3	61	0.224
14	357.2	177	0.496
15	116.6	54	0.463
16	488.4	178	0.364
17	193.2	48	0.248
total	4976.2	2029	0.408
standard deviation of area density			0.119

The mass loading of Co SAs anchored on WSe_2 (monolayer and 7 layers) was approximately determined as follows:

$$\text{mass loading}_{\text{Co}}\ (\text{monolayer}) = \frac{58.933 \times 0.408}{(58.933 \times 0.408 + 341.76 \times 9) \times 1} = 0.78\ \text{wt\%}$$

$$\text{mass loading}_{\text{Co}}\ (7\ \text{layers}) = \frac{58.933 \times 0.408}{(58.933 \times 0.408 + 341.76 \times 9) \times 7} = 0.11\ \text{wt\%}$$

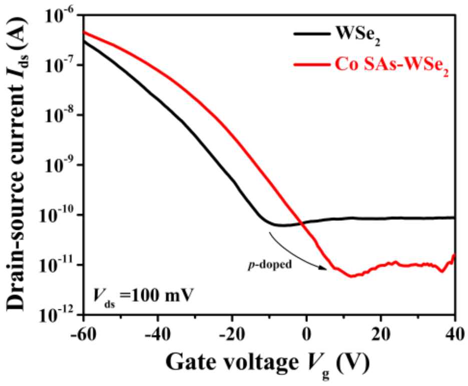

Supplementary Figure 17. Transfer characteristics of mechanically exfoliated WSe_2 (black) and Co SAs-WSe_2 (red).

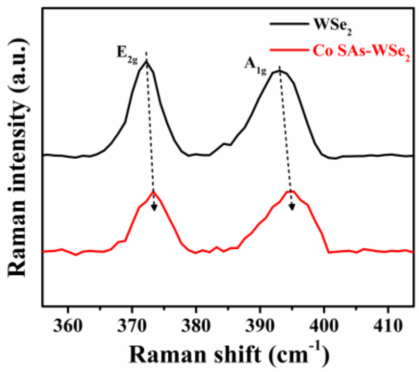

Supplementary Figure 18. Raman spectroscopy of mechanically exfoliated WSe_2 (black) and Co SAs-WSe_2 (red).

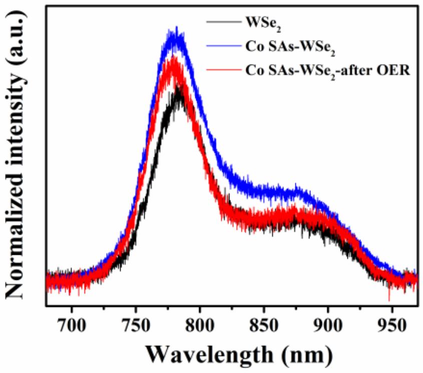

Supplementary Figure 19. PL spectroscopy of mechanically exfoliated WSe_2 (black) and Co SAs-WSe_2 before (blue) and after OER stability test (red).

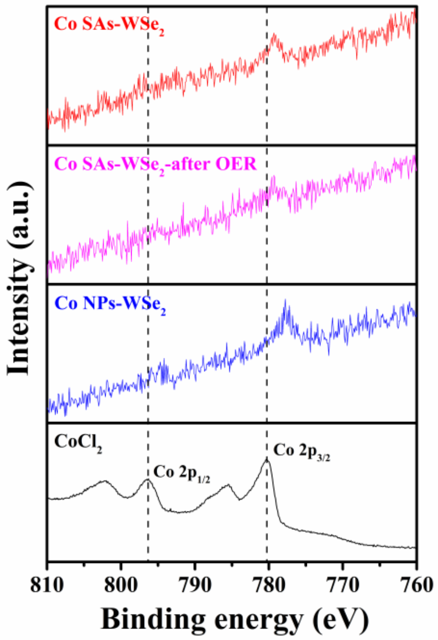

Supplementary Figure 20. XPS spectra showing Co 2p core level peak regions for $CoCl_2$ (black), Co NPs-WSe_2 (blue), Co SAs-WSe_2 before (red) and after OER stability test (magenta).

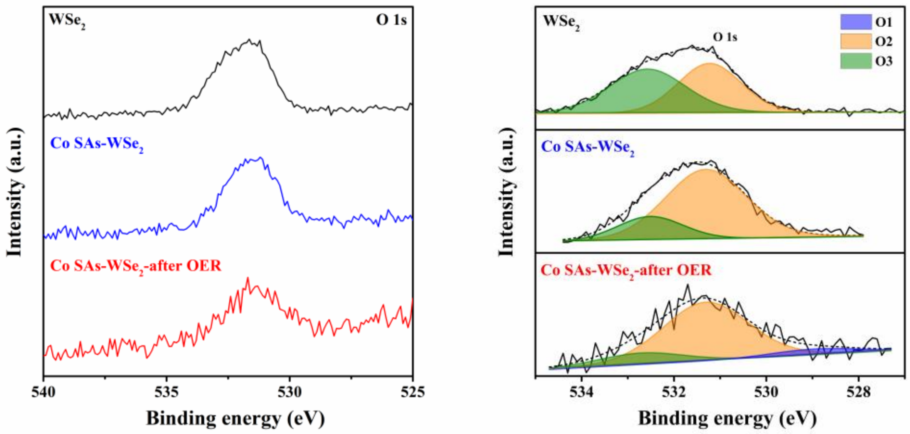

Supplementary Figure 21. XPS spectra (left) showing O 1s core level peak regions for bulk WSe_2 (black) and Co SAs-WSe_2 before (blue) and after OER stability test (red), and corresponding peak deconvolution (right).

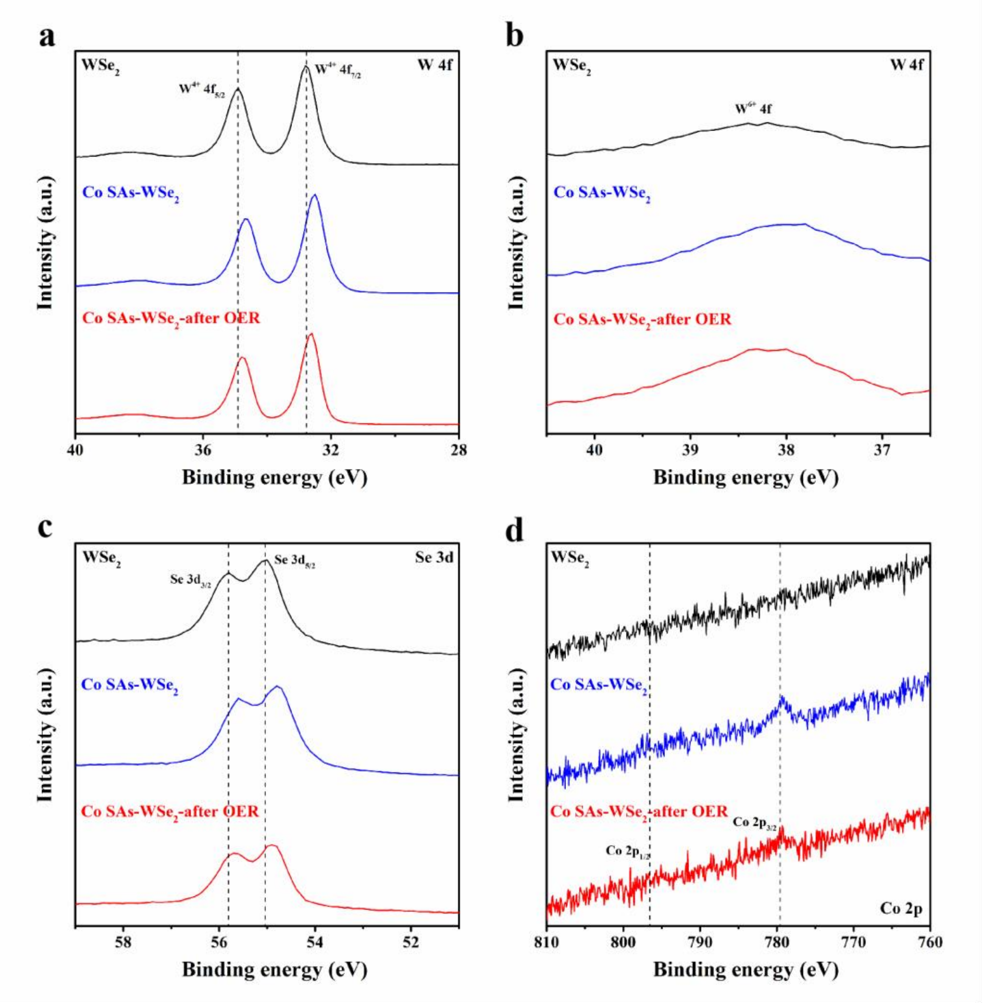

Supplementary Figure 22. XPS spectra showing W 4f (**a**, **b**), Se 3d (**c**) and Co 2p (**d**) core level peak regions for bulk WSe_2 (black) and Co SAs-WSe_2 before (blue) and after OER stability test (red).

Fabrications of micro-device (microcell) & reactor for gas collection

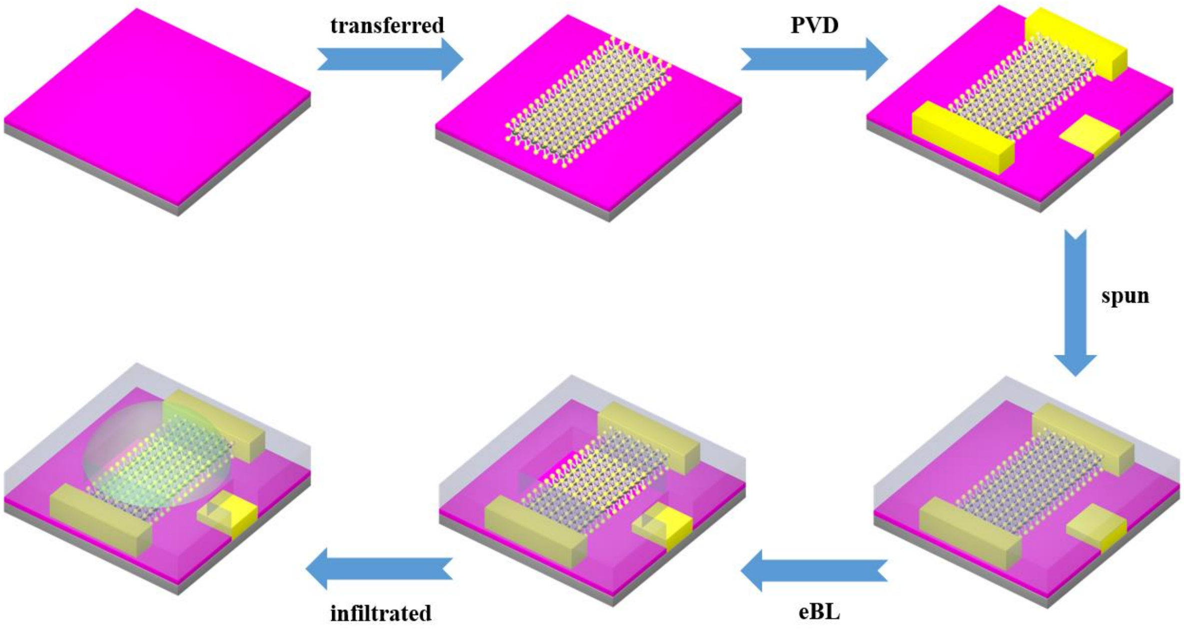

Supplementary Figure 23. Preparation steps of the individual micro-device.

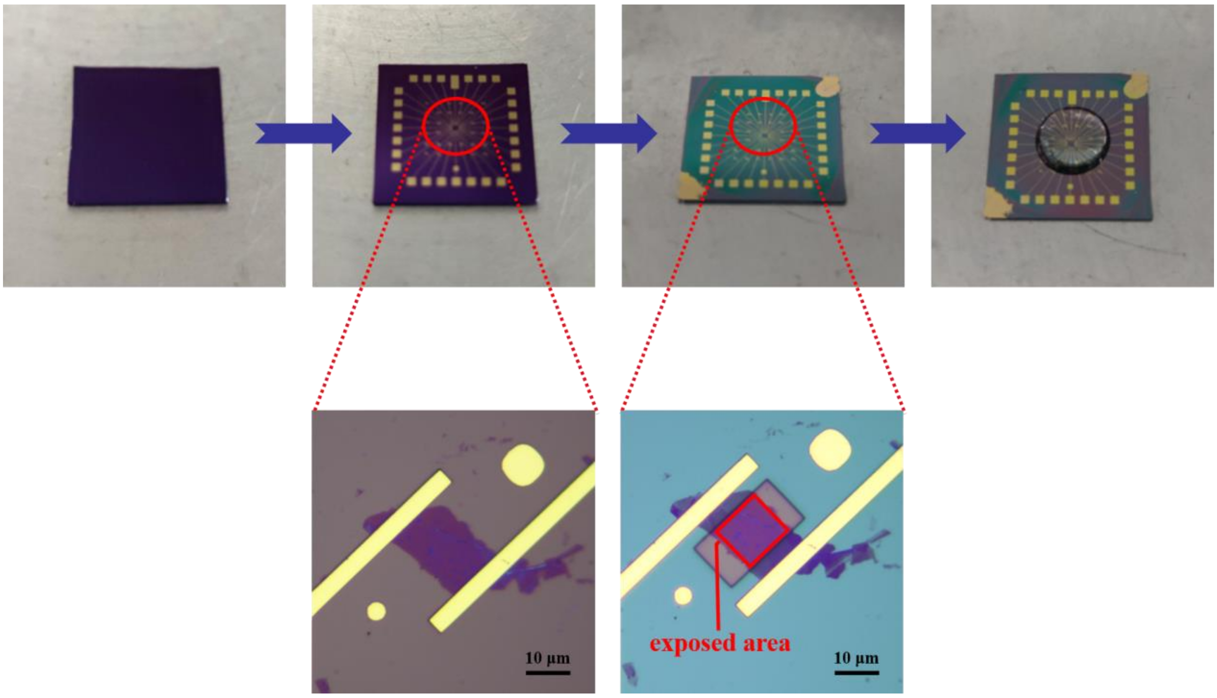

Supplementary Figure 24. Optical images corresponding to the steps shown in **Supplementary Figure 23**.

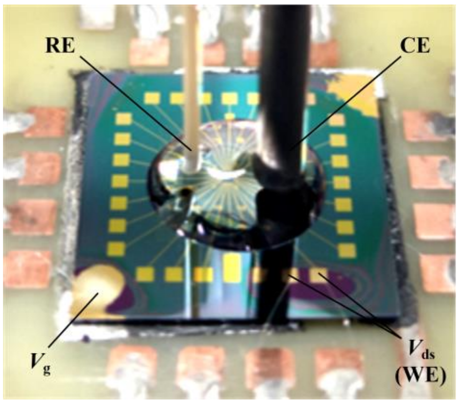

Supplementary Figure 25. The optical image of the four-electrode microcell.

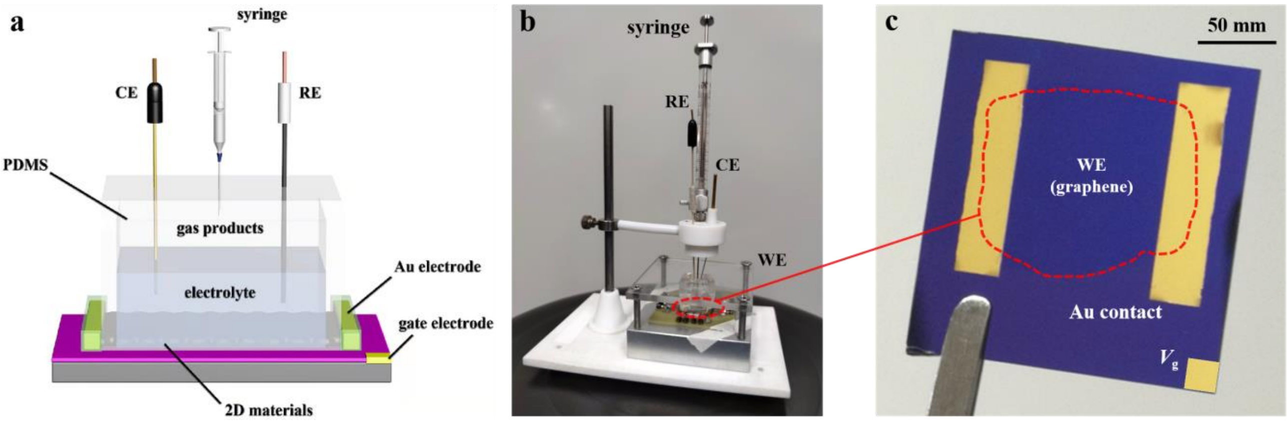

Supplementary Figure 26. The schematic diagram (**a**) and optical images (**b**, **c**) of the electrochemical reactor for gas collection.

Thickness selectivity of MoS_2 on the modulation efficiency of OEEFs

MoS_2 nanosheets with different thicknesses were fabricated into micro-devices for electrochemical measurements (HER) through applied different gate voltages (under OEEF regulation). Specifically, $\Delta\eta_E$ of MoS_2 with 8 layers (6.09 nm, **Supplementary Figure 27g**) is 33 mV (the right of **Supplementary Figure 28**) while monolayer MoS_2 (0.83 nm, **Supplementary Figure 27a**) exhibits much wider tunable range of $\Delta\eta_E$=116 mV (the left of **Supplementary Figure 28**), indicating that electric fields act more effectively on thin MoS_2. As a control, CVD-grown monolayer MoS_2 (0.71 nm, **Supplementary Figure 27h**) was fabricated into micro-device, which exhibited tunable HER performance modulated by OEEFs ($\Delta\eta_E$=80 mV, the left of **Supplementary Figure 29**). And the HER activity of overly-thick MoS_2 (>10 nm, **Supplementary Figure 27i**) show no response to OEEFs (the right of **Supplementary Figure 29**). According to the electronic/electrochemical data from eight mechanically exfoliated MoS_2 samples (**Supplementary Figure 27**), 5 nm (7 layer) is regarded as the threshold of thickness to distinguish the modulation efficiency of thick MoS_2 from thin MoS_2 (as shown in **Fig. 2b** in the manuscript). And we investigated the variation of *in-situ* drain-source current (I_{ds}) and HER overpotential ($|\eta|$) with gate voltages (**Supplementary Figure 30**), further confirming the thickness-selectivity of MoS_2 on the modulation efficiency of OEEFs.

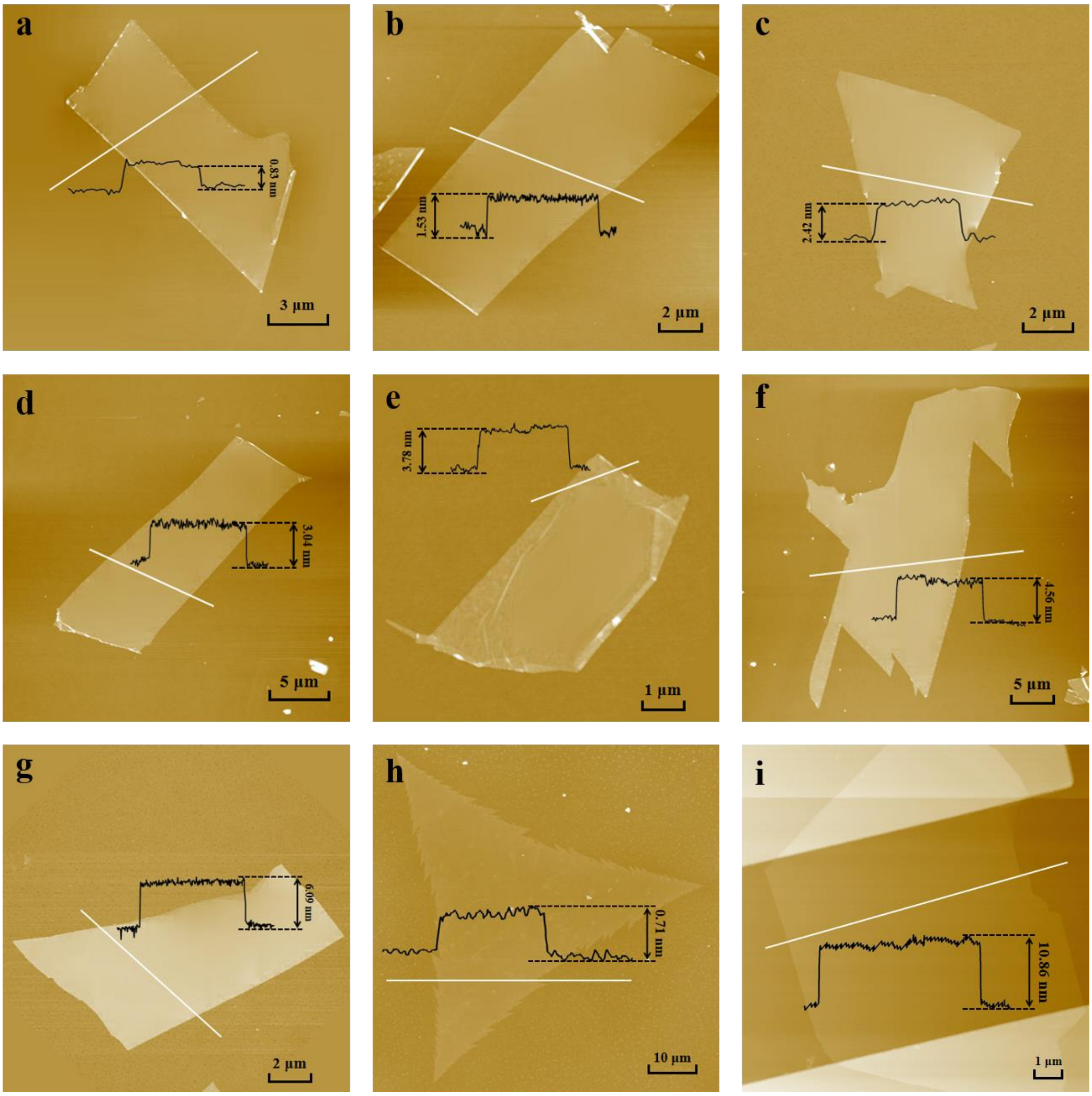

Supplementary Figure 27. AFM images of mechanically exfoliated MoS_2 with different thicknesses (**a-g** and **i**), and CVD-grown monolayer MoS_2 (**h**).

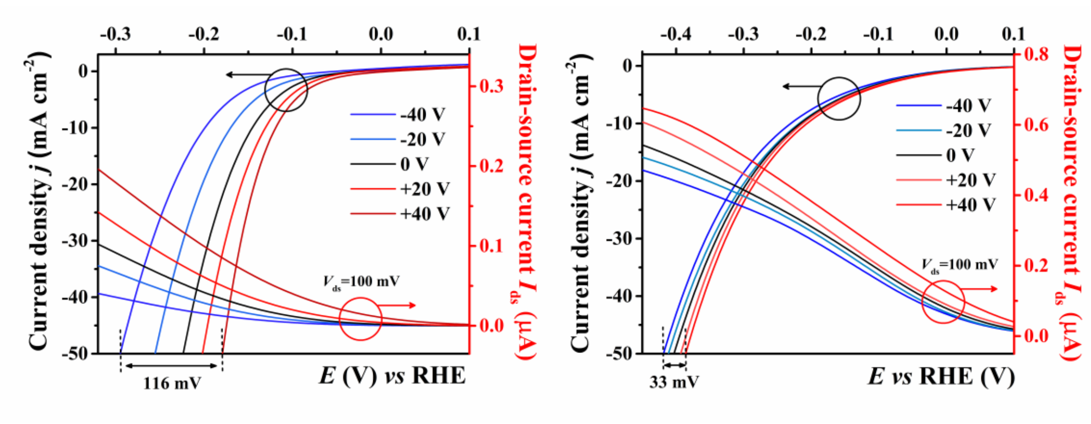

Supplementary Figure 28. HER polarization curves of thin MoS_2 (0.83 nm, left) and thick MoS_2 (6.09 nm, right) under OEEF regulation.

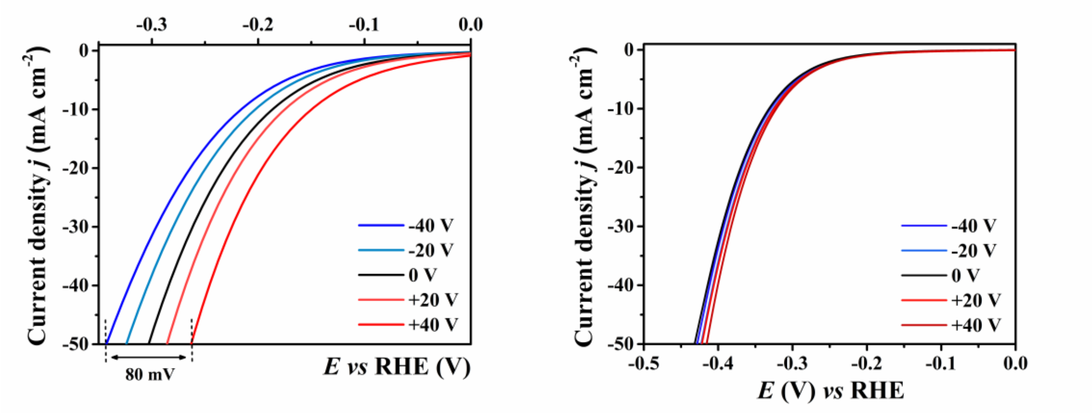

Supplementary Figure 29. HER polarization curves of CVD-grown monolayer MoS_2 (0.71 nm, left) and overly thick MoS_2 (>10 nm, right) under OEEF regulation.

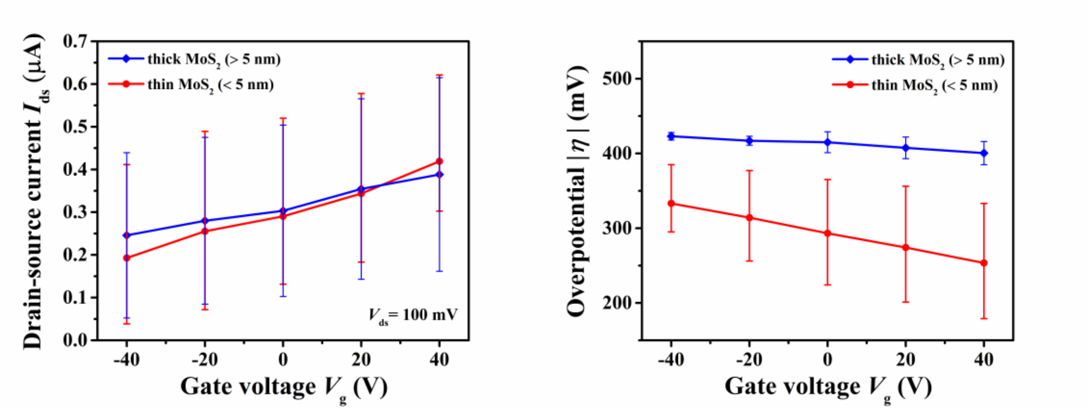

Supplementary Figure 30. The variation of the *in-situ* drain-source current (left, @ E=−0.4 V) and overpotential (right, @ 50 mA cm^{-2}) with gate voltages.

Electrocatalytic measurements under OEEF regulation

In the on-chip electrochemical microcell (reactors), a graphite rod and a leak-free Ag/AgCl electrode (with 3.4 M KCl) was used as the counter electrode (CE) and the reference electrode (RE), respectively. And the working electrode includes pristine 2D materials (MoS_2, WSe_2, graphene) and SACs (Pt SAs, Co SAs, Pd SAs) anchored on 2D materials. Specifically, the thickness of 2D substrates is less than 5 nm and the exposed area of 2D SACs is 20-100 μm^2, which depends on the window patterned by eBL. The pre-activation of working electrode (2D SACs) was operated through CV tests in the potential range (vs. RHE) of −0.1 V to 0.3 V. Usually 20 CV cycles were running to make sure that the electrochemical curves eventually remained unchanged.

We performed a series of electrochemical measurements including CV, LSV and potentiostatic tests in this work. During CV and LSV tests, 0.5 M H_2SO_4 was used for HER and 0.1 KOH was used for OER. Specifically, for pristine 2D substrates (MoS_2, WSe_2, graphene) the potential range (vs. RHE) is −0.5 V to 0.1 V in HER tests and 1.0 V to 2.0 V in OER tests. For 2D SACs, the potential range (vs. RHE) is −0.3 V to 0.1 V in HER tests and 1.0 V to 1.8 V in OER tests. During potentiostatic tests, 0.5 M H_2SO_4 was used for HER and 0.1 KOH was used for OER. Specifically, for pristine large-size graphene the applied potential (vs. RHE) is −0.3 V in HER tests and 1.8 V in OER tests. For SACs anchored on graphene, the applied potential (vs. RHE) is −0.1 V in HER tests and 1.6 V in OER tests.

HER performance of MoS_2 and Pt SAs-MoS_2 under OEEF regulation.

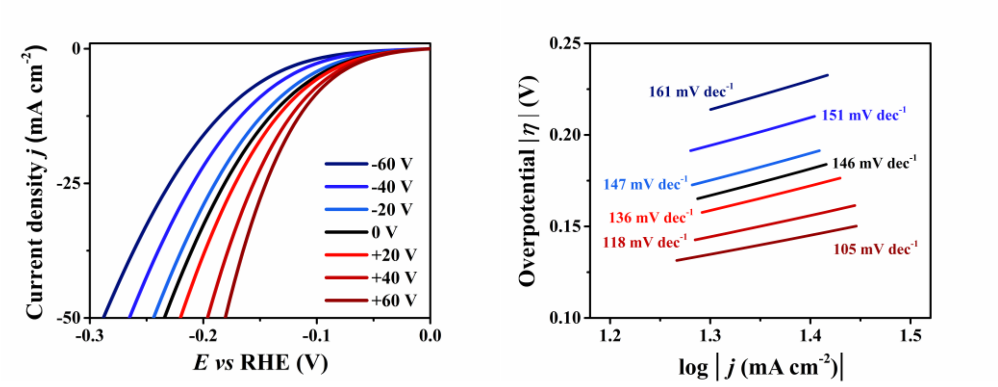

Supplementary Figure 31. HER polarization curves (left) and Tafel plots (right) of MoS_2 under OEEF regulation.

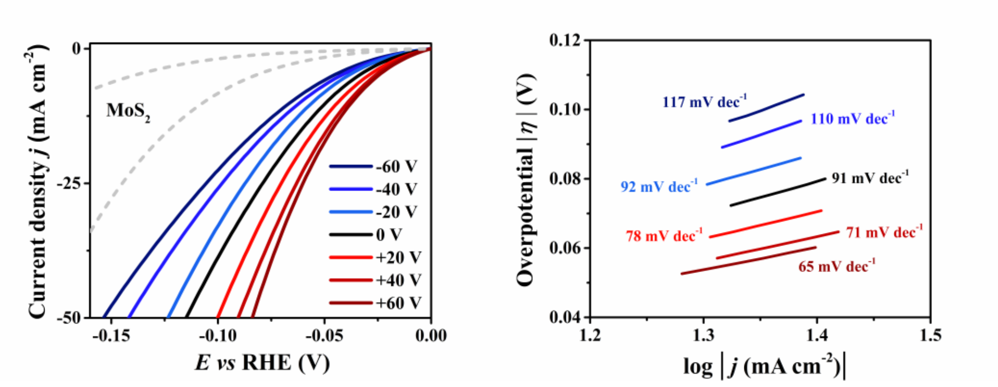

Supplementary Figure 32. HER polarization curves (left) and Tafel plots (right) of Pt SAs-MoS_2 under OEEF regulation.

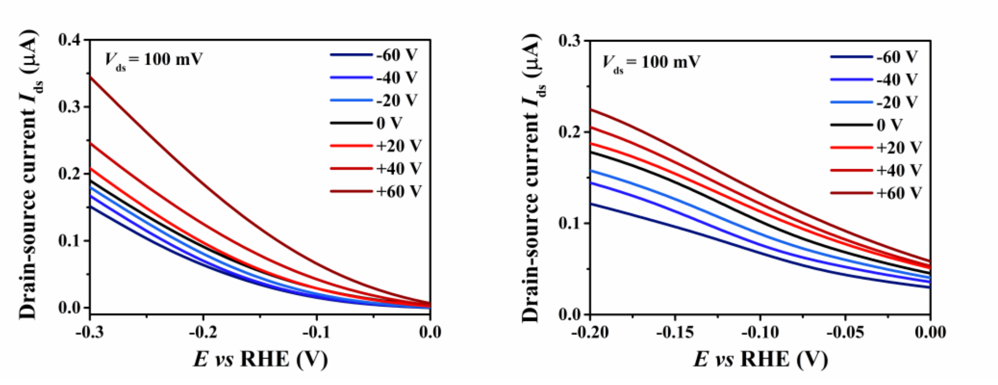

Supplementary Figure 33. The *in-situ* drain-source current (V_{ds}=100 mV) of MoS_2 (left) and Pt SAs-MoS_2 (right) acquired during the HER process.

HER and OER performance of WSe_2 and Pt SAs-WSe_2 under OEEF regulation. 1.46 mg K_2PtCl_6 (99.95 %, *Admas-beta*) was added into 30 mL deionized water and ethylene glycol mixed solution (9:1 volume ratio). Then the 2D WSe_2 were immersed in it under 365 nm UV radiation for 2 h, which was taken out to heat at 150 °C for 15 min. The concentration of K_2PtCl_6 was 0.1 mmol L^{-1}.

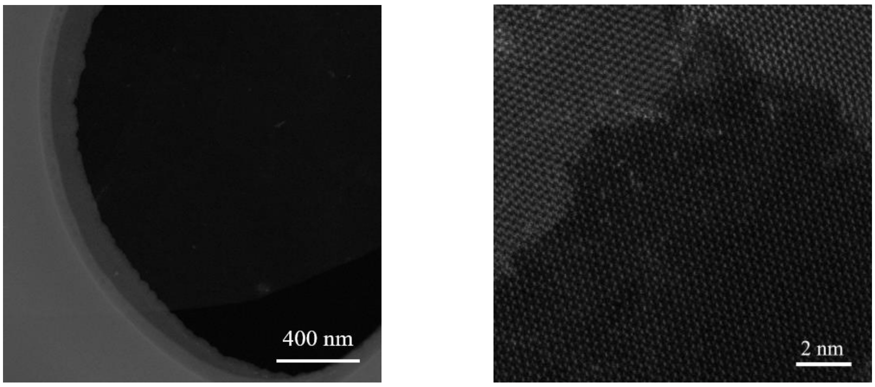

Supplementary Figure 34. HAADF-STEM images of Pt SAs-WSe_2.

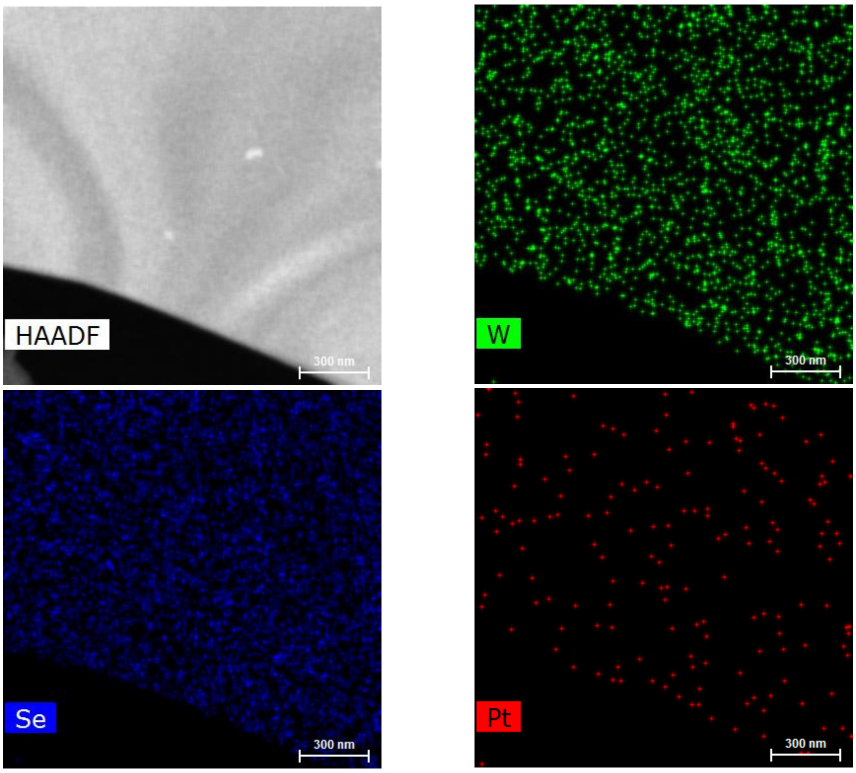

Supplementary Figure 35. STEM-EDS elemental mappings (W, Se and Pt) of Pt SAs-WSe_2.

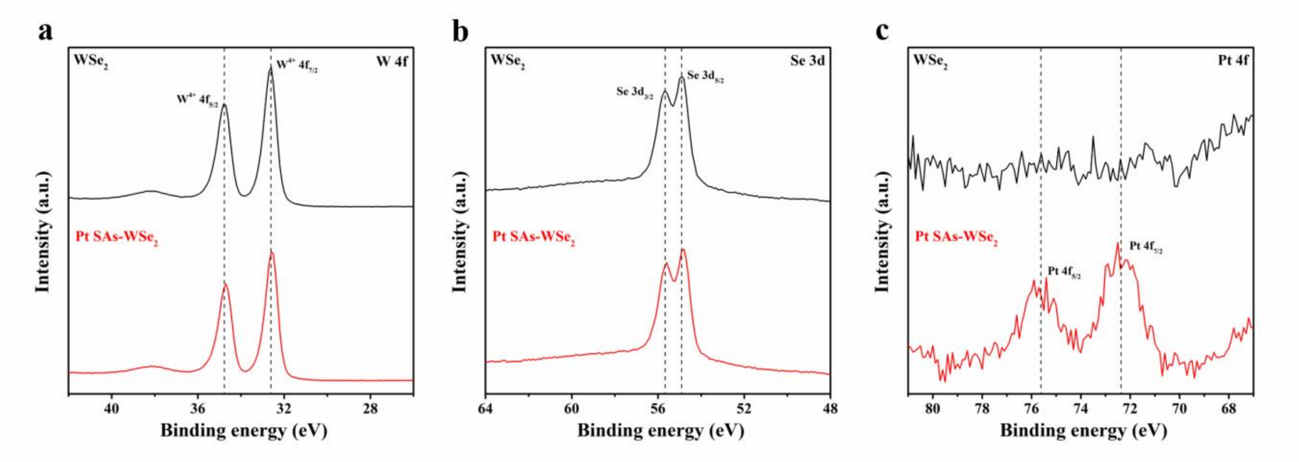

Supplementary Figure 36. XPS spectra showing W 4f (**a**), Se 3d (**b**) and Pt 4f (**c**) core level peak regions for bulk WSe_2 (black) and Pt SAs-WSe_2 (red).

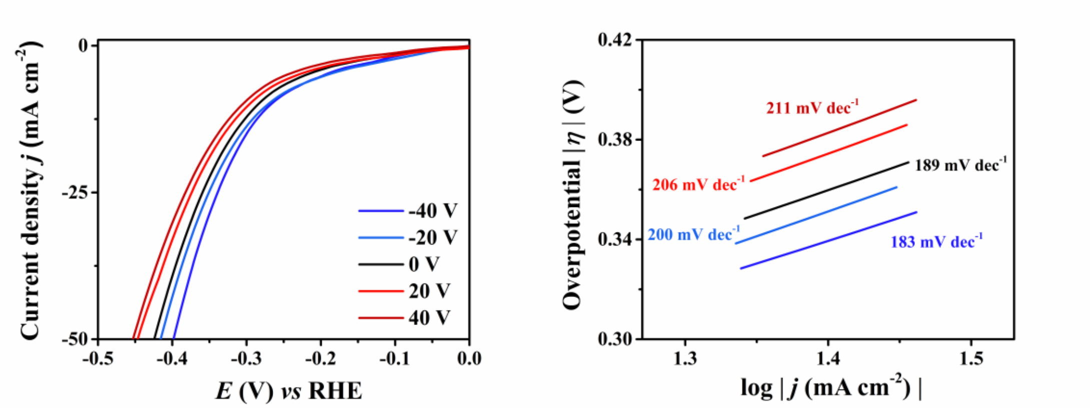

Supplementary Figure 37. HER polarization curves (left) and Tafel plots (right) of WSe_2 under OEEF regulation.

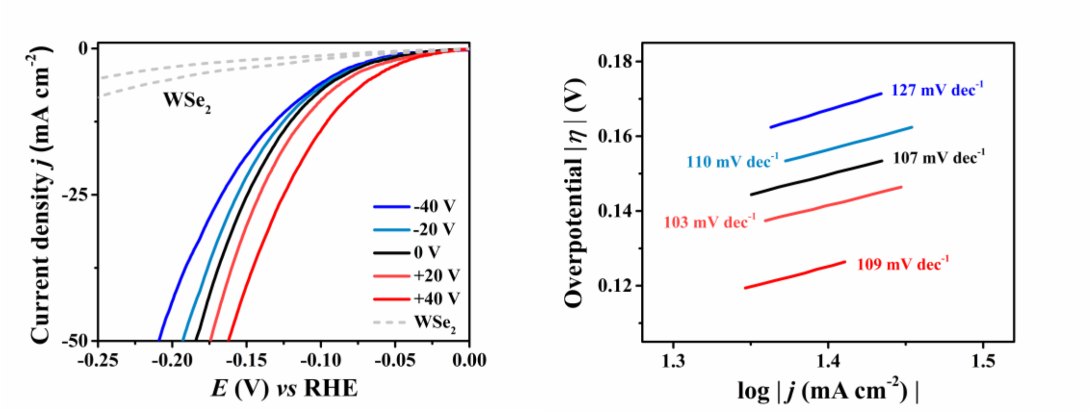

Supplementary Figure 38. HER polarization curves (left) and Tafel plots (right) of Pt SAs-WSe_2 under OEEF regulation.

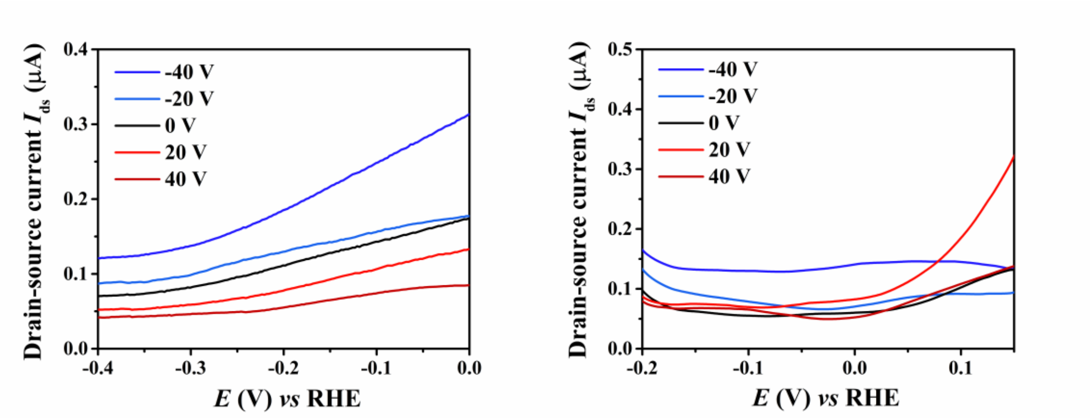

Supplementary Figure 39. The *in-situ* drain-source current (V_{ds}=100 mV) of WSe_2 (left) and Pt SAs-WSe_2 (right) acquired during the HER process.

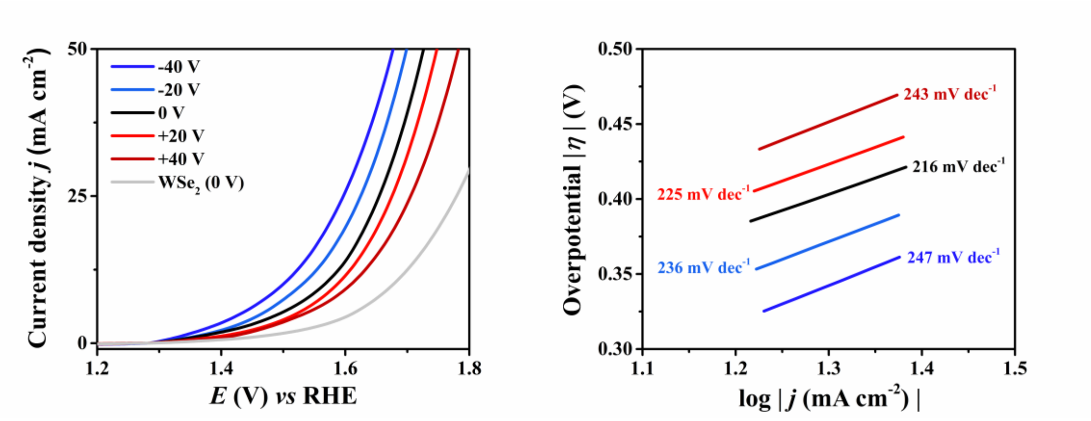

Supplementary Figure 40. OER polarization curves (left) and Tafel plots (right) of Pt SAs-WSe_2 under OEEF regulation.

HER performance of graphene and Pt SAs-graphene under OEEF regulation. 1.46 mg K_2PtCl_6 (99.95 %, *Admas-beta*) was added into 30 mL deionized water and ethylene glycol mixed solution. Then the 2D graphene was immersed in it under 365 nm UV radiation for 2 h, which was taken out to heat at 150 °C for 15 min. The concentration of K_2PtCl_6 was 0.1 mmol L^{-1}.

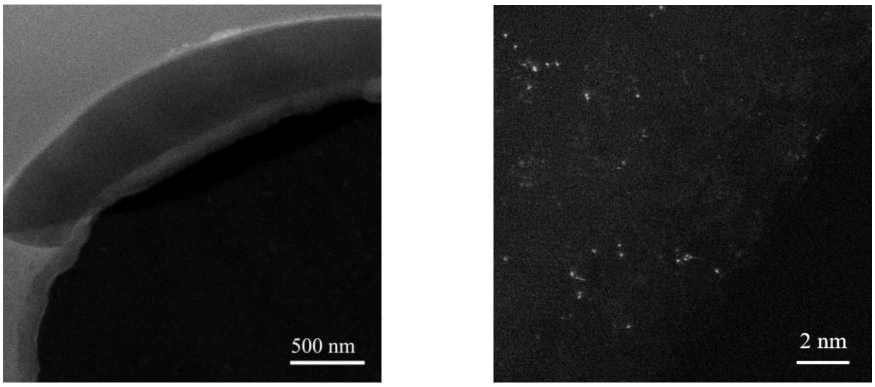

Supplementary Figure 41. HAADF-STEM images of Pt SAs-graphene.

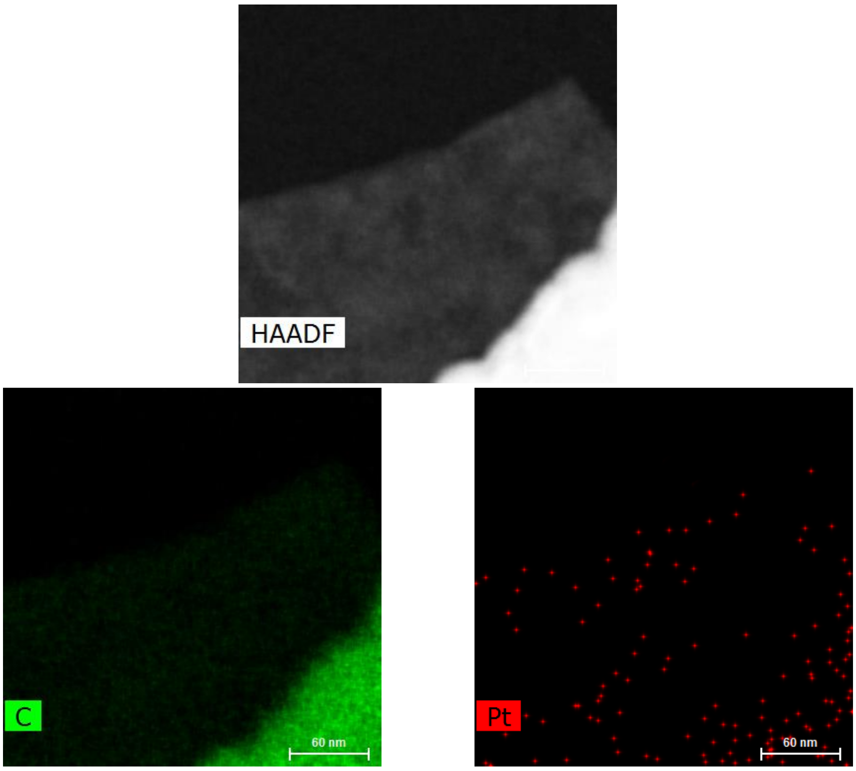

Supplementary Figure 42. STEM-EDS elemental mappings (C and Pt) of Pt SAs-graphene.

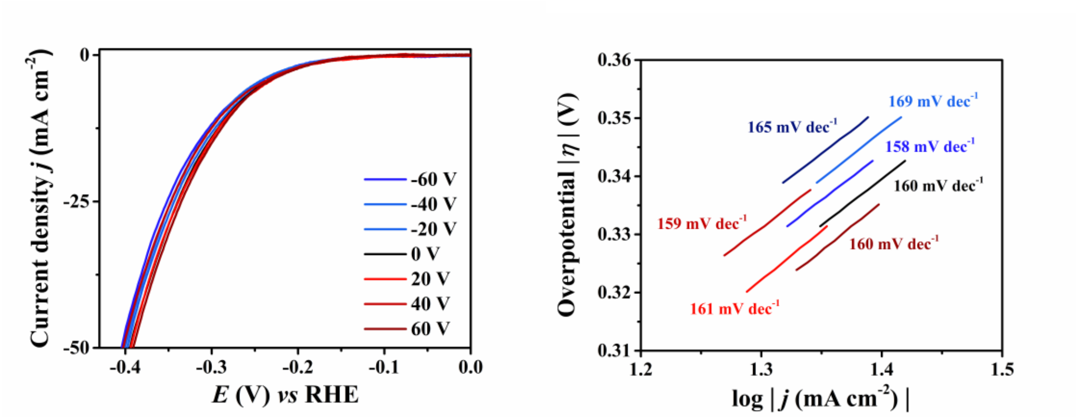

Supplementary Figure 43. HER polarization curves (left) and Tafel plots (right) of mechanically exfoliated graphene under OEEF regulation.

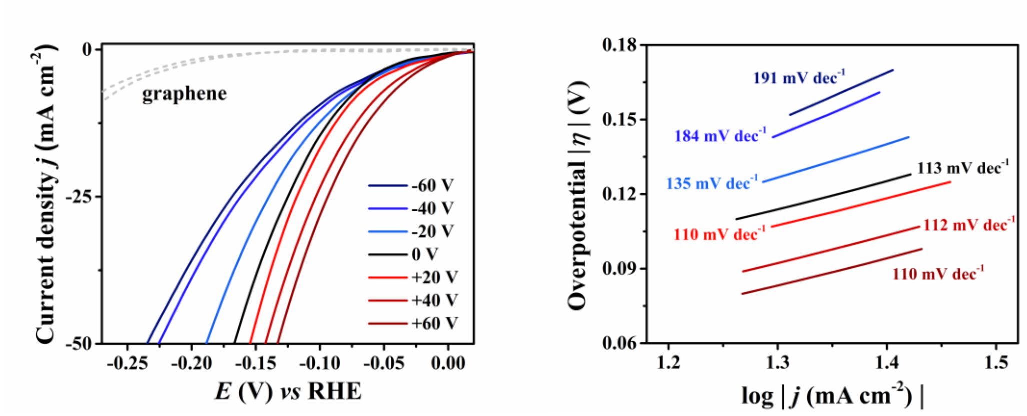

Supplementary Figure 44. HER polarization curves (left) and Tafel plots (right) of Pt SAs-graphene under OEEF regulation.

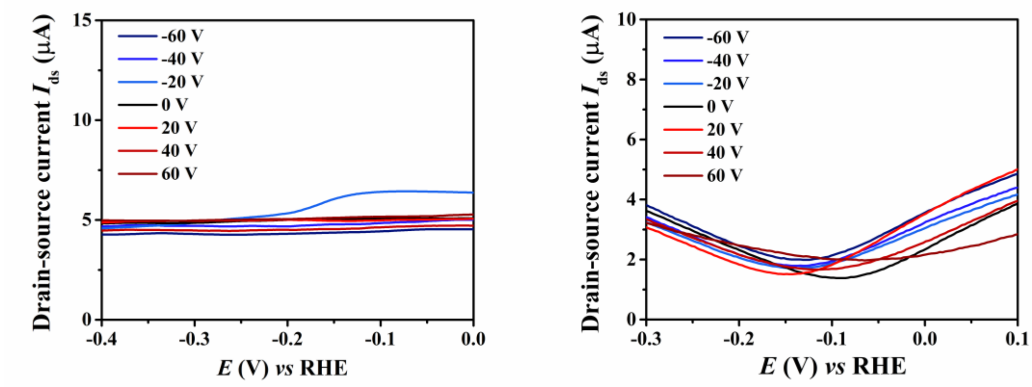

Supplementary Figure 45. The *in-situ* drain-source current (V_{ds}=10 mV) of graphene (left) and Pt SAs-graphene (right) acquired during the HER process.

HER performance of Pd SAs-MoS_2 under OEEF regulation. 1.96 mg K_2PdCl_4 (99%+, *Admas-beta*) was added into 30 mL deionized water and ethylene glycol mixed solution. Then the 2D MoS_2 was immersed in it under 365 nm UV radiation for 2 h, which was taken out to heat at 150 °C for 15 min. The concentration of K_2PdCl_4 was 0.2 mmol L^{-1}.

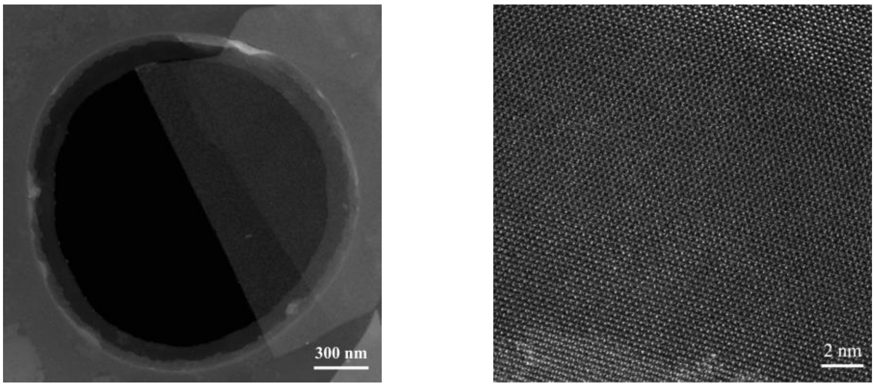

Supplementary Figure 46. HAADF-STEM images of Pd SAs-MoS_2.

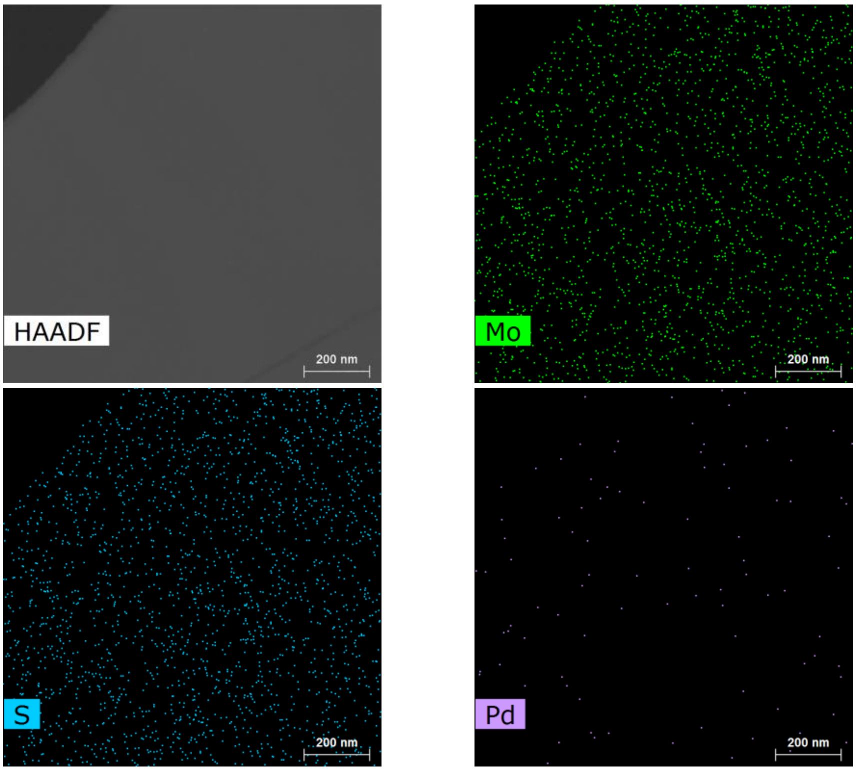

Supplementary Figure 47. STEM-EDS elemental mappings (Mo, S and Pd) of Pd SAs-MoS_2.

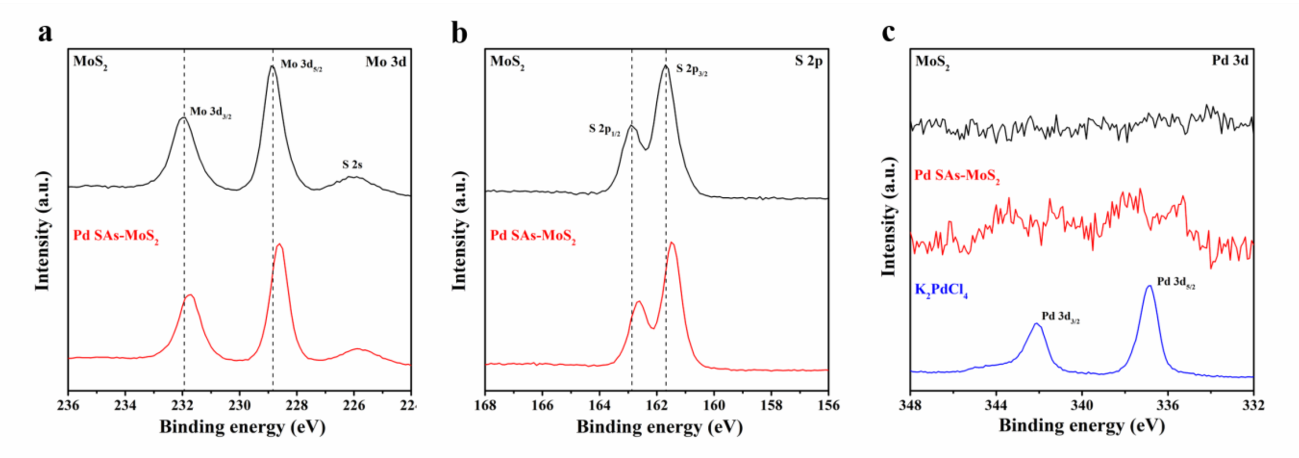

Supplementary Figure 48. XPS spectra showing Mo 3d (**a**), S 2p (**b**) and Pd 3d (**c**) core level peak regions for bulk MoS_2 (black) and Pd SAs-MoS_2 (red). K_2PdCl_4 was used as the Pd^{2+} reference.

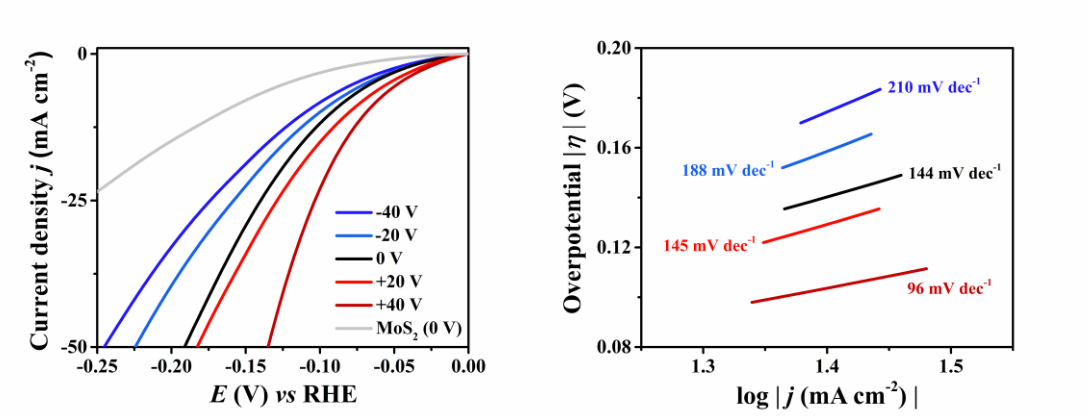

Supplementary Figure 49. HER polarization curves (left) and Tafel plots (right) of Pd SAs-MoS_2 under OEEF regulation.

HER performance of Pt NPs-MoS_2 under OEEF regulation. Electrodeposition of Pt nanoparticles on MoS_2 was conducted using a linear sweep voltammetry method. A layer of PMMA film was spun on the silicon wafer as the passivation layer, in which a window designed by e-beam lithography was etched by acetone to expose part of MoS_2. A platinum wire was used as the counter electrode and an Ag/AgCl electrode was used as the reference electrode. Then the electrochemical deposition was carried out from 0 V to −0.5 V (*vs.* RHE) in the electrolyte containing 10 mmol L^{-1} H_2PtCl_6 and 0.5 M H_2SO_4. Finally, we got the Pt NPs (~30 nm) on MoS_2.

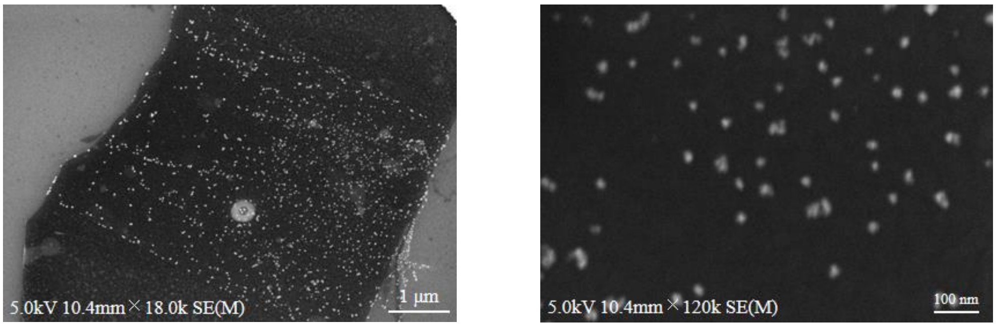

Supplementary Figure 50. Magnified SEM images of Pt NPs-MoS_2.

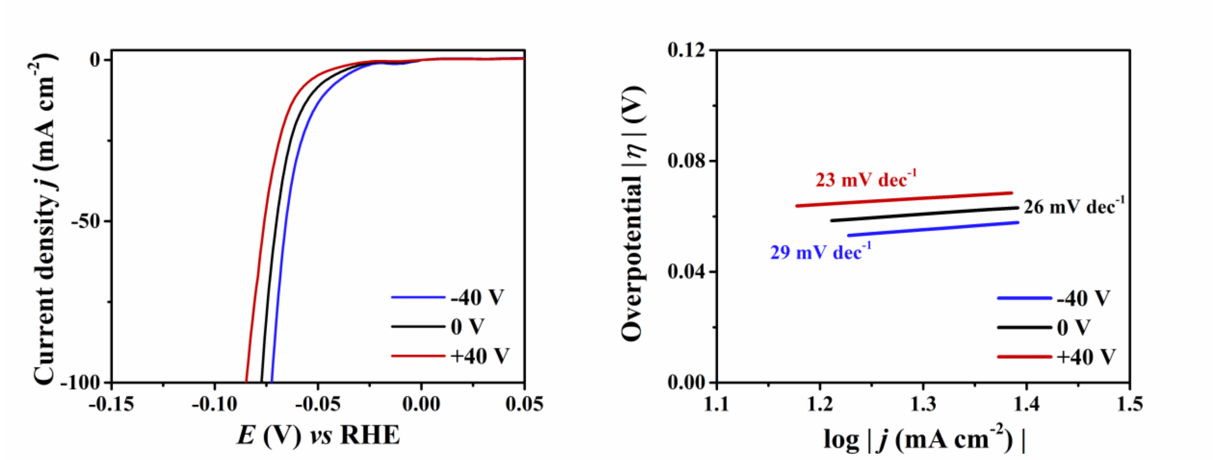

Supplementary Figure 51. HER polarization curves (left) and Tafel plots (right) of Pt NPs-MoS_2 under OEEF regulation.

HER performance of 30 nm-thickness Pt film under OEEF regulation. The silicon wafer with prepatterned Au electrodes was spun with a layer of PMMA, patterned by e-beam lithography, and deposited with Pt film (30 nm) through physical evaporation, successively. After lifting off the layer of PMMA with acetone, the 30 nm-thickness Pt film (75 μm × 8 μm) was prepared. A new PMMA film was then spun on the silicon wafer as the passivation layer, in which a window was etched to expose part of Pt film with fixed area (20 μm × 8 μm). Above all, the individual Pt microelectrode was fabricated for HER test.

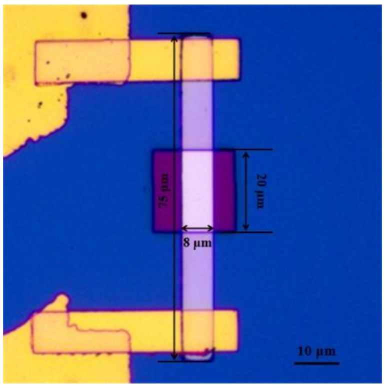

Supplementary Figure 52. Optical image of 30 nm-thickness Pt film.

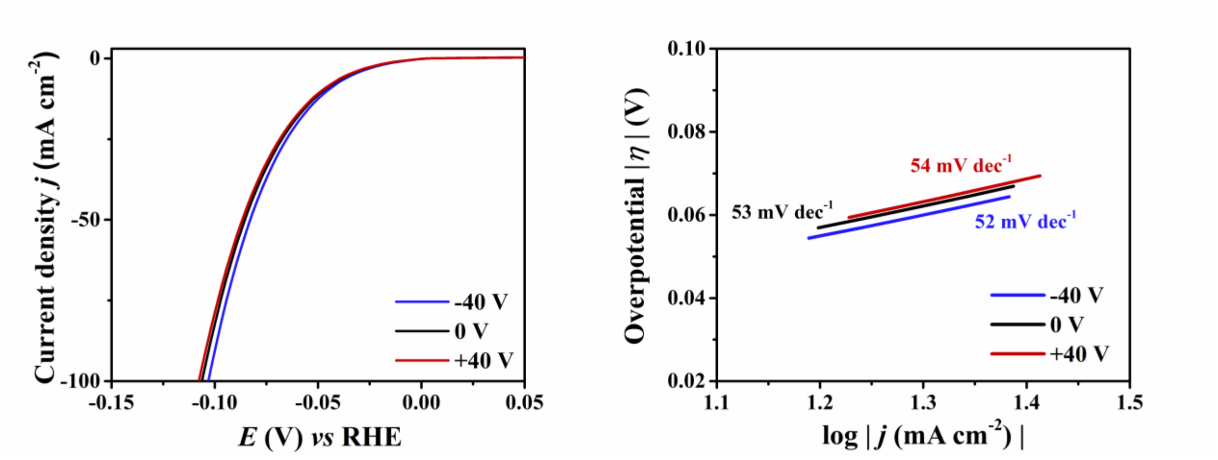

Supplementary Figure 53. HER polarization curves (left) and Tafel plots (right) of Pt microelectrode under OEEF regulation.

OER performance of WSe_2 and Co SAs-WSe_2 under OEEF regulation.

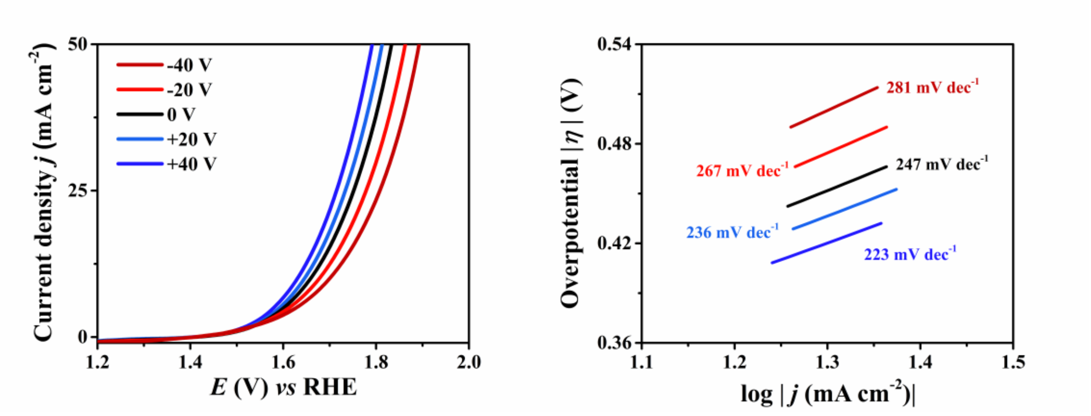

Supplementary Figure 54. OER polarization curves (left) and Tafel plots (right) of WSe_2 under OEEF regulation.

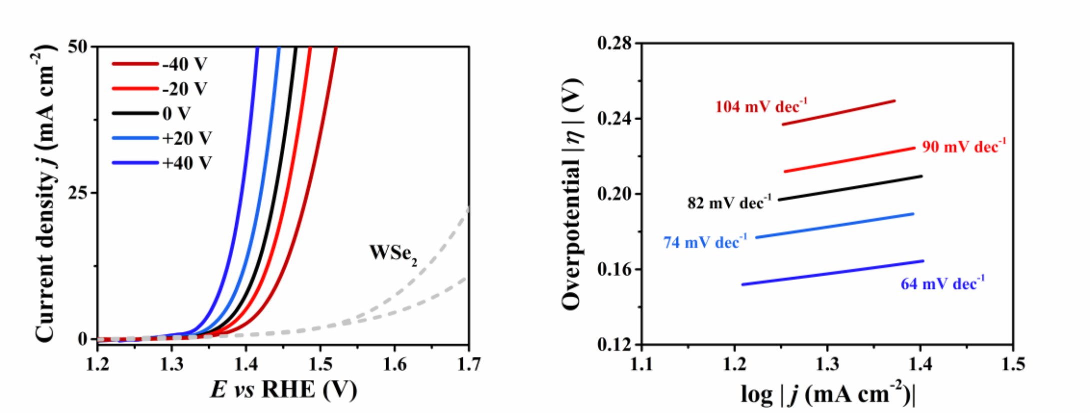

Supplementary Figure 55. OER polarization curves (left) and Tafel plots (right) of Co SAs-WSe_2 under OEEF regulation.

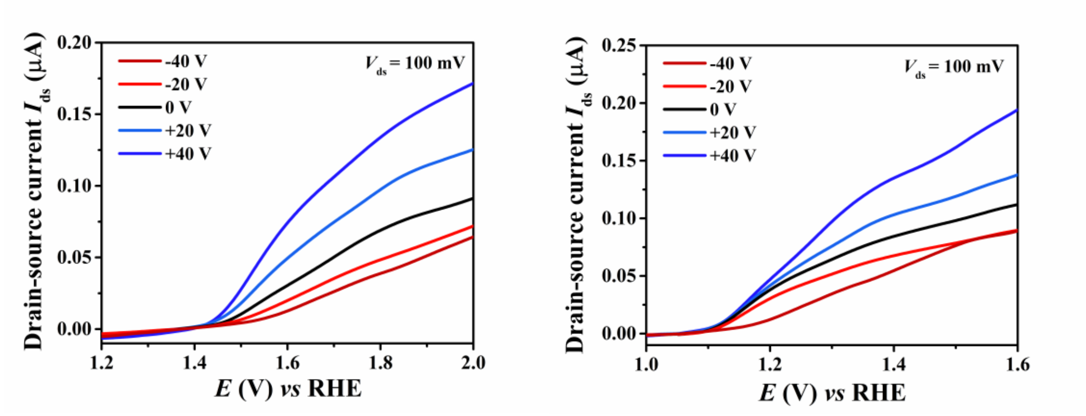

Supplementary Figure 56. The *in-situ* drain-source current (V_{ds}=100 mV) of WSe_2 (left) and Co SAs-WSe_2 (right) acquired during the OER process.

HER and OER performance of MoS_2 and Co SAs-MoS_2 under OEEF regulation. 3.57 mg $CoCl_2 \cdot 6H_2O$ (AR, *Aladdin*) was added into 30 mL deionized water and ethylene glycol mixed solution (9:1 volume ratio). Then the 2D MoS_2 was immersed in it under 365 nm UV radiation for 3 h, which was taken out to heat at 150 °C for 15 min. The concentration of $CoCl_2$ was 0.5 mmol L^{-1}.

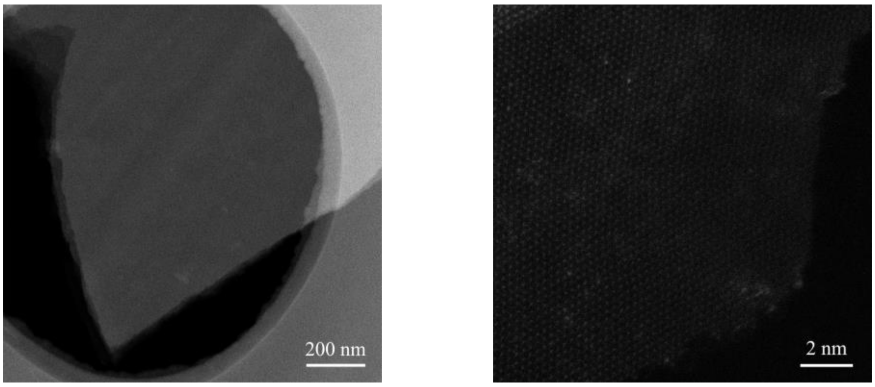

Supplementary Figure 57. HAADF-STEM images of Co SAs-MoS_2.

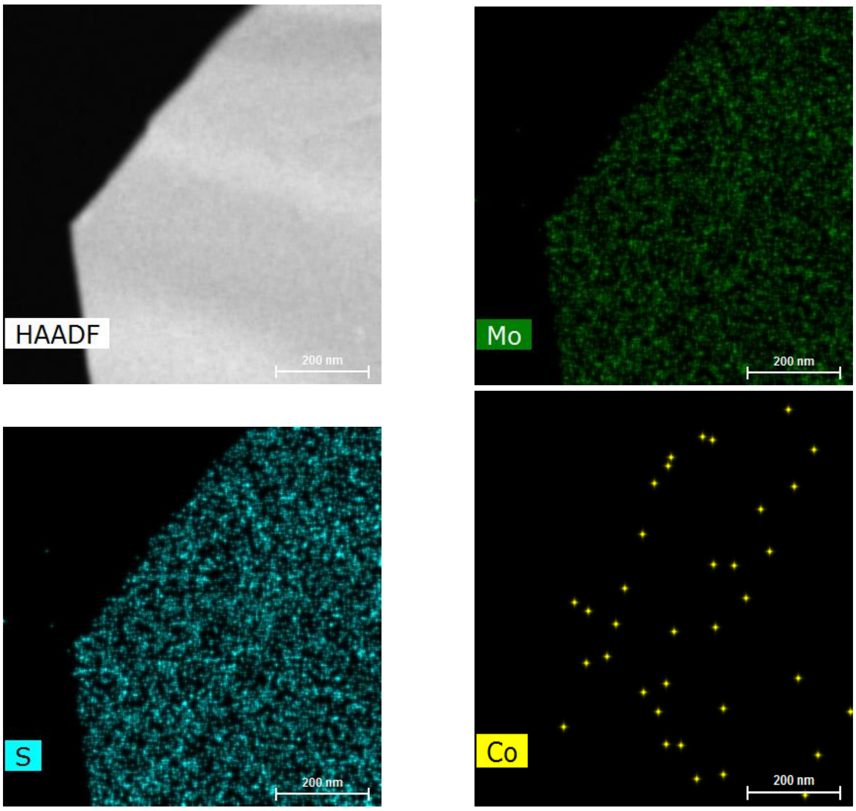

Supplementary Figure 58. STEM-EDS elemental mappings (Mo, S and Co) of Co SAs-MoS_2.

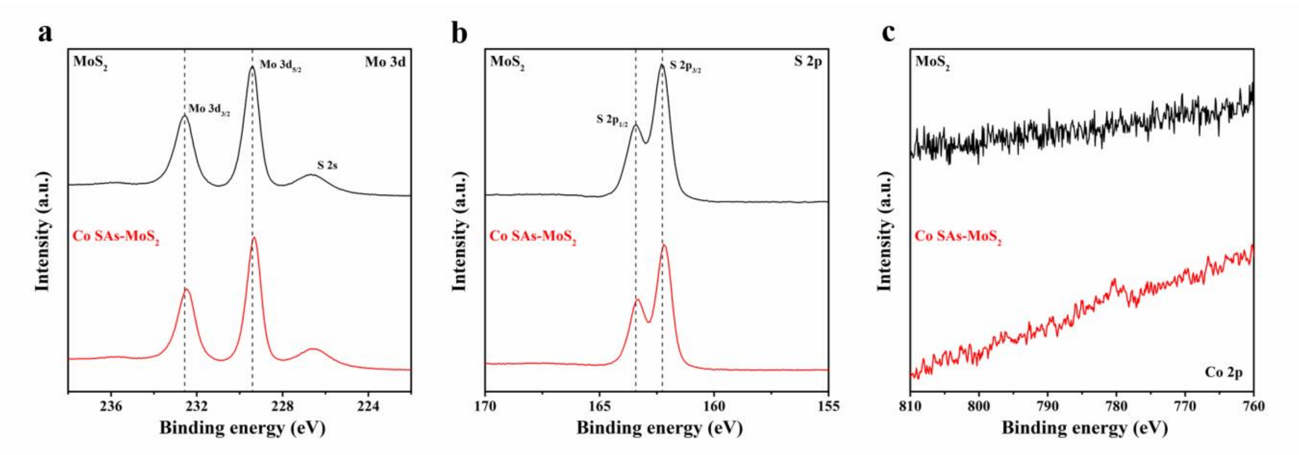

Supplementary Figure 59. XPS spectra showing Mo 3d (**a**), S 2p (**b**) and Co 2p (**c**) core level peak regions for bulk MoS_2 (black) and Co SAs-MoS_2 (red).

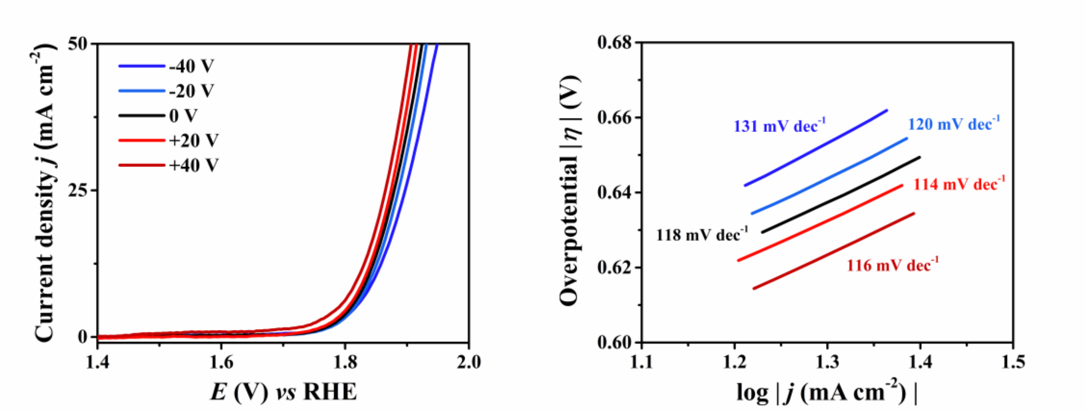

Supplementary Figure 60. OER polarization curves (left) and Tafel plots (right) of MoS_2 under OEEF regulation.

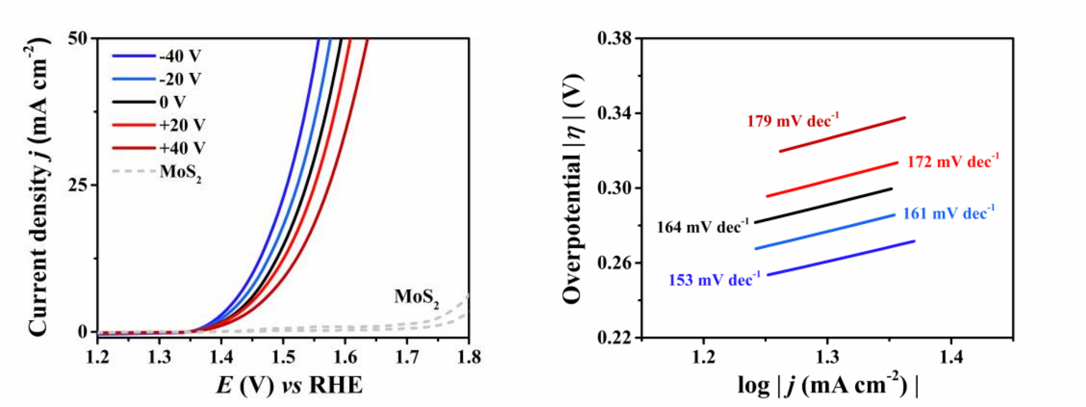

Supplementary Figure 61. OER polarization curves (left) and Tafel plots (right) of Co SAs-MoS_2 under OEEF regulation.

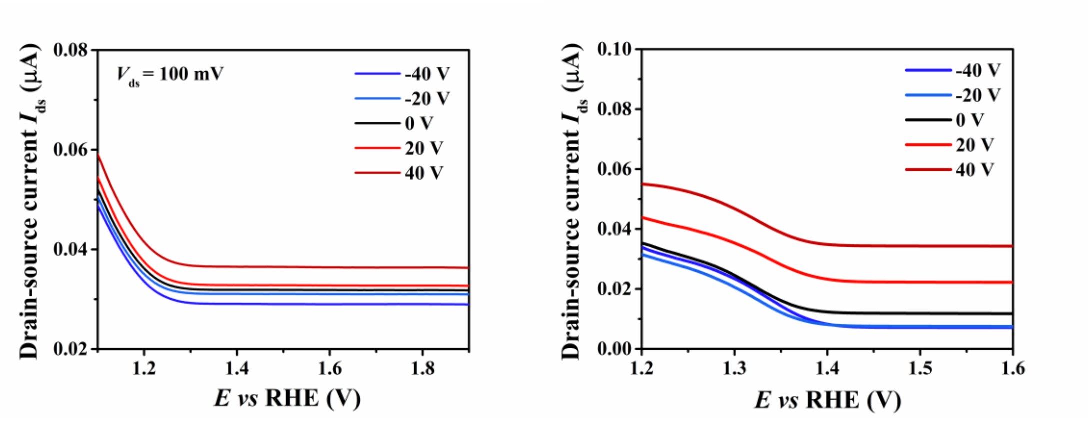

Supplementary Figure 62. The *in-situ* drain-source current (V_{ds}=100 mV) of MoS_2 (left) and Co SAs-MoS_2 (right) acquired during the OER process.

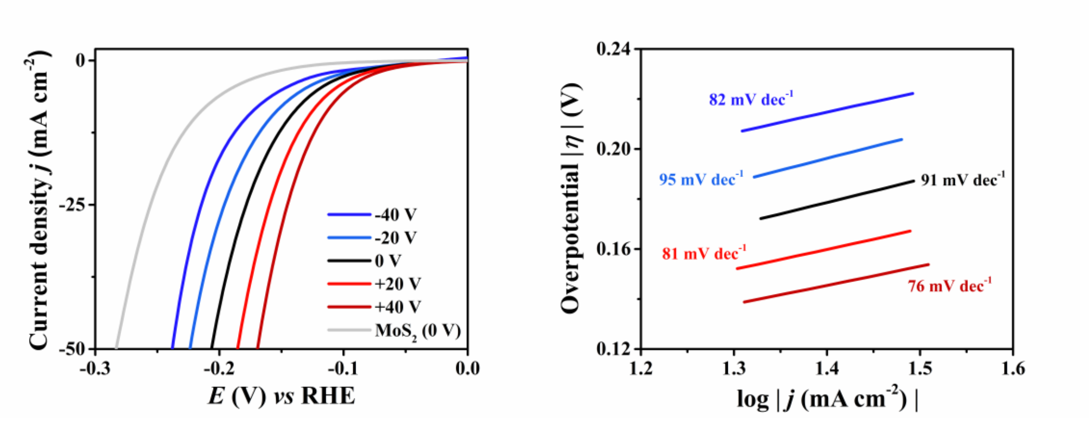

Supplementary Figure 63. HER polarization curves (left) and Tafel plots (right) of Co SAs-MoS_2 under OEEF regulation.

OER performance of graphene and Co SAs-graphene under OEEF regulation. 1.43 mg $CoCl_2 \cdot 6H_2O$ (AR, *Aladdin*) was added into 30 mL deionized water and ethylene glycol mixed solution (9:1 volume ratio). Then the 2D graphene was immersed in it under 365 nm UV radiation for 3 h, which was taken out to heat at 150 °C for 15 min. The concentration of $CoCl_2$ was 0.5 mmol L^{-1}.

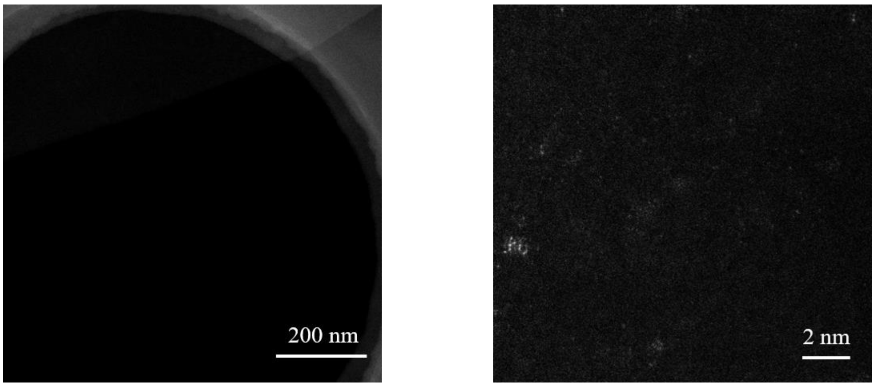

Supplementary Figure 64. HAADF-STEM images of Co SAs-graphene.

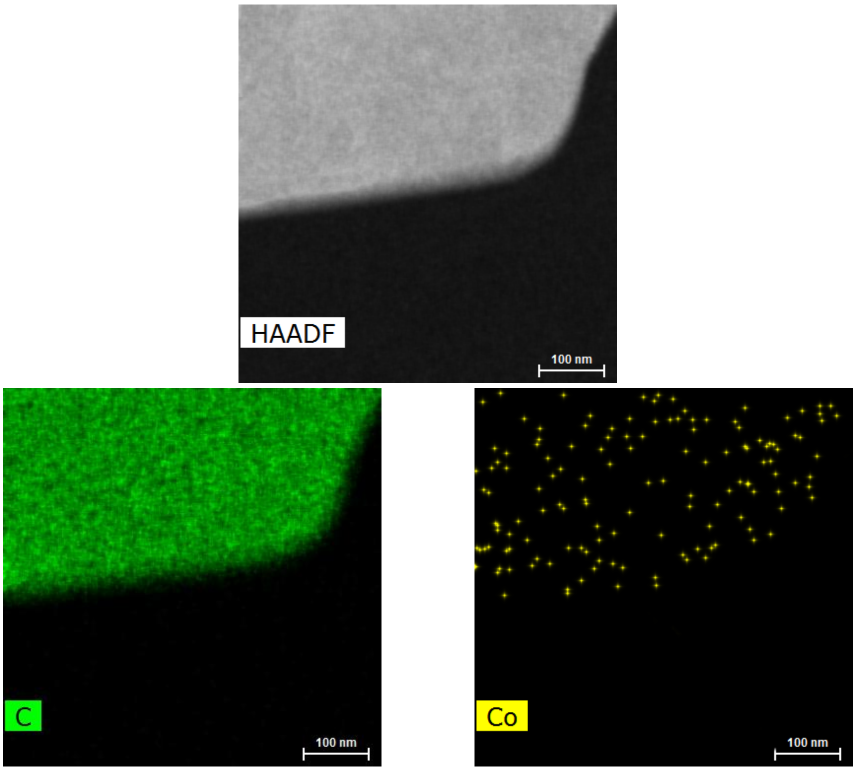

Supplementary Figure 65. STEM-EDS elemental mappings (C and Co) of Co SAs-graphene.

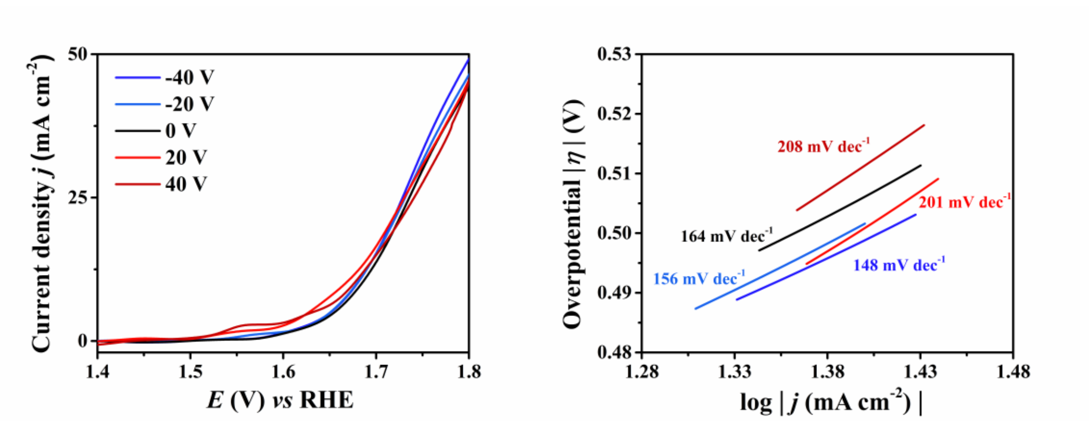

Supplementary Figure 66. OER polarization curves (left) and Tafel plots (right) of mechanically exfoliated graphene under OEEF regulation.

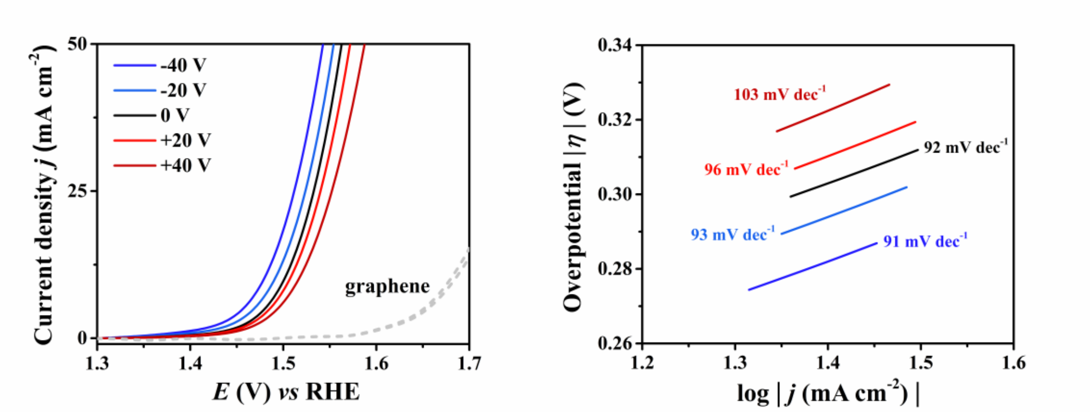

Supplementary Figure 67. OER polarization curves (left) and Tafel plots (right) of Co SAs-graphene under OEEF regulation.

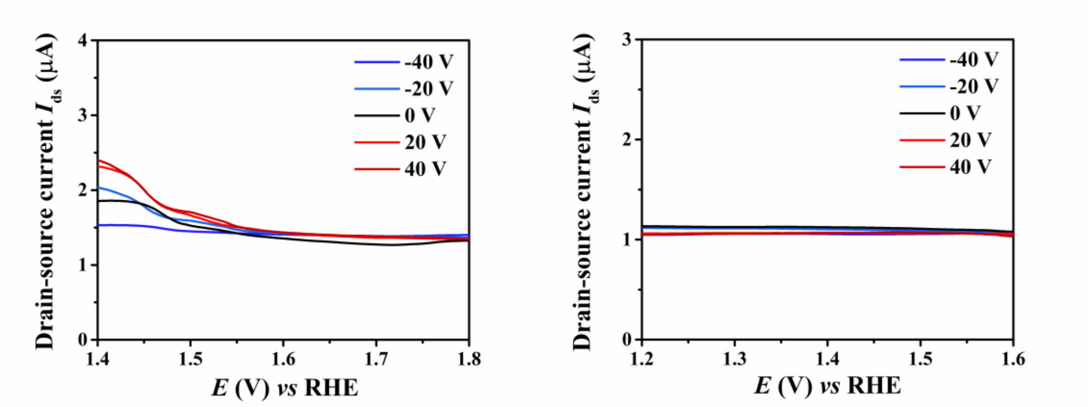

Supplementary Figure 68. The *in-situ* drain-source current (V_{ds}=10 mV) of graphene (left) and Co SAs-graphene (right) acquired during the OER process.

OER performance of Pd SAs-WSe_2 under OEEF regulation. 1.96 mg K_2PdCl_4 (99%+, *Admas-beta*) was added into 30 mL deionized water and ethylene glycol mixed solution. Then the 2D WSe_2 was immersed in it under 365 nm UV radiation for 2 h, which was taken out to heat at 150 °C for 15 min. The concentration of K_2PdCl_4 was 0.2 mmol L^{-1}.

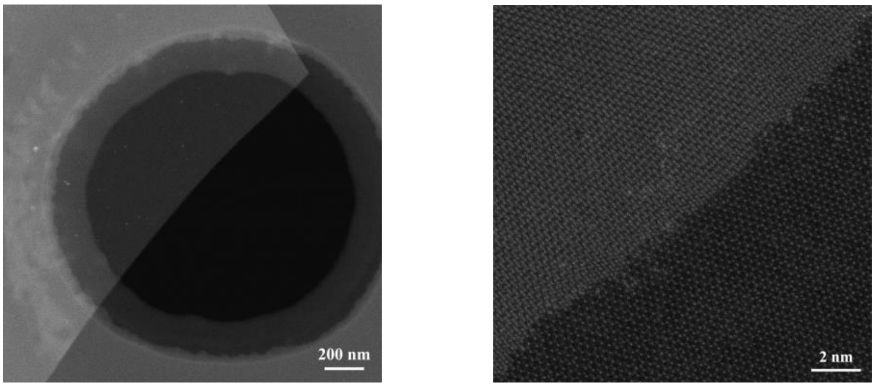

Supplementary Figure 69. HAADF-STEM images of Pd SAs-WSe_2.

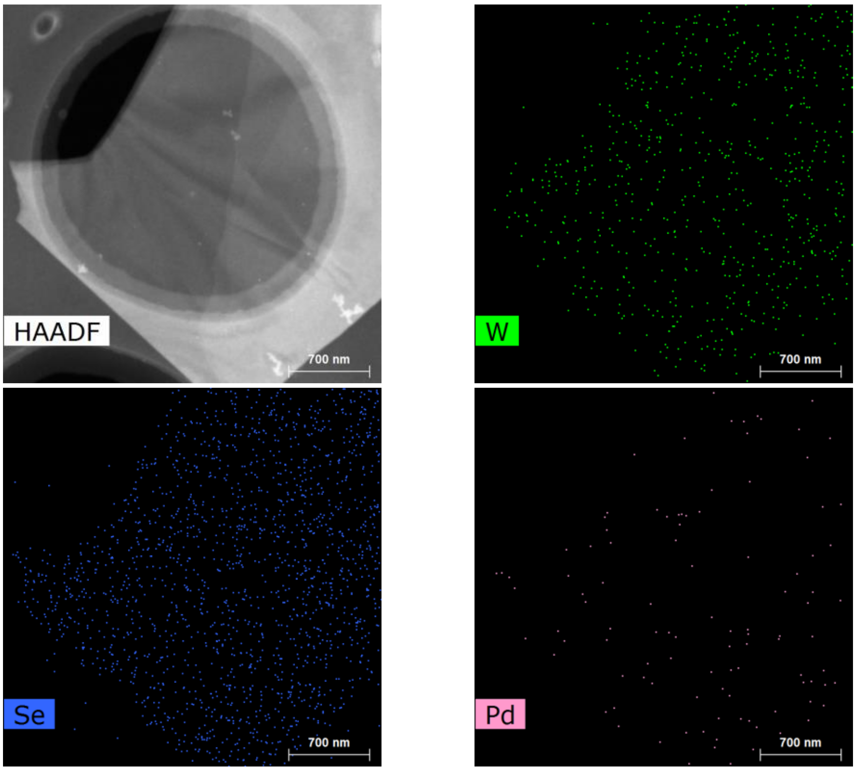

Supplementary Figure 70. STEM-EDS elemental mappings (W, Se and Pd) of Pd SAs-WSe_2.

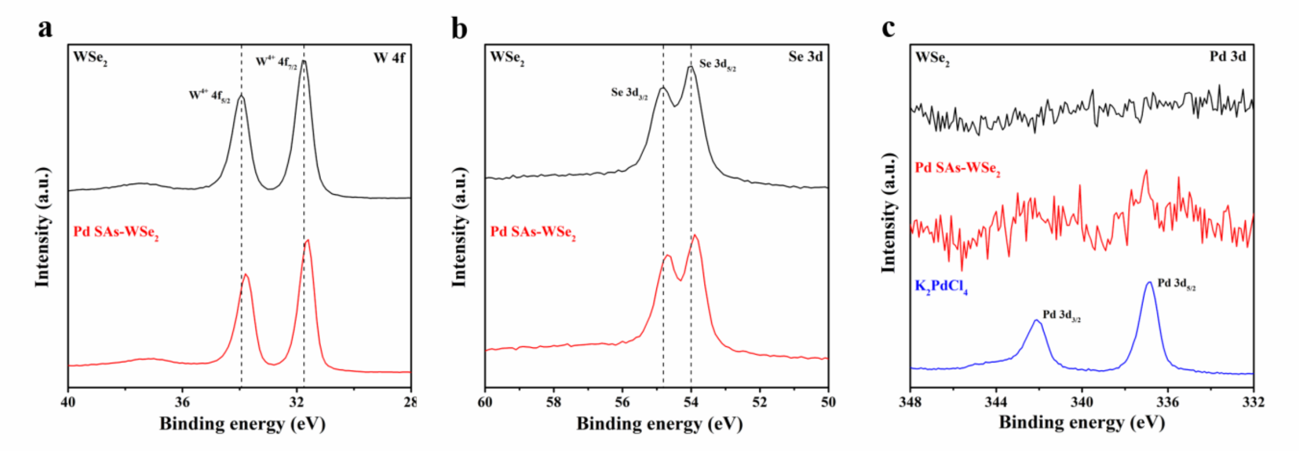

Supplementary Figure 71. XPS spectra showing W 4f (**a**), Se 3d (**b**) and Pd 3d (**c**) core level peak regions for bulk WSe_2 (black) and Pd SAs-WSe_2 (red). K_2PdCl_4 was used as the Pd^{2+} reference.

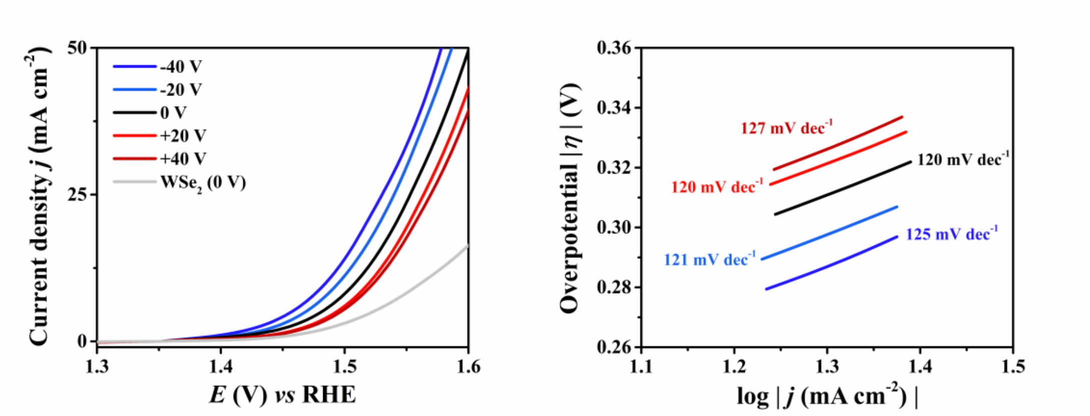

Supplementary Figure 72. HER polarization curves (left) and Tafel plots (right) of Pd SAs-WSe_2 under OEEF regulation.

OER performance of Co NPs-WSe_2 under OEEF regulation. Electrodeposition of Co nanoparticles on WSe_2 was conducted using a linear sweep voltammetry method. A layer of PMMA film was spun on the silicon wafer as the passivation layer, in which a window designed by e-beam lithography was etched by acetone to expose part of WSe_2. A platinum wire was used as the counter electrode and an Ag/AgCl electrode was used as the reference electrode. Then the electrochemical deposition was carried out from 0 V to −0.6 V (*vs* RHE) in the electrolyte containing 0.1 mmol L^{-1} $CoCl_2$ and 0.1 M KOH. Finally, we got the Co NPs (~80 nm) on WSe_2.

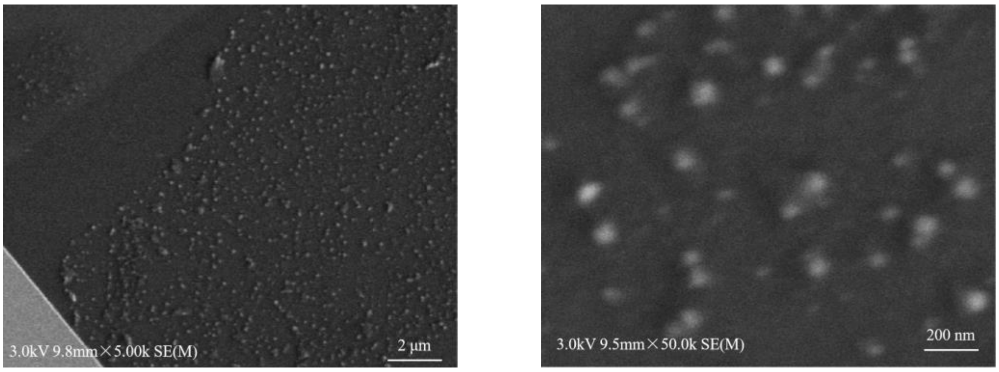

Supplementary Figure 73. Magnified SEM images of Co NPs-WSe_2.

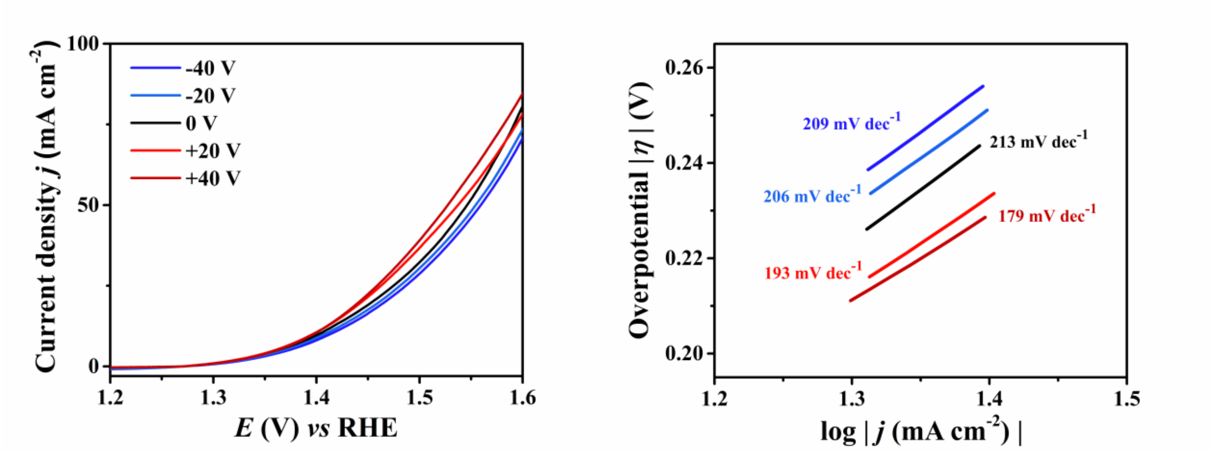

Supplementary Figure 74. HER polarization curves (left) and Tafel plots (right) of Co NPs-WSe_2 under OEEF regulation.

OER performance of 10 nm-thickness RuO_2 film under OEEF regulation. The silicon wafer with prepatterned Au electrodes was spun with a layer of PMMA, patterned by e-beam lithography, and deposited with RuO_2 film (10 nm) through physical evaporation, successively. After annealing at 150 °C in the air for 2 h and lifting off the layer of PMMA with acetone, the 10 nm-thickness RuO_2 film (80 μm × 10 μm) was prepared. A new PMMA film was then spun on the silicon wafer as the passivation layer, in which a window was etched to expose part of RuO_2 film with fixed area (30 μm × 10 μm). Above all, the individual RuO_2 microelectrode was fabricated for OER test.

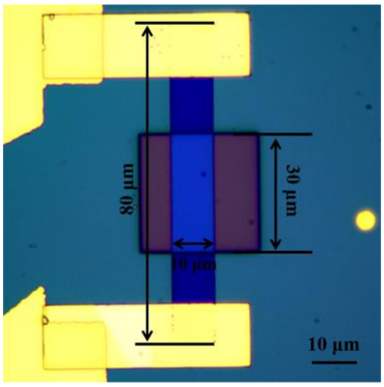

Supplementary Figure 75. Optical image of RuO_2 film.

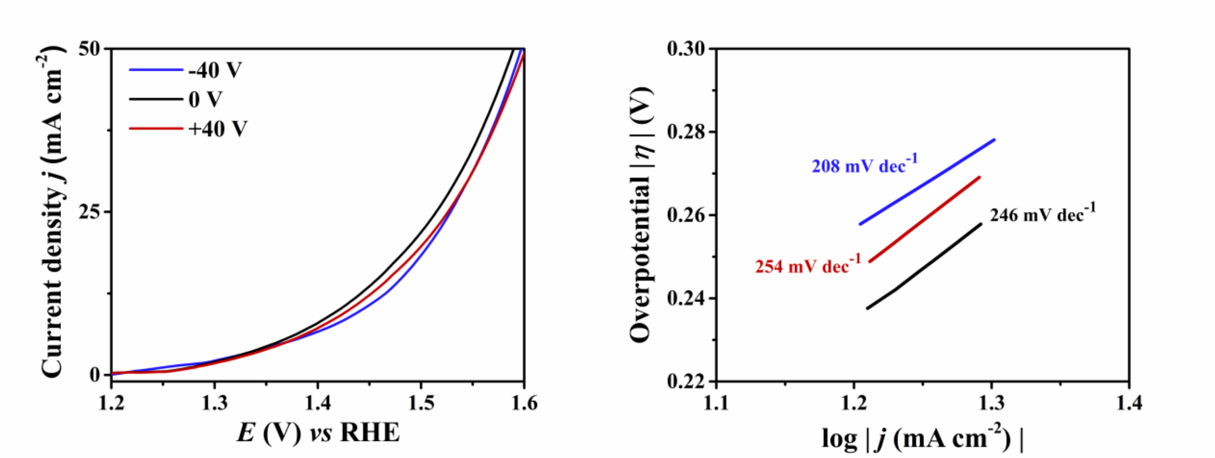

Supplementary Figure 76. OER polarization curves (left) and Tafel plots (right) of RuO_2 microelectrode under OEEF regulation.

Electrocatalytic performance comparison and stability test

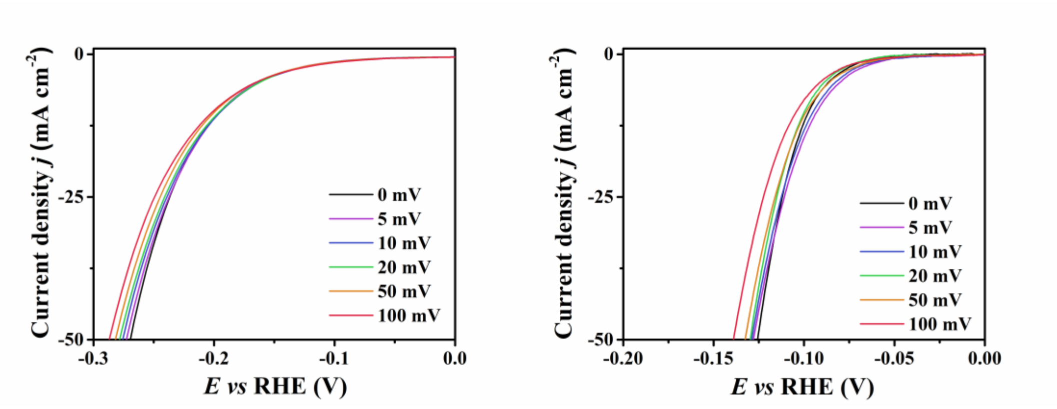

Supplementary Figure 77. HER polarization curves of MoS_2 (left) and Pt SAs-MoS_2 (right) with different biases (V_{ds}=0, 5, 10, 20, 50 and 100 mV).

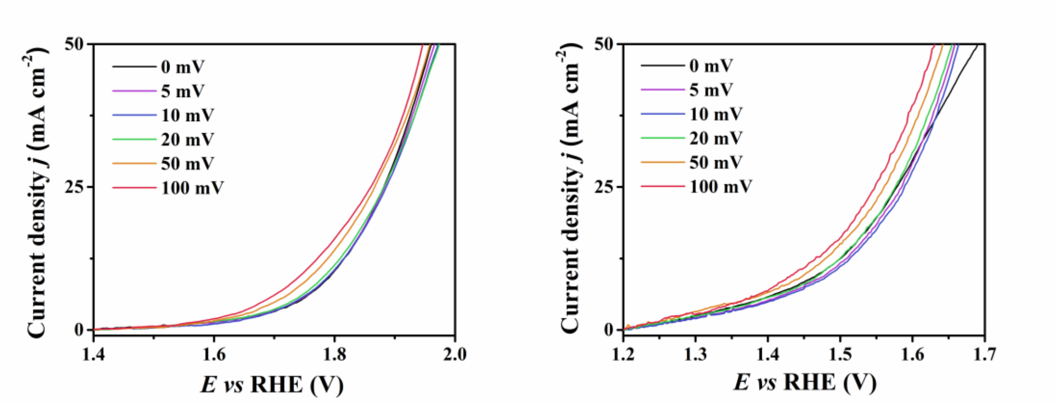

Supplementary Figure 78. OER polarization curves of WSe_2 (left) and Co SAs-WSe_2 (right) with different biases (V_{ds}=0, 5, 10, 20, 50 and 100 mV).

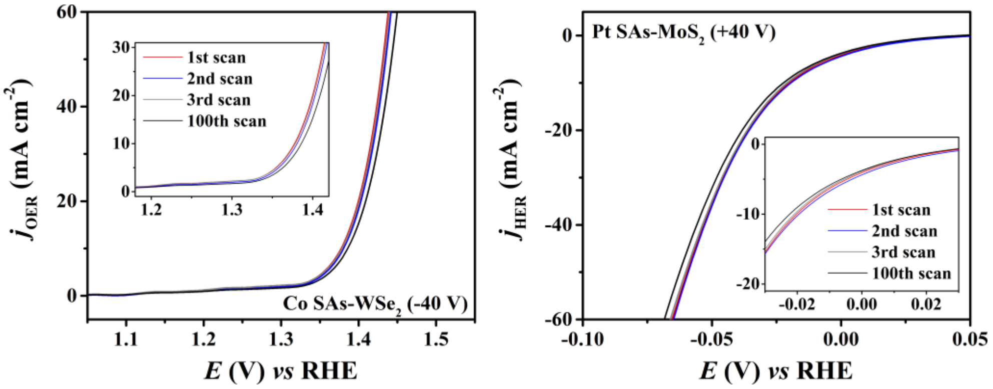

Supplementary Figure 79. The first three scans and the last scan of Pt SAs-MoS_2 for HER (left, V_g= +40 V) and Co SAs-WSe_2 for OER (right, V_g= −40 V) in a 100-cycle LSV test.

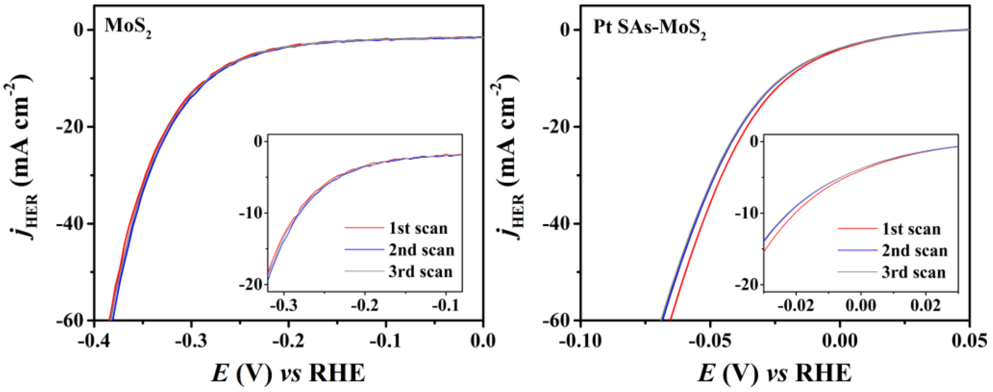

Supplementary Figure 80. The first few scans of the pristine MoS_2 (left) and Pt SAs-MoS_2 (right) in HER test.

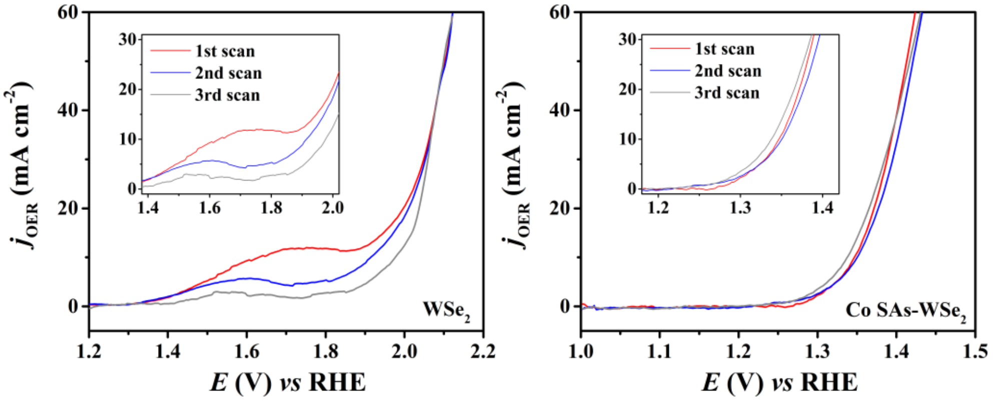

Supplementary Figure 81. The first few scans of the pristine WSe_2 (left) and Co SAs-WSe_2 (right) in OER test.

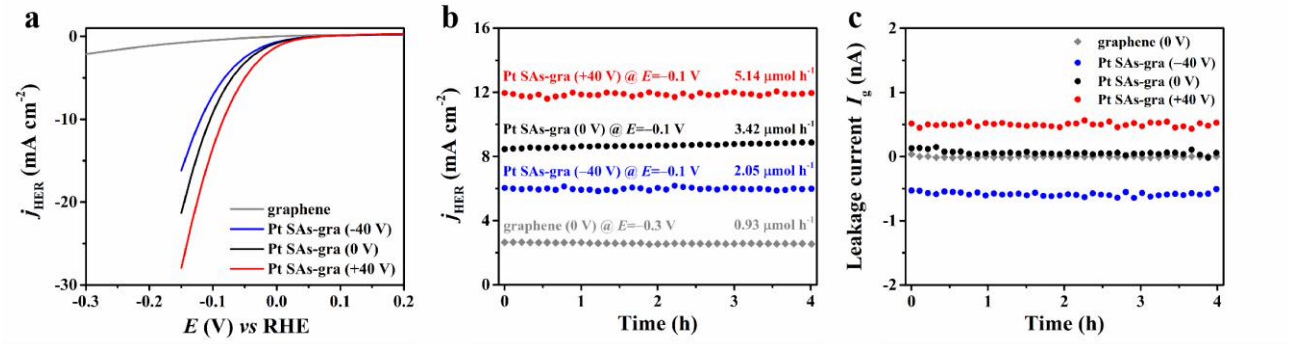

Supplementary Figure 82. HER polarization curves (**a**), chronoamperometric curves (**b**) and corresponding leakage current (**c**) in a 4-h potentiostatic electrolysis test (right) of centimeter-size graphene and Pt SAs-graphene samples under OEEF regulation. The formation rates of H_2 product determined by gas chromatograph analysis are added along each curve in **b**.

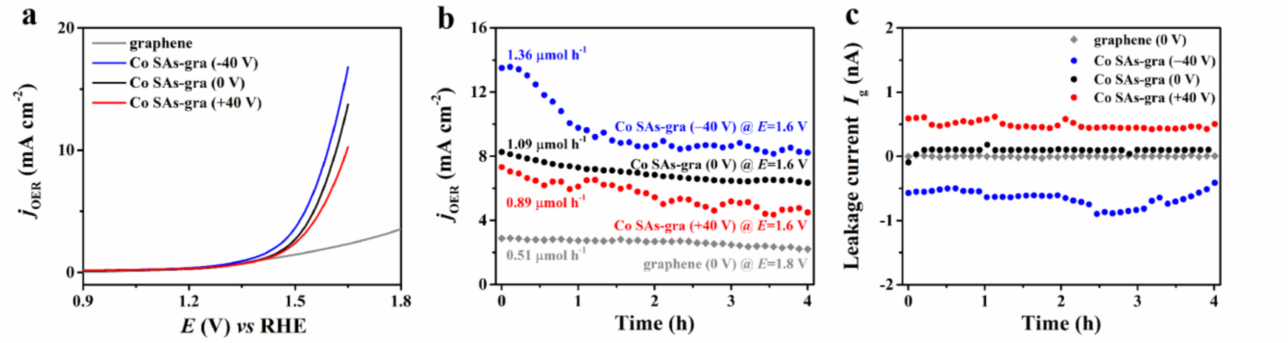

Supplementary Figure 83. OER polarization curves (**a**), chronoamperometric curves (**b**) and corresponding leakage current (**c**) in a 4-h potentiostatic electrolysis test (right) of centimeter-size graphene and Co SAs-graphene samples under OEEF regulation. The formation rates of O_2 product determined by gas chromatograph analysis are added along each curve in **b**.

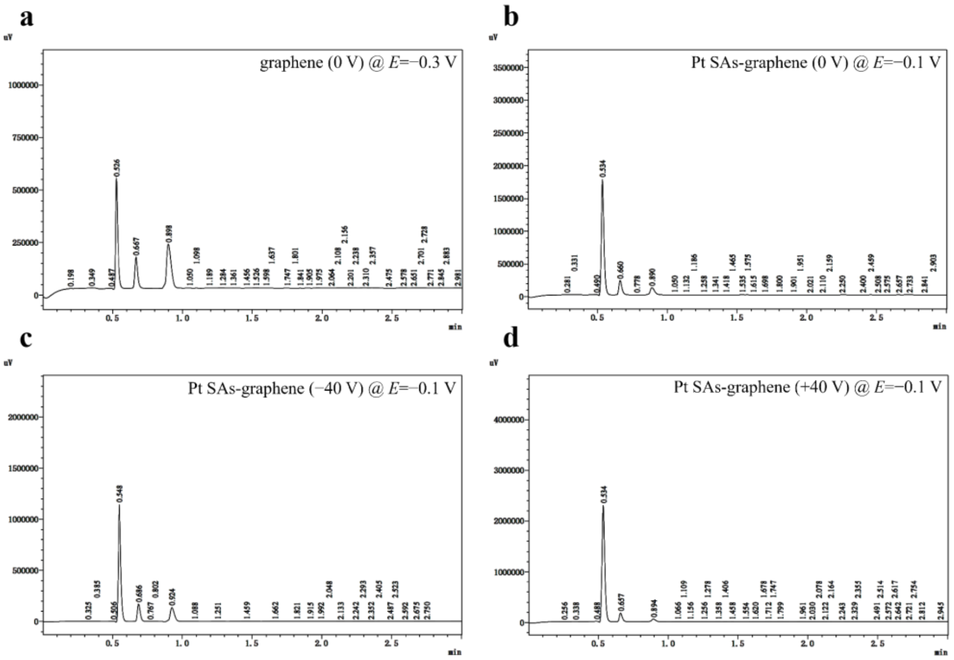

Supplementary Figure 84. Corresponding GC analysis results of gas products from a 4-h potentiostatic electrolysis (shown in **Supplementary Figure 82b)** of graphene (**a**, V_g=0 V) and Pt SAs-graphene (**b** for V_g=0 V, **c** for V_g=−40 V and **d** for V_g=+40 V) for HER under OEEF regulation. The H_2 peak has a retention time of around 0.5 min.

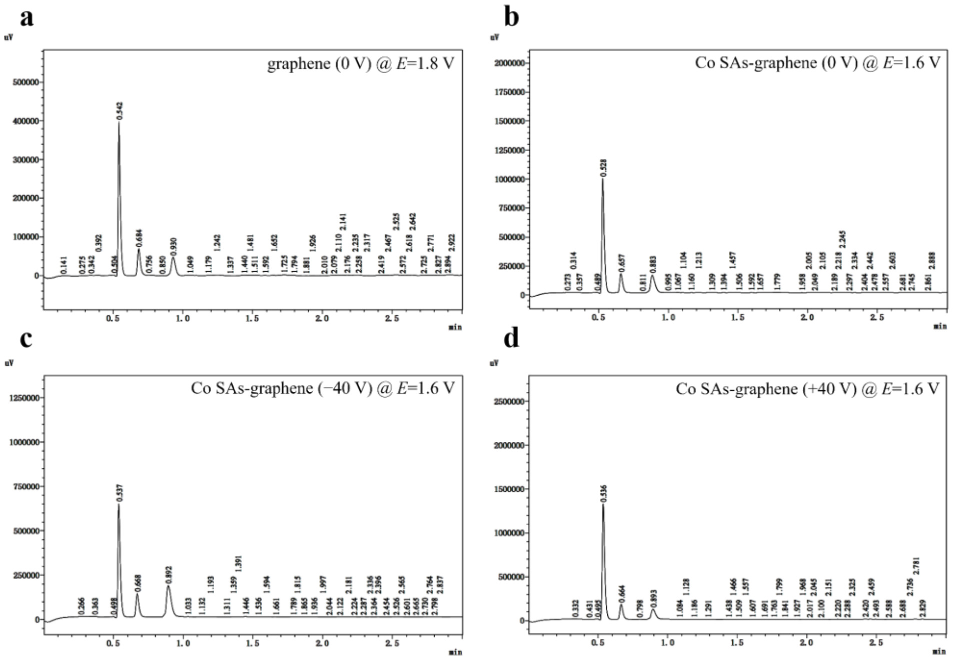

Supplementary Figure 85. Corresponding GC analysis results of gas products from a 4-h potentiostatic electrolysis (shown in **Supplementary Figure 83b)** of graphene (**a**, V_g=0 V) and Co SAs-graphene (**b** for V_g=0 V, **c** for V_g=−40 V and **d** for V_g=+40 V) for OER. The O_2 peak has a retention time of around 0.9 min.

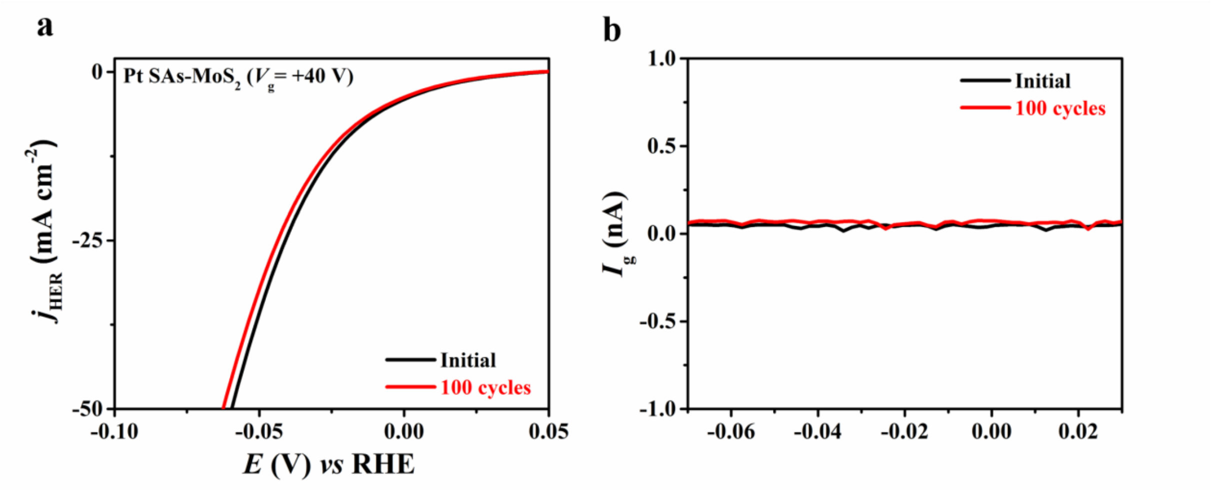

Supplementary Figure 86. HER polarization curves (**a**) of Pt SAs-MoS_2 (V_g=+40 V) before and after 100 CV cycles, and the correspondingly recorded leakage current (**b**) flowing gate electrode.

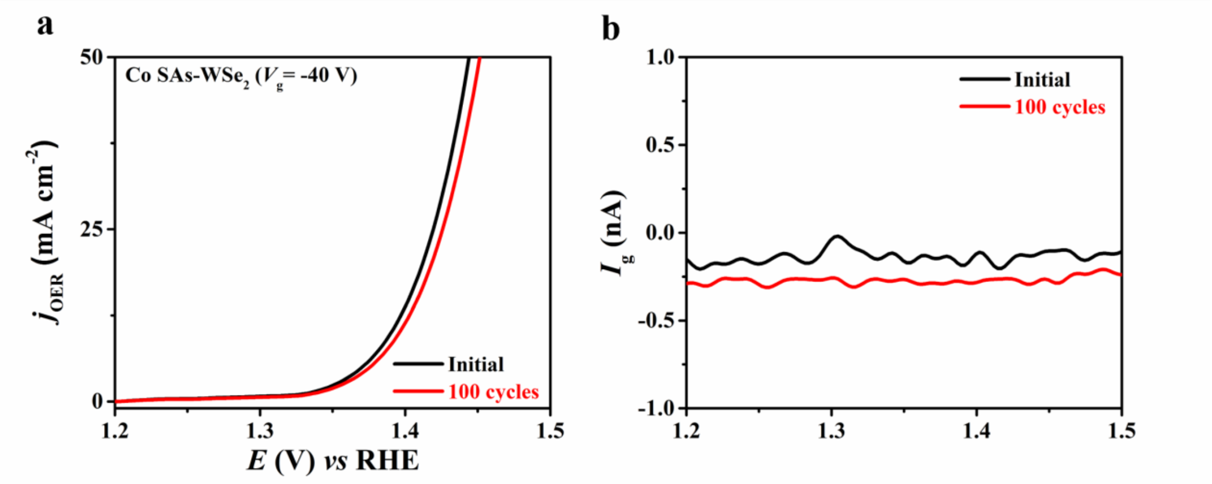

Supplementary Figure 87. OER polarization curves (**a**) of Co SAs-WSe_2 (V_g=−40 V) before and after 100 CV cycles, and the correspondingly recorded leakage current (**b**) flowing gate electrode.

Supplementary Table 5. Mass activity (Pt loading) of Pt SAs-MoS_2 for HER (in the electrolyte of 0.5 M H_2SO_4).

A/mg_{Pt}	−40 V	−20 V	0 V	+20 V	+40 V
η= −0.02 V	106.6	115.5	148.8	201.4	239.1
η= −0.04 V	332.4	386.4	498.2	630.0	758.0
η= −0.05 V	493.8	589.3	761.0	945.3	1171.1

Supplementary Table 6. Mass loading, mass activity, overpotential and Tafel slope of Pt SAs-MoS_2 (V_g = +40 V) for HER, in comparison to several representative results from recent published works.

electrocatalyst	mass loading (wt%)	mass activity @ η= −0.05 V (A/mg_{metal})	overpotential @ 10 mA cm^{-2} (mV)	Tafel slope (mV dec^{-1})
Pt SAs-MoS_2 (+40 V)	4.84	1171.1	20	51
Pt-SAs/WS_2	4.1	55.5	32	28
Pt SAs/DG	2.1	26.2	23	25
Pt-SAs/MoS_2	5.1	17.14	59	31
Pt/NGNs	2.1	10.1	38	29
Pt_1/OLC	0.27	9.4	38	36
$Mo_2TiC_2T_x$-Pt_{SA}	1.2	5.3	30	30
Pt_1/NMHCS	1.59	2.07	40	56

Supplementary Table 7. Mass activity (Co loading) of Co SAs-WSe_2 for OER (in the electrolyte of 0.1 M KOH).

A/mg_{Co}	−40 V	−20 V	0 V	+20 V	+40 V
η=0.14 V	2514.5	1109.5	636.1	423.3	242.9
η=0.15 V	3684.1	1617.9	929.2	618.6	338.1
η=0.20 V	19024.2	8287.4	4763.6	3175.7	1670.5

Supplementary Table 8. Mass loading, mass activity, overpotential and Tafel slope of Co SAs-WSe_2 (V_g = −40 V) for OER, in comparison to several representative results from recent published works.

electrocatalyst	mass loading (wt%)	mass activity (A/mg_{metal}) @ η= 0.2 V	overpotential (mV) @ 10 mA cm^{-2}	Tafel slope (mV dec^{-1})
Co SAs-WSe_2 (−40 V)	0.78	19024.2	139	64
Ru SAs/AC-FeCoNi	0.67	6.2	205	40
Ir_{SA}/Ni_2P	3.6	3.84	149	90.1
Ru_1/D-NiFe LDH	1.2	0.87	189	31
$Ir_{18\ wt\ \%}$-NiO	18	N.A.	215	38
Ni-O-G SACs	3.1	N.A.	224	42

Supplementary Table 9. Overpotential and Tafel slope of Pt SAs-MoS_2 and other reported electrocatalysts for HER.

electrocatalyst	overpotential @ 10 mA cm^{-2} (mV)	Tafel slope (mV dec^{-1})	electrolyte	reference
mechanically exfoliated MoS_2	114 (V_g = +40 V)	114	0.5 M H_2SO_4	this work
CVD-grown monolayer MoS_2	151 (V_g = +40 V)	158	0.5 M H_2SO_4	this work
**Pt SAs-MoS_2**	**20 (V_g = +40 V)**	**51**	**0.5 M H_2SO_4**	**this work**
Pt NPs-MoS_2	46	29	0.5 M H_2SO_4	this work
Co SAs-MoS_2	117 (V_g = +40 V)	72	0.5 M H_2SO_4	this work
mechanically exfoliated WSe_2	268 (V_g = −40 V)	183	0.5 M H_2SO_4	this work
Pt SAs-WSe_2	88 (V_g = +40 V)	109	0.5 M H_2SO_4	this work
mechanically exfoliated graphene	287	159	0.5 M H_2SO_4	this work
Pt SAs-graphene	63 (V_g = +40 V)	108	0.5 M H_2SO_4	this work

Pd SAs-MoS_2	69 (V_g = +40 V)	90	0.5 M H_2SO_4	this work
30 nm-thickness Pt film	45	52	0.5 M H_2SO_4	this work
mechanically exfoliated MoS_2	74 (V_g = +3 V)	99	0.5 M H_2SO_4	Zhang WC, et al. *Small.* 2019, 15, 1900964
CVD-grown MoS_2	176 ± 27 (V_g = +100 V)	100 ± 6	0.5 M H_2SO_4	Wang Y, et al. *Nano Lett.* 2019, 19, 6118
1% Pd-MoS_2	78	80	0.5 M H_2SO_4	Luo ZY, et al. *Nat. Commun.* 2018, 9, 2120
Co-$^{S}MoS_2$	220	92	0.5 M H_2SO_4	Lau THM, et al. *Chem. Sci.* 2018, 9, 4769
$3Co_{Mo}-V_S$	75	57	0.5 M H_2SO_4	Zhou Y, et al. *Nat. Commun.* 2020, 11, 2253
SA Co-D 1T MoS_2	42	32	0.5 M H_2SO_4	Qi K, et al. *Nat. Commun.* 2019, 10, 5231
$Mo_2TiC_2T_x$-Pt_{SA}	30	30	0.5 M H_2SO_4	Zhang JQ, et al. *Nat. Catal.* 2018, 1, 985
Pt-MoS_2	140	96	0.1 M H_2SO_4	Deng J, et al. *Energy Environ. Sci.* 2015, 8, 1594
Pt_1/OLC	38	36	0.5 M H_2SO_4	Liu DB, et al. *Nat. Energy* 2019, 4, 512
Pt SAs/DG	23	25	0.5 M H_2SO_4	Qu YT, et al. *J. Am. Chem. Soc.* 2019, 141, 11, 4505

Pt-GDY2	40	38	0.5 M H_2SO_4	Yin XP, *Angew. Chem. Int. Ed.* 2018, 57, 9382
Pt1/MC	32	25	0.5 M H_2SO_4	Wei HH, et al. *Nat. Commun.* 2017, 8, 1490
Pt/NGN	38	29	0.5 M H_2SO_4	Cheng NC, et al. *Nat. Commun.* 2016, 7, 13838
Pt-GT-1	18 / 66	24	0.5 M H_2SO_4 / 0.1 M $HClO_4$	Tiwari JN, et al. *Nat. Energy* 2018, 3, 773
Pt-TiS_2	70	40.6	0.5 M H_2SO_4	Zeng ZY, et al. *Energy Environ. Sci.* 2014, 7, 797
O_2-based / N_2^*-based ALD Pt NPs	40	N.A.	0.5 M H_2SO_4	Dendooven J, et al. *Nat. Commun.* 2017, 8, 1074
Pt-MoS_2	50	40	0.5 M H_2SO_4	Huang X, et al. *Nat. Commun.* 2013, 4, 1444
Pt-SAs/MoS_2	59	31	0.5 M H_2SO_4	Shi Y, et al. *Nat. Commun.* 2020, 11, 4558
Pt_1/NMHCS	40	56	0.5 M H_2SO_4	Kuang PY, et al. Adv. Mater. 2021, 2008599
Pt-SAs/WS_2	32	28	0.5 M H_2SO_4	Shi Y, et al. *Nat. Commun.* 2021, 12, 3021
Pt/MoS_2/CFs	30	53.6	0.5 M H_2SO_4	Hou DM, et al. *Electrochimica Acta* 2015, 166, 26

Supplementary Table 10. Overpotential and Tafel slope of Co $SAs-WSe_2$ and other reported electrocatalysts for OER.

electrocatalyst	overpotential @ 10 mA cm^{-2} (mV)	Tafel slope (mV dec^{-1})	electrolyte	reference
mechanically exfoliated WSe_2	400 (V_g = −40 V)	224	0.1 M KOH	this work
**Co $SAs-WSe_2$**	**139 (V_g = −40 V)**	**64**	**0.1 M KOH**	**this work**
Co $NPs-WSe_2$	168	194	0.1 M KOH	this work
Pt $SAs-WSe_2$	270 (V_g= −40 V)	247	0.1 M KOH	this work
mechanically exfoliated MoS_2	590 (V_g = +40 V)	116	0.1 M KOH	this work
Co $SAs-MoS_2$	220 (V_g = −40 V)	153	0.1 M KOH	this work
mechanically exfoliated graphene	449	148	0.1 M KOH	this work
Co SAs-graphene	248 (V_g= −40 V)	89	0.1 M KOH	this work
Pd $SAs-WSe_2$	255 (V_g= −40 V)	125	0.1 M KOH	this work

10nm-thickness RuO_2 film	212	208	0.1M KOH	this work
mechanically exfoliated WSe_2	230	101	0.1 M KOH	He YM, et al. *Nat. Mater.* 2019, 18, 1098
WSe_2: CdS NHDs	290	49	0.1 M KOH	Karfa P, et al. *J. Mater. Chem. A.* 2017, 5, 1495–1508
Co-MoS_2/BCCF-21	260	85	1.0 M KOH	Xiong QZ, et al. *Adv. Mater.* 2018, 30, 1801450
A-Ni@DG	270	47	1.0 M KOH	Zhang LZ, et al. *Chem.* 2018, 4, 285−297
PHNCMs	235	45.7	1.0 M KOH	Li HY, et al. *Nat. Commun.* 2017, 8, 15377
Ni-NHGF / Co-NHGF	331 / 402	63 / 80	1.0 M KOH	Fei HL, et al. *Nat. Catal.* 2018, 1, 63−72
G-FeCoW	191	37	1.0 M KOH	Zhang B, et al. *Science* 2016, 352, 333-337
w-$Ni(OH)_2$	237	33	1.0 M KOH	Yan JQ, et al. *Nat. Commun.* 2019, 10. 2149
meso-Fe-MoS_2/$CoMo_2S_4$	290	65	1.0 M KOH	Guo YN, et al. *ACS Nano* 2020
$CoSe_2$–D_{Fe}–V_{Co}	320	53.5	1.0 M NaOH	Dou YH, et al. *Nat. Commun.* 2020, 11, 1664
Ru-RuO_2/D-CTFs-300	190	41	1.0 M KOH	Gao X, et al. *J. Phys. Chem.* 2020, 50, 135-142

$Ir_{18\ wt\ \%}$-NiO	215	38	1.0 M KOH	Wang Q, et al. *J. Am. Chem. Soc.* 2020, 142, 16, 7425
Ni-O-G SACs	224	42	1.0 M KOH	Li YG, et al. *Adv. Sci.* 2020, 1903089
D-U-$Co(OH)_2$	223	131	1.0 M KOH	Zhang BW, et al. *ACS Energy Lett.* 2019, 4, 1, 328-336
Ru SAs/AC-FeCoNi	205	40	1.0 M KOH	Hu YD, et al. *Adv. Energy Mater.* 2020, 2002816
Ru_1/D-NiFe LDH	189	31	1.0 M KOH	Zhai PL, et al. *Nat. Commun.* 2021, 12, 4587
Ru-MoS_2-Mo_2C/TiN	280	202	1.0 M KOH	V. H. Hoa, et al. *Nano Energy* 2021, 106277
Ir_{SA}-Ni_2P	149	90.1	1.0 M KOH	Wang Q, et al. *J. Am. Chem. Soc.* 2021, 143, 34, 13605

Computational details

In the systems of MoS_2 and Pt SAs-MoS_2 for HER

Monolayer model of MoS_2 was cleaved from 2H-MoS_2 unit cell (***a***=***b***=3.18 Å). A vacuum layer of 20 Å is set in the normal direction to the plane to minimize image interactions. The density functional theory (DFT) simulations were done on periodically repeated 4 × 4 × 1 supercells with 3.1% sulfur vacancies (1/32), which are usually regarded as the main active sites in the basal plane of 2H-MoS_2 for HER[1-3]. To investigate the effect of externally applied electric fields on Pt SAs-MoS_2 proposed in this work, three possible binding sites (*X-sub*, *M-top* and *h-top*, **Supplementary Figure 88**) were considered into DFT simulations, in which the models of *X-sub* and *M-top* have lower formation energies ($E_{formation}$) than ***h-top***. Confirmed by XAS results (**Supplementary Table 3**) and STEM observations (**Supplementary Figure 2**), the *M-top* site binding to S atom was constructed as the optimal model and therefore we introduced electric field gradient (E_{field}, from −0.4 V/Å to +0.4 V/Å) into (*M-top*)-Pt SAs-MoS_2 for subsequent theoretical analysis (including PDOS and charge analysis through introducing different E_{field}).

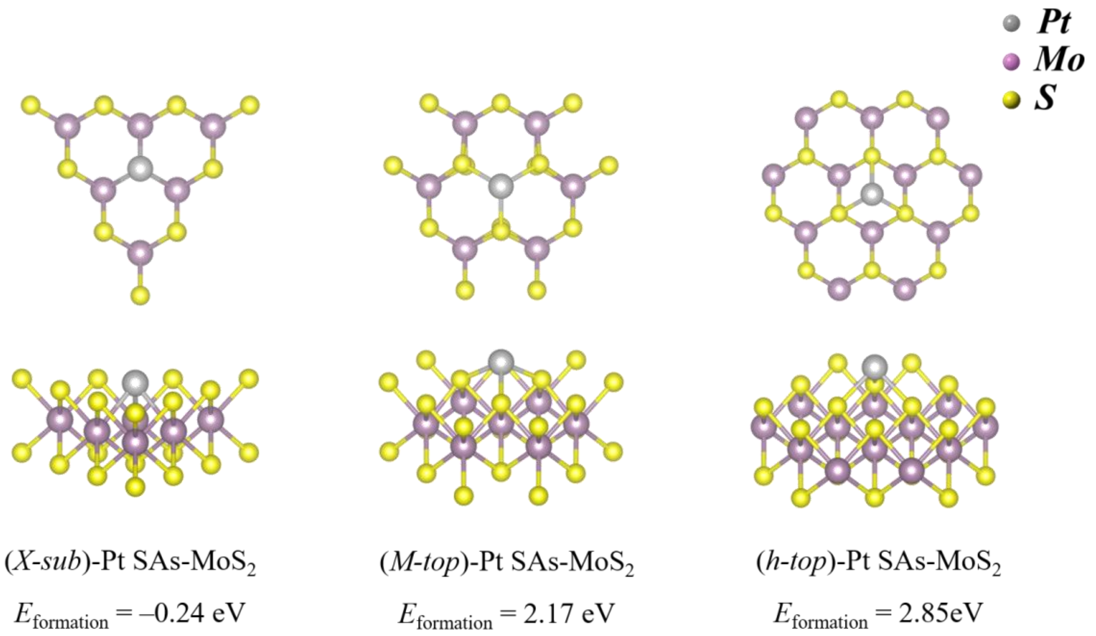

Supplementary Figure 88. Three models of Pt single site binding on 2H-MoS_2 and formation energies. ***X-sub***: metal atom stays at S-vacancy site. ***M-top***: metal atom stays at the top of Mo atom in pristine MoS_2. ***h-top***: metal atom stays at the top of triangular prism in pristine MoS_2.

Supplementary Table 11. Bond length of Pt atom binds to MoS_2 in different DFT models.

model	*X-sub*	*M-top*	*h-top*
binding form	Pt-Mo	Pt-S	Pt-S
bond length	2.632	2.291	2.343

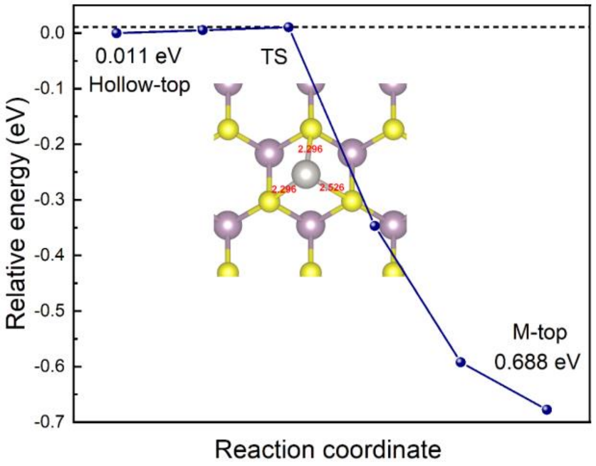

Supplementary Figure 89. Calculated energy barrier of migrating Pt atom from *h-top* to *M-top*. The energy barrier of migrating Pt atom from *h-top* to *M-top* is about 0.011 eV, indicating *h-top* site could be easily transferred to *M-top* site.

The Gibbs free energy of each intermediate is given by $G = E + E_{\mathrm{ZPE}} - \mathrm{T}S + \Delta U^{0\rightarrow \mathrm{T}}$, where E is the total energy of adsorption model, and T is equal to 298.15 K. E_{ZPE}, S and $\Delta U^{0\rightarrow \mathrm{T}}$ are zero-point energy, entropy, and internal energy at temperature T of intermediate, respectively. The values of S and $\Delta U^{0\rightarrow \mathrm{T}}$ could be calculated from equations (S1) and (S2), respectively.

$$S = R\sum_{i}\left\{\frac{hv_i}{k_{\mathrm{B}}\mathrm{T}}\frac{e^{-\frac{hv_i}{k_{\mathrm{B}}\mathrm{T}}}}{1-e^{-\frac{hv_i}{k_{\mathrm{B}}\mathrm{T}}}} - ln\left[1-e^{-\frac{hv_i}{k_{\mathrm{B}}\mathrm{T}}}\right]\right\} \tag{S1}$$

$$\Delta U^{0\rightarrow T} = R\sum_{i}\left(\frac{hv_i}{k_{\mathrm{B}}}\right)\left(\frac{1}{2}+\frac{e^{-\frac{hv_i}{k_{\mathrm{B}}\mathrm{T}}}}{1-e^{-\frac{hv_i}{k_{\mathrm{B}}\mathrm{T}}}}\right) \tag{S2}$$

where R is molar gas constant, k_{B} is Boltzmann constant, h is Plank constant, and v_i is frequency for the i-th vibration mode. All these thermodynamic correction terms could be calculated using VASPKIT[4]. The frequency below 50 cm^{-1} is set to 50 cm^{-1}. The computational data for E_{ZPE}, S and $\Delta U^{0\rightarrow \mathrm{T}}$ are listed in **Supplementary Table 12** (for hydrogen intermediates) and **Supplementary Table 17** (for oxygen intermediates), respectively.

Supplementary Table 12. Detailed thermodynamic corrections to the Gibbs free energy (eV) of hydrogen intermediates at T=298.15 K.[a]

Models	Intermediates	E_{ZPE}	TS	$\Delta U^{0\rightarrow T}$
2H-MoS_2	*H	0.157	0.010	0.008
(*M-top*)-Pt SAs-MoS_2	*H	0.162	0.046	0.027

[a] The calculated results under zero electric field were used uniformly, because electric filed has negligible influence on the thermodynamic corrections.

For HER, one water molecule was putted on top of the adsorbed *H in the model of (*M-top*)-Pt SAs-MoS_2 to investigate the OEEF-induced effect from solvents at the interface.

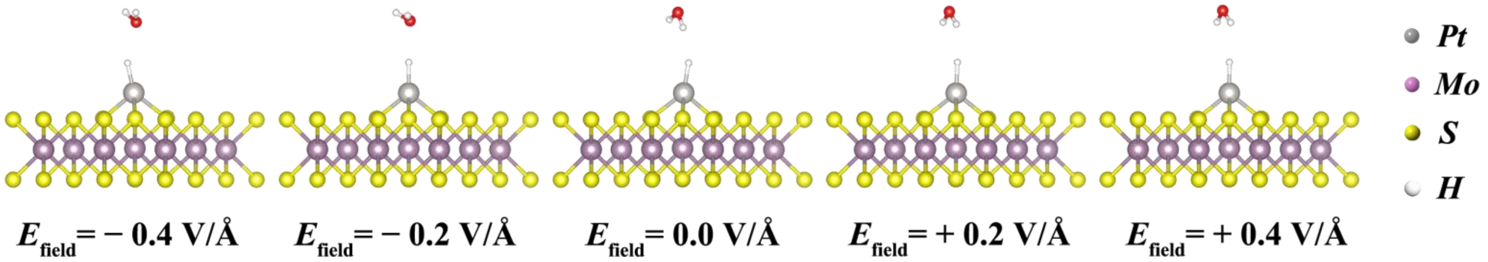

Supplementary Figure 90. The explicit solvation models of (*M-top*)-Pt SAs-MoS_2 (*H adsorbed), with E_{field} applied from −0.4 V/Å to +0.4 V/Å.

Supplementary Table 13. Hydrogen adsorption free energies (ΔG_H, U=0 V) of 2H-MoS_2 (Mo atom) and (*M-top*)-Pt SAs-MoS_2 (Pt atom, gas-phase and explicit solvation models), with E_{field} applied from −0.4 V/Å to +0.4 V/Å.

ΔG_H (eV)		−0.4 V/Å	−0.2 V/Å	0.0 V/Å	+0.2 V/Å	+0.4 V/Å
2H-MoS_2		0.047	0.049	0.051	0.053	0.055
(*M-top*)-Pt SAs-MoS_2	gas-phase	0.098	0.040	−0.025	−0.092	−0.165
	solvation	0.373	0.360	0.250	0.171	0.078

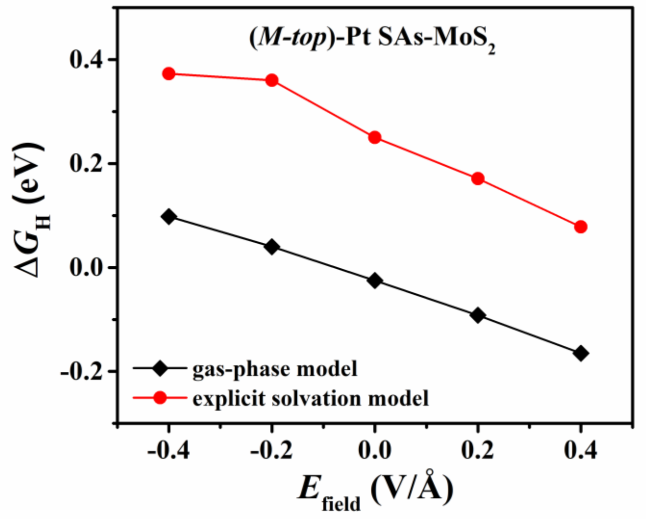

Supplementary Figure 91. Hydrogen adsorption energy (ΔG_H) in the gas-phase and explicit solvation models of (*M-top*)-Pt SAs-MoS_2.

The PDOS projected onto the H 1s and the Pt $5d_{z^2}$ orbitals in (*M-top*)-Pt SAs-MoS_2 was shown in **Supplementary Figure 92**, and the band centers[5] of H 1s-Pt $5d_{z^2}$ bonding states and antibonding states were calculated with following equations (S3) and (S4). Then the energy gap ($\Delta\varepsilon = \varepsilon_b - \varepsilon_{ab}$) between the centers of H 1s-Pt $5d_{z^2}$ bonding states (ε_b) and antibonding states center (ε_{ab}) was determined and correlated with the OEEF-modulated hydrogen adsorption free energy (ΔG_H). Similar procedures were implemented for the model of (*M-top*)-Co SAs-WSe_2 with O* adsorbed (shown in **Supplementary Figure 102**).

$$\varepsilon_b = \frac{\int_b n_b(\varepsilon)\varepsilon d\varepsilon}{\int_b n_b(\varepsilon) d\varepsilon} \tag{S3}$$

$$\varepsilon_{ab} = \frac{\int_{ab} n_{ab}(\varepsilon)\varepsilon d\varepsilon}{\int_{ab} n_{ab}(\varepsilon) d\varepsilon} \tag{S4}$$

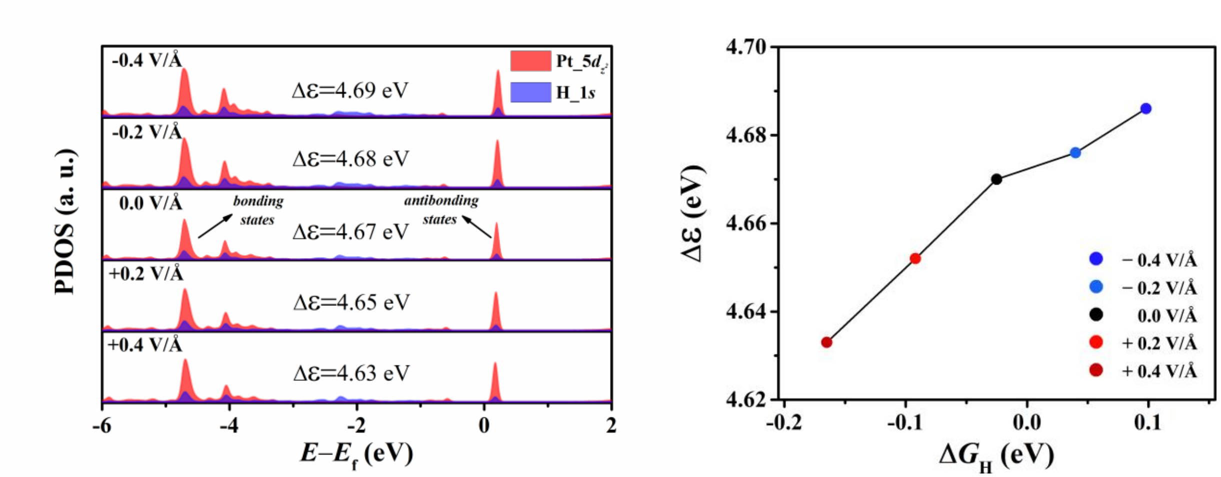

Supplementary Figure 92. PDOS (left) of H 1s orbital (blue) and Pt 5d$_{z^2}$ orbital (red) in (*M-top*)-Pt SAs-MoS_2 at different OEEFs, showing the energy gap (Δε) between the centers of H 1s-Pt 5d$_{z^2}$ bonding states and antibonding states and its correlation (right) with hydrogen adsorption free energy (ΔG_H).

Bader charge analysis[6] (**Supplementary Tables 14**, **15**) was performed on (*M-top*)-Pt SAs-MoS_2 under OEEF regulation, to reveal the electric-field modulation on Pt-support interaction. Notably, although ΔG_H and Q of the anion vacancy MoS_2 are also associated with E_{field}, the change of which is actually slight (consistent with Ling's work[7]). More importantly, compared to Q_H of *H adsorbed on 2H-MoS_2 (Mo atoms), the charge density of *H adsorbed on Pt SAs-MoS_2 (Pt atom) experiences a significant alteration under OEEF regulation, which contributes to the "onsite electrostatic polarization" mechanism we proposed here (**Fig. 3c** and **Supplementary Figures 93**, **94**).

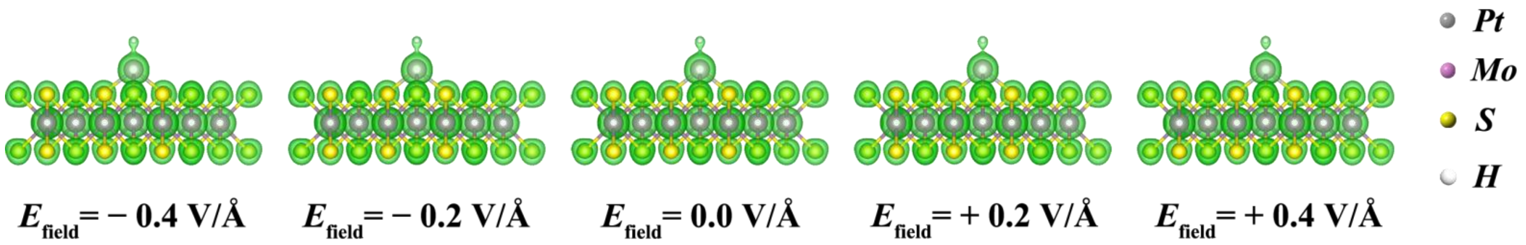

Supplementary Figure 93. Charge densities (green) of (*M-top*)-Pt SAs-MoS_2 at different OEEFs (E_{field} from −0.4 V/Å to +0.4 V/Å), where *H is adsorbed on Pt atom. The interface value is 0.15 $e^-/Bohr^3$.

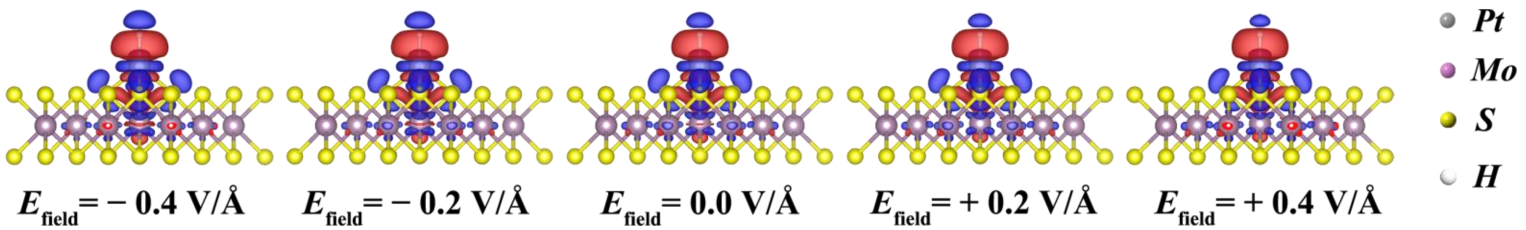

Supplementary Figure 94. Deformation charge densities of (*M-top*)-Pt SAs-MoS_2 at different OEEFs (E_{field} from −0.4 V/Å to +0.4 V/Å), where *H is adsorbed on Pt atom. The interface value is 0.001 $e^-/Bohr^3$. Red and blue areas denote electron accumulation and depletion, respectively.

Supplementary Table 14. Bader charge of 2H-MoS_2 (Q_{Mo}), (*M-top*)-Pt SAs-MoS_2 (Q_{Pt}) and the adsorbed *H (Q_H), with E_{field} applied from −0.4 V/Å to +0.4 V/Å. Note that the H (1s), S (3s, 3p), Mo (4p, 5s, 4d) and Pt (6s, 5d) electrons were treated as valance states during all DFT calculations.

model		Bader charge	−0.4 V/Å	−0.2 V/Å	0.0 V/Å	+0.2 V/Å	+0.4 V/Å
2H-MoS_2	without *H adsorbed	Q_{Mo}	13.203	13.204	13.204	13.205	13.205
	with *H adsorbed	Q_{Mo}	12.803	12.804	12.801	12.803	12.801
		Q_H	2.365	2.368	2.372	2.375	2.377
(*M-top*)-Pt SAs-MoS_2	without *H adsorbed	Q_{Pt}	9.718	9.748	9.799	9.811	9.858
	with *H adsorbed	Q_{Pt}	8.770	8.760	8.781	8.772	8.777
		Q_H	**1.944**	**1.982**	**2.008**	**2.044**	**2.080**

Supplementary Table 15. Bader charge of the adsorbed *H (Q_H) in the gas-phase and explicit solvation models of (*M-top*)-Pt SAs-MoS_2, with E_{field} applied from −0.4 V/Å to +0.4 V/Å.

Q_H	−0.4 V/Å	0.0 V/Å	+0.4 V/Å
gas-phase model	1.944	2.008	2.080
explicit solvation model	1.964	1.989	2.070

We further injected electrons related to excess carrier density ($n=CV_g/q$, where C is the capacitance of 275 nm SiO_2 and V_g is the gate voltage) to the model of (*M-top*)-Pt SAs-MoS_2, which is equivalent to V_g from −60 V to +60 V. In order to measure the specific capacitance of 275 nm SiO_2, a gold electrode (1.5 mm×1.5 mm) was deposited on the silicon wafer to constructed an Au/SiO_2/Si structure. By sweeping gate voltage applied on it at different scan rates, the capacitive current density was obtained and the value of C was calculated to be 53.56 nF/cm^2. See more details in **Supplementary Figure 95** and **Supplementary Table 16**.

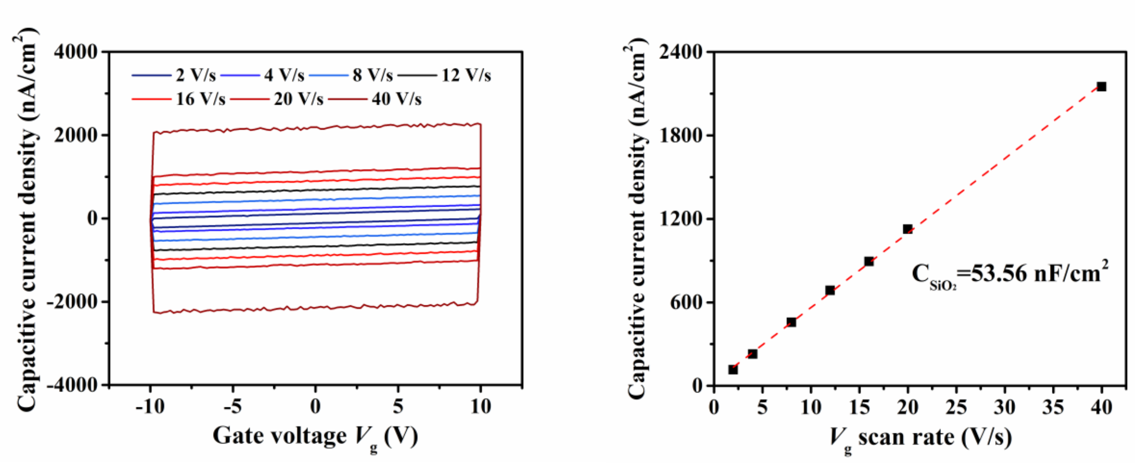

Supplementary Figure 95. Capacitive current density at different V_g applied on an Au/SiO_2/Si structure (left), and the averaged capacitive current density as a function of V_g scan rates (right).

Supplementary Table 16. The electrons injected into the model of (*M-top*)-Pt SAs-MoS_2 at different OEEFs (V_g from −60 V to +60 V).

V_g (V)	n (cm^{-2})	injected electrons (e^-)
−60	-2.009E+13	-0.2792
−40	-1.339E+13	-0.1861
−20	-6.695E+12	-0.0931
0	0.000E+00	0.0000
+20	6.695E+12	0.0931
+40	1.339E+13	0.1861
+60	2.009E+13	0.2792

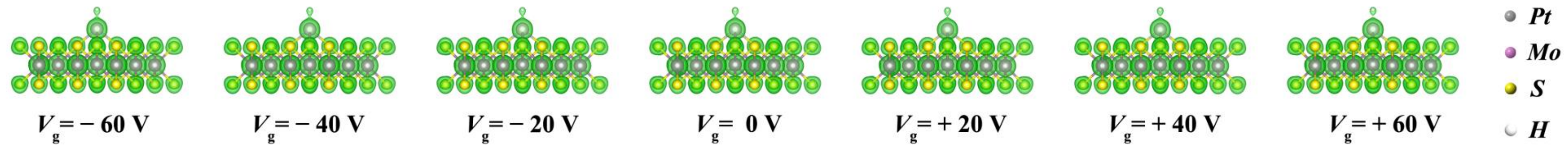

Supplementary Figure 96. Charge densities (green) of (*M-top*)-Pt SAs-MoS_2 at different OEEFs (V_g from −60 V to +60 V), where *H is adsorbed on Pt atom. The interface value is 0.15 e^-/$Bohr^3$.

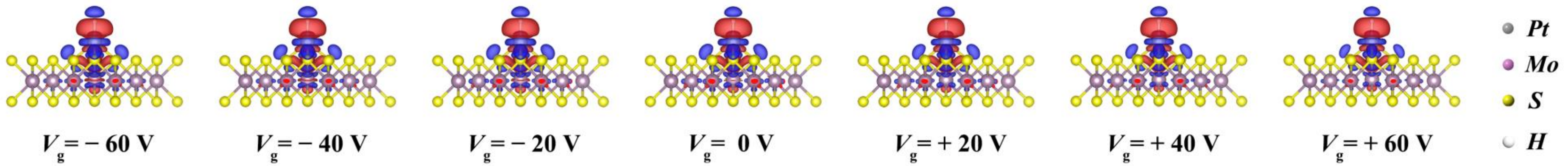

Supplementary Figure 97. Deformation charge densities of (*M-top*)-Pt SAs-MoS_2 at different OEEFs (V_g from −60 V to +60 V), where *H is adsorbed on Pt atom. The interface value is 0.001 e^-/$Bohr^3$. Red and blue areas denote electron accumulation and depletion, respectively.

In the systems of WSe_2 and Co SAs-WSe_2 for OER

Monolayer model of WSe_2 was cleaved from 2H-WSe_2 unit cell (***a***=***b***=3.30 Å). A vacuum layer of 20 Å is set in the normal direction to the plane to minimize image interactions. The density functional theory (DFT) simulations were done on periodically repeated 4 × 4 × 1 supercells. Due to the fact that the Co K-edge XANES could not be well recorded for determining the precise electronic structure of Co SAs-WSe_2 samples, we considered all cases of Co atom binding to 2H-WSe_2 and constructed three possible models (**Supplementary Figure 98**). In line with the STEM observations, the two optimal DFT models of (*M-top*)-Co SAs-WSe_2 and (*X-sub*)-Co SAs-WSe_2 with lower formation energies were applied for subsequent theoretical analysis (including PDOS and charge analysis through introducing different E_{field}).

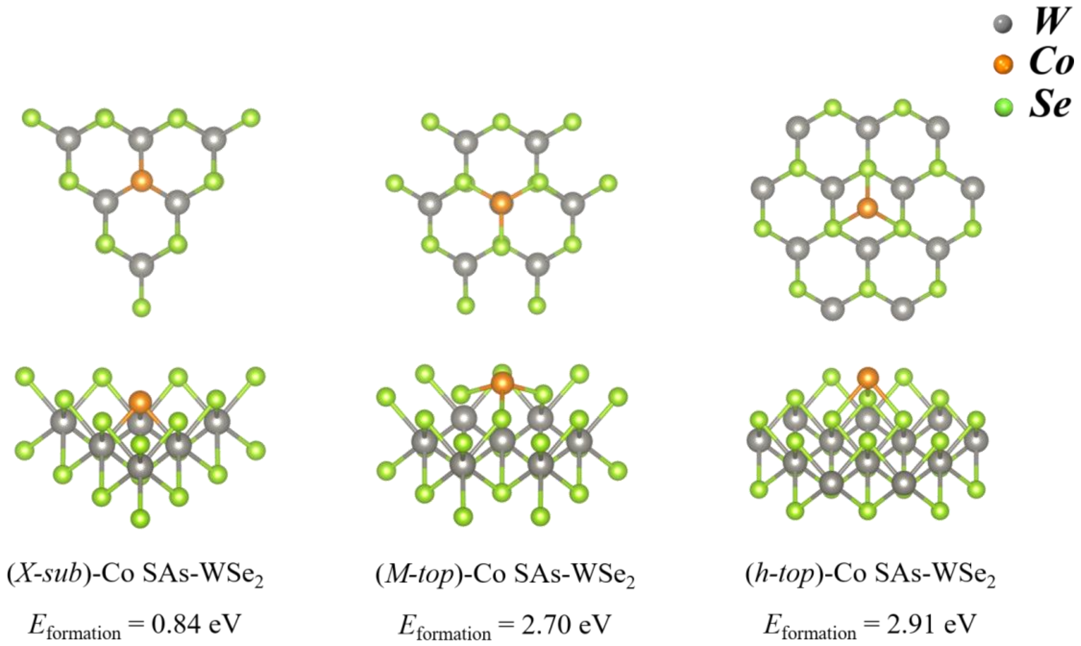

Supplementary Figure 98. Three models of Co single site binding on 2H-WSe_2 and formation energies. ***X-sub***: metal atom stays at Se-vacancy site. ***M-top***: metal atom stays at the top of W atom in pristine WSe_2. ***h-top***: metal atom stays at the top of triangular prism in pristine WSe_2.

Supplementary Table 17. Thermodynamic corrections to Gibbs free energy (eV) of oxygen intermediates at T=298.15 K.[a]

Models	Intermediates	E_{ZPE}	TS	$\Delta U^{0\rightarrow T}$
2H-WSe_2	*OH	0.351	0.054	0.036
	*O	0.090	0.027	0.019
	*OOH	0.351	0.183	0.092
(*M-top*)-Co SAs-WSe_2	*OH	0.341	0.101	0.057
	*O	0.063	0.083	0.040
	*OOH	0.429	0.194	0.097
(*X-sub*)-Co SAs-WSe_2	*OH	0.340	0.103	0.057
	*O	0.054	0.041	0.021
	*OOH	0.437	0.124	0.070

[a] The calculated results under zero electric field were used uniformly, because electric filed has negligible influence on the thermodynamic corrections.

Supplementary Table 18. Adsorption free energies of oxygen intermediates (*OH, *O, and *OOH, U=1.23 V) and free energy differences (ΔG_1, ΔG_2 and ΔG_3) in 2H-WSe_2 (W atom), with E_{field} applied from −0.4 V/Å to +0.4 V/Å.

ΔG (eV)	−0.4 V/Å	−0.2 V/Å	0.0 V/Å	+0.2 V/Å	+0.4 V/Å
ΔG_{OH}	−1.198	−1.178	−1.161	−1.144	−1.134
ΔG_{O}	−4.148	−4.153	−4.157	−4.162	−4.167
ΔG_{OOH}	−2.347	−2.365	−2.399	−2.449	−2.604
$\Delta G_1=\Delta G_{OH}$	−1.198	−1.178	−1.161	−1.144	−1.134
$\Delta G_2=\Delta G_{O}-\Delta G_{OH}$	−2.950	−2.975	−2.996	−3.018	−3.033
$\Delta G_3=\Delta G_{OOH}-\Delta G_{O}$	**1.801**	**1.788**	**1.758**	**1.713**	**1.563**

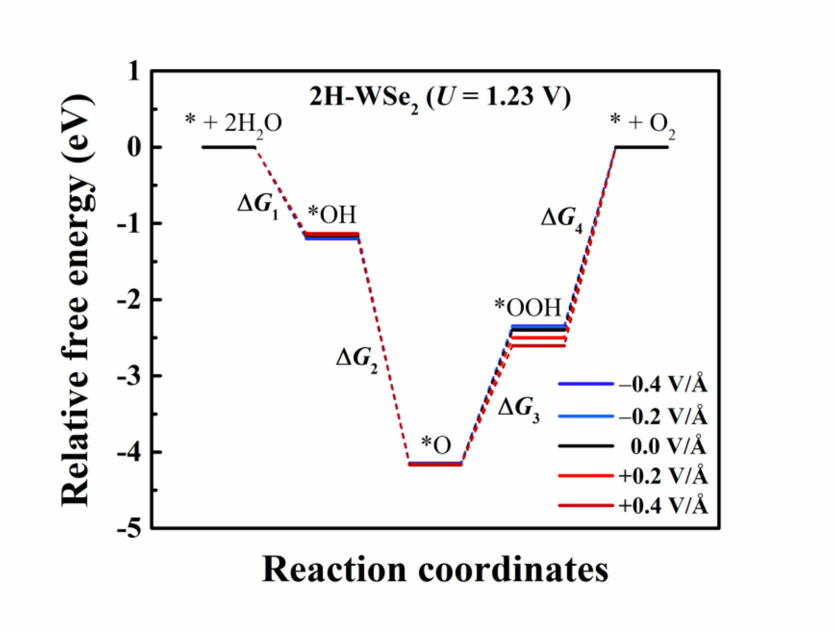

Supplementary Figure 99. Adsorption free energies diagram of 2H-WSe_2 for OER under OEEF regulation, where *OH, *O and *OOH is successively absorbed on the exposed W atom.

Furthermore, two water molecules were placed around the oxygen intermediates (*OH, *O and *OOH) in (*M-top*)-Co SAs-WSe_2 for OER to consider the influence of short-ranged intermolecular hydrogen bonding interactions.

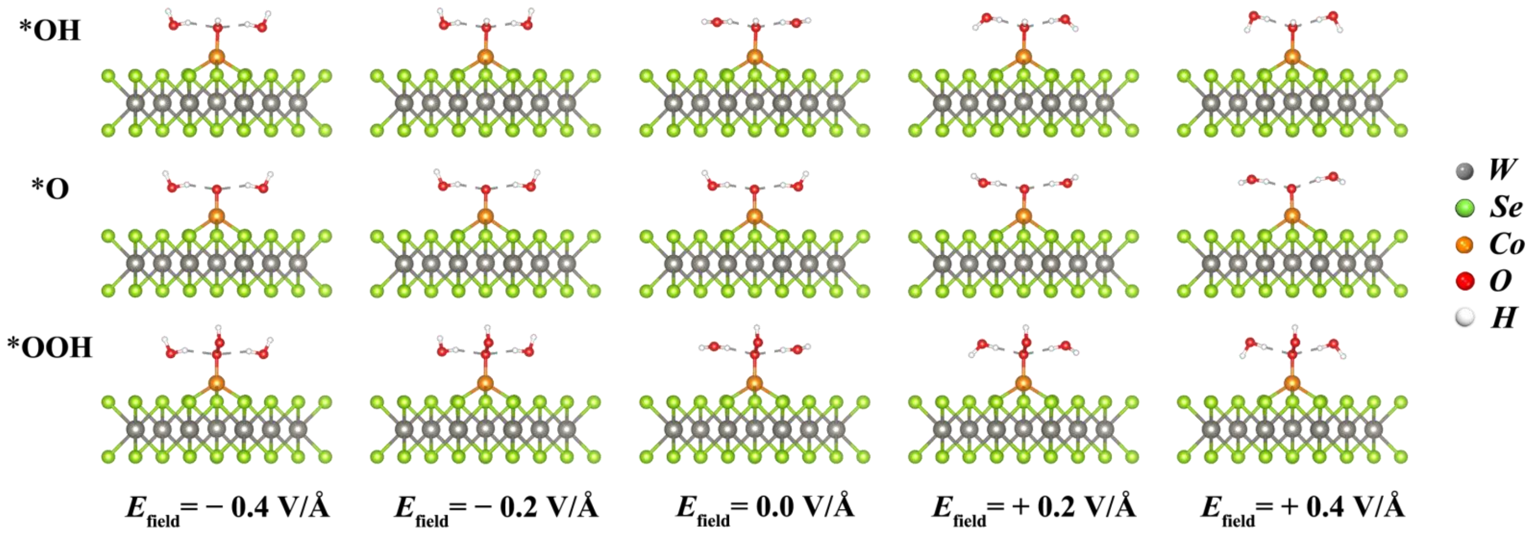

Supplementary Figure 100. The explicit solvation models of (*M-top*)-Co SAs-WSe_2 (*OH, *O, and *OOH adsorbed), with E_{field} applied from −0.4 V/Å to +0.4 V/Å.

Supplementary Table 19. Adsorption free energies of oxygen intermediates (*OH, *O, and *OOH, U=1.23 V) and free energy differences (ΔG_1, ΔG_2 and ΔG_3) in (*M-top*)-Co SAs-WSe_2 (Co atom, gas-phase and explicit solvation models), with E_{field} applied from −0.4 V/Å to +0.4 V/Å.

ΔG (eV)		−0.4 V/Å	−0.2 V/Å	0.0 V/Å	+0.2 V/Å	+0.4 V/Å
ΔG_{OH}	gas-phase	−1.637	−1.687	−1.753	−1.828	−1.917
	solvation	−1.215	−1.443	−1.711	−1.538	−1.559
ΔG_{O}	gas-phase	−1.618	−1.758	−1.908	−2.064	−2.231
	solvation	−1.310	−1.608	−1.902	−1.828	−1.834
ΔG_{OOH}	gas-phase	−0.977	−0.983	−1.010	−1.057	−1.127
	solvation	−0.276	−0.540	−0.824	−0.660	−0.559
$\Delta G_1=\Delta G_{OH}$	gas-phase	−1.637	−1.687	−1.753	−1.828	−1.917
	solvation	−1.215	−1.443	−1.711	−1.538	−1.559
$\Delta G_2=\Delta G_{O}-\Delta G_{OH}$	gas-phase	0.020	−0.071	−0.155	−0.236	−0.314
	solvation	−0.095	−0.166	−0.191	−0.290	−0.275
$\Delta G_3=\Delta G_{OOH}-\Delta G_{O}$	gas-phase	**0.641**	**0.776**	**0.898**	**1.008**	**1.103**
	solvation	**1.034**	**1.068**	**1.078**	**1.168**	**1.275**

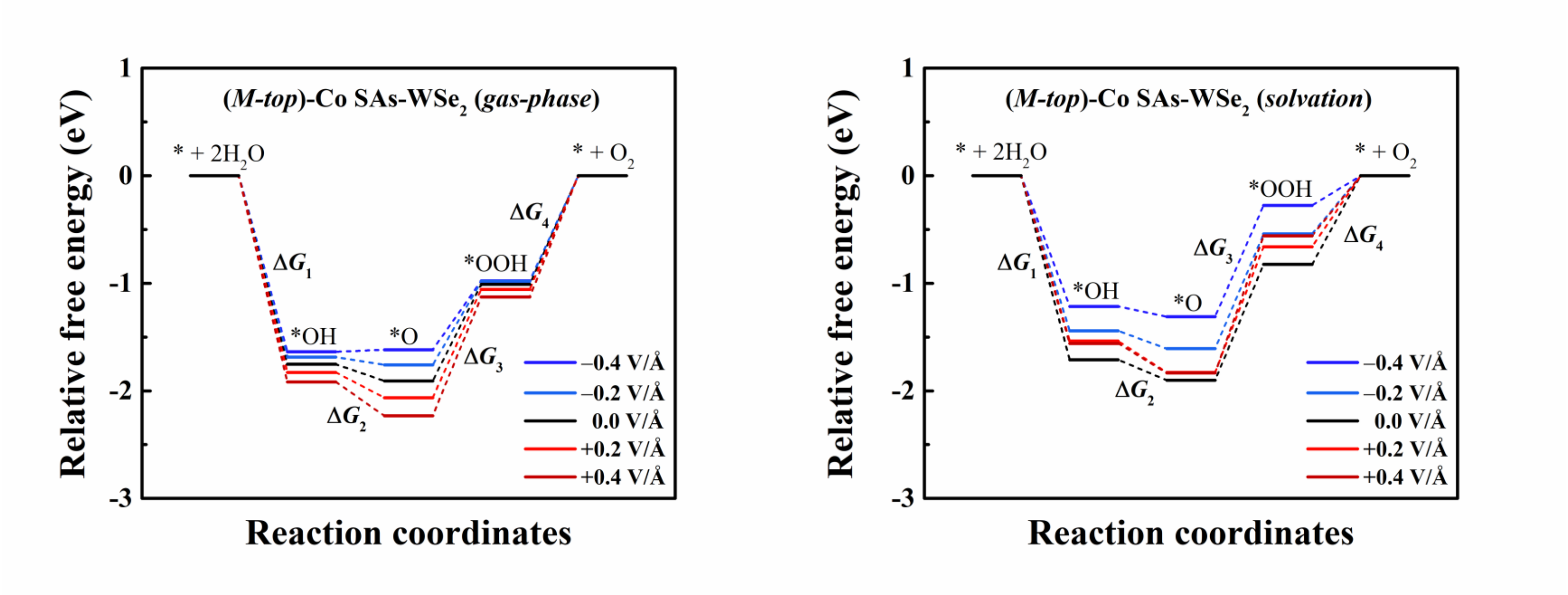

Supplementary Figure 101. Adsorption free energies diagrams of (*M-top*)-Co SAs-WSe_2 (gas-phase and explicit solvation models) for OER under OEEF regulation, where *OH, *O and *OOH is successively absorbed on the exposed Co atom.

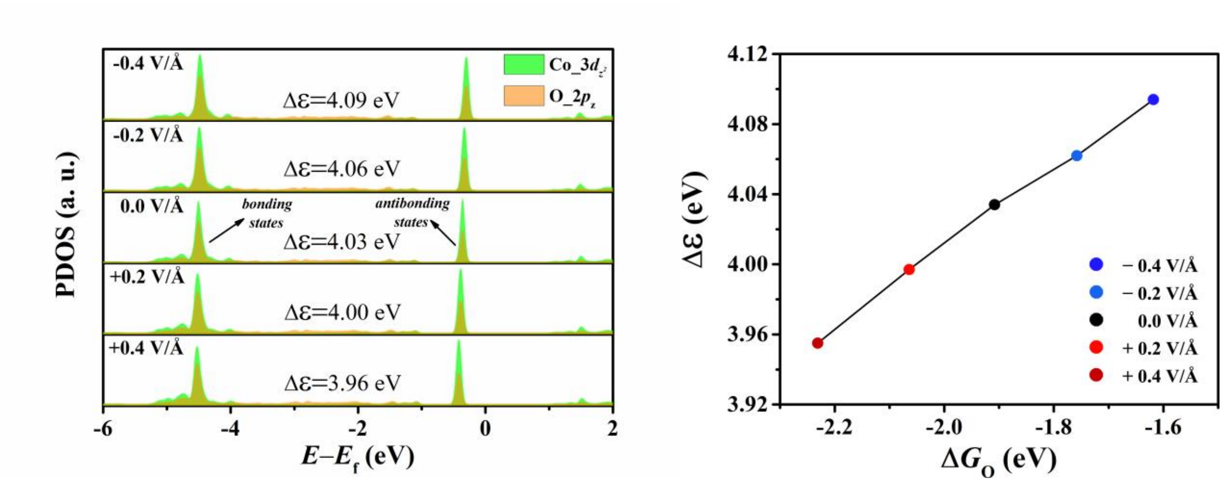

Supplementary Figure 102. PDOS (left) of O $2p_z$ orbital (green) and Co $3d_{z^2}$ orbital (orange) in (*M-top*)-Co SAs-WSe_2 at different OEEFs, showing the energy gap (Δε) between the centers of O $2p_z$-Co $3d_{z^2}$ bonding states and antibonding states and its correlation (right) with oxygen adsorption free energy (ΔG_O).

Supplementary Table 20. Adsorption free energies of oxygen intermediates (*OH, *O, and *OOH, U=1.23 V) and free energy differences (ΔG_1, ΔG_2 and ΔG_3) in (*X-sub*)-Co SAs-WSe_2 (Co atom), with E_{field} applied from −0.4 V/Å to +0.4 V/Å.

ΔG (eV)	−0.4 V/Å	−0.2 V/Å	0.0 V/Å	+0.2 V/Å	+0.4 V/Å
ΔG_{OH}	−1.463	−1.483	−1.505	−1.537	−1.585
ΔG_{O}	−1.135	−1.224	−1.310	−1.395	−1.489
ΔG_{OOH}	−0.751	−0.739	−0.729	−0.727	−0.741
$\Delta G_1=\Delta G_{OH}$	−1.463	−1.483	−1.505	−1.537	−1.585
$\Delta G_2=\Delta G_O-\Delta G_{OH}$	0.328	0.259	0.195	0.142	0.097
$\Delta G_3=\Delta G_{OOH}-\Delta G_O$	**0.384**	**0.486**	**0.581**	**0.668**	**0.748**

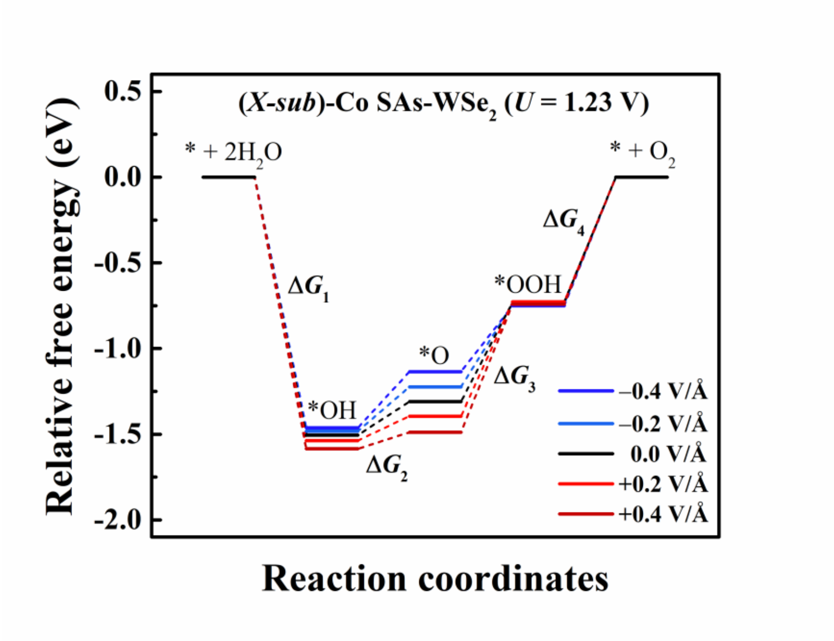

Supplementary Figure 103. Adsorption free energies diagrams of (*X*-*sub*)-Co SAs-WSe_2 for OER under OEEF regulation, where *OH, *O and *OOH is successively absorbed on the exposed Co atom.

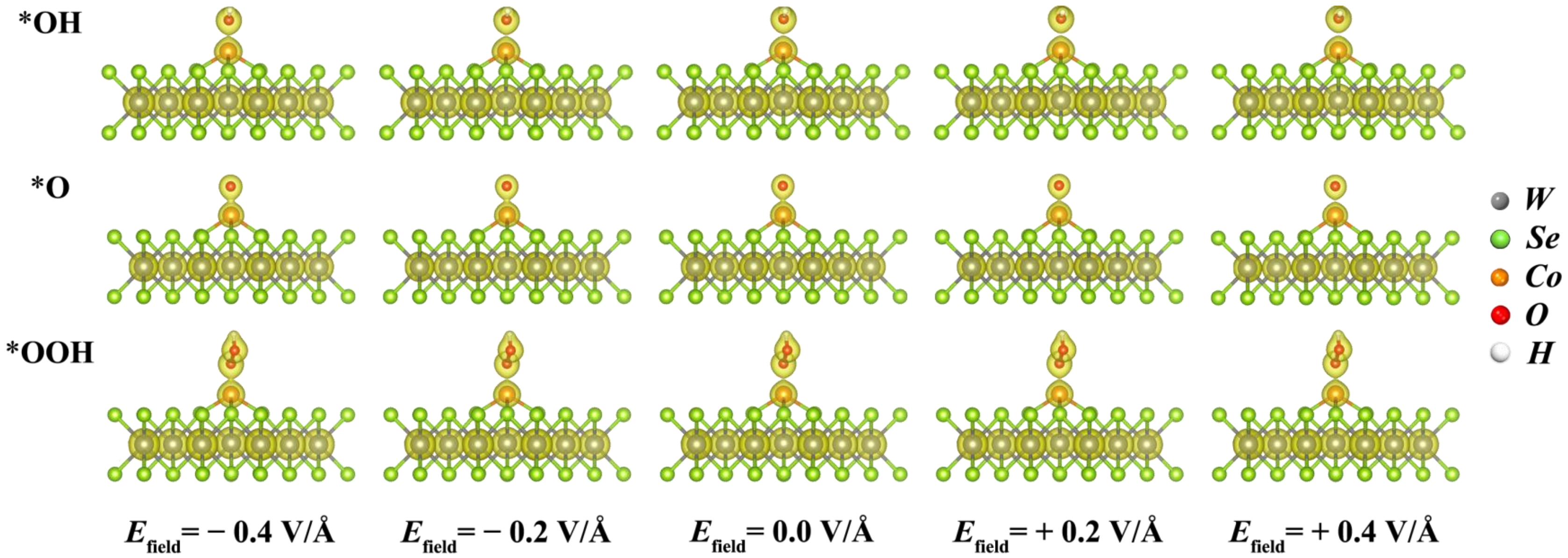

Supplementary Figure 104. Charge densities (yellow) of (*M-top*)-Co SAs-WSe_2 at different OEEFs (E_{field} from −0.4 V/Å to +0.4 V/Å), where *OH, *O and *OOH (from top to bottom) is adsorbed on Co atom, respectively. The interface value is 0.15 $e^-/Bohr^3$.

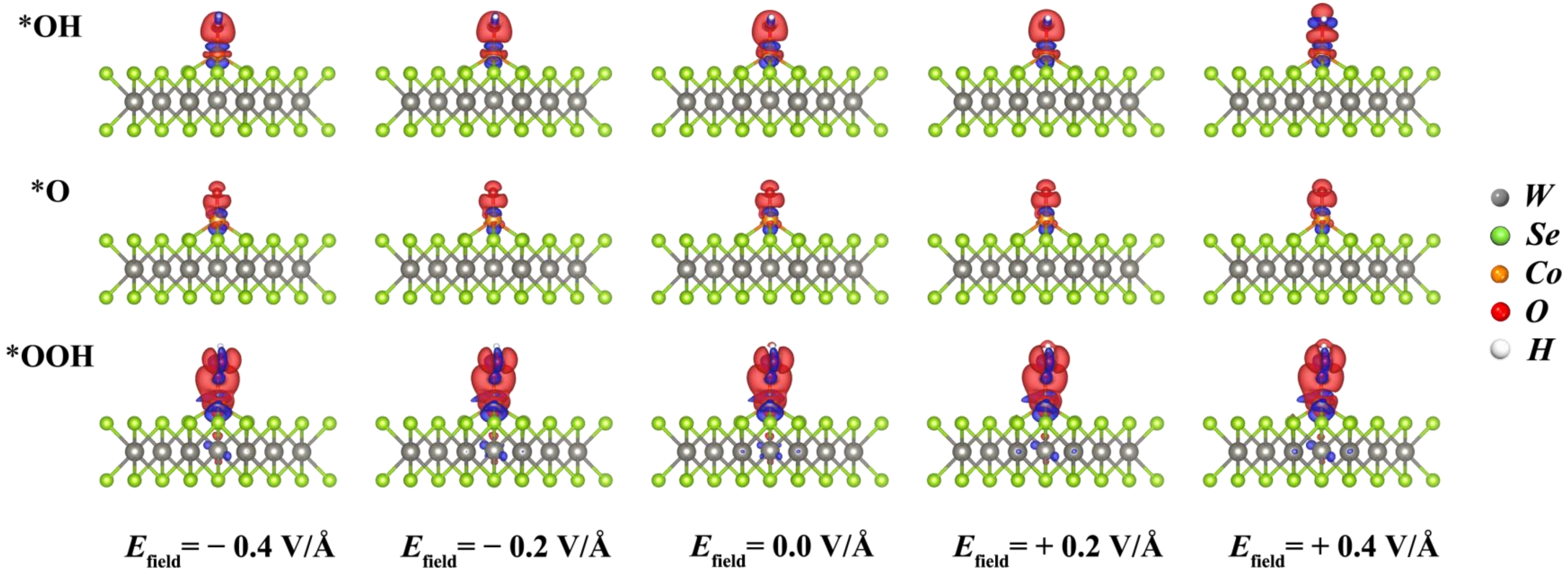

Supplementary Figure 105. Deformation charge densities of (*M-top*)-Co SAs-WSe_2 at different OEEFs (E_{field} from −0.4 V/Å to +0.4 V/Å), where *OH, *O and *OOH (from top to bottom) is adsorbed on Co atom, respectively. The interface value is 0.01 $e^-/Bohr^3$. Red and blue areas denote electron accumulation and depletion, respectively.

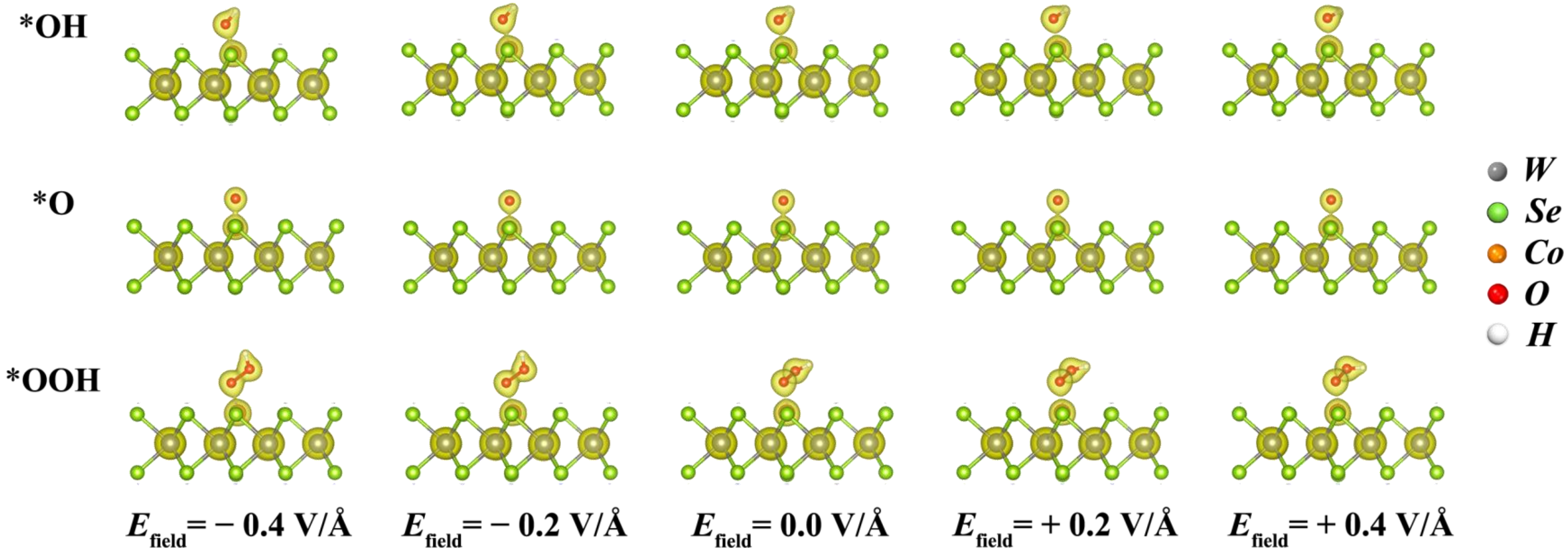

Supplementary Figure 106. Charge densities (yellow) of (*X*-*sub*)-Co SAs-WSe_2 at different OEEFs (E_{field} from −0.4 V/Å to +0.4 V/Å), where *OH, *O and *OOH (from top to bottom) is adsorbed on Co atom, respectively. The interface value is 0.15 $e^-/Bohr^3$.

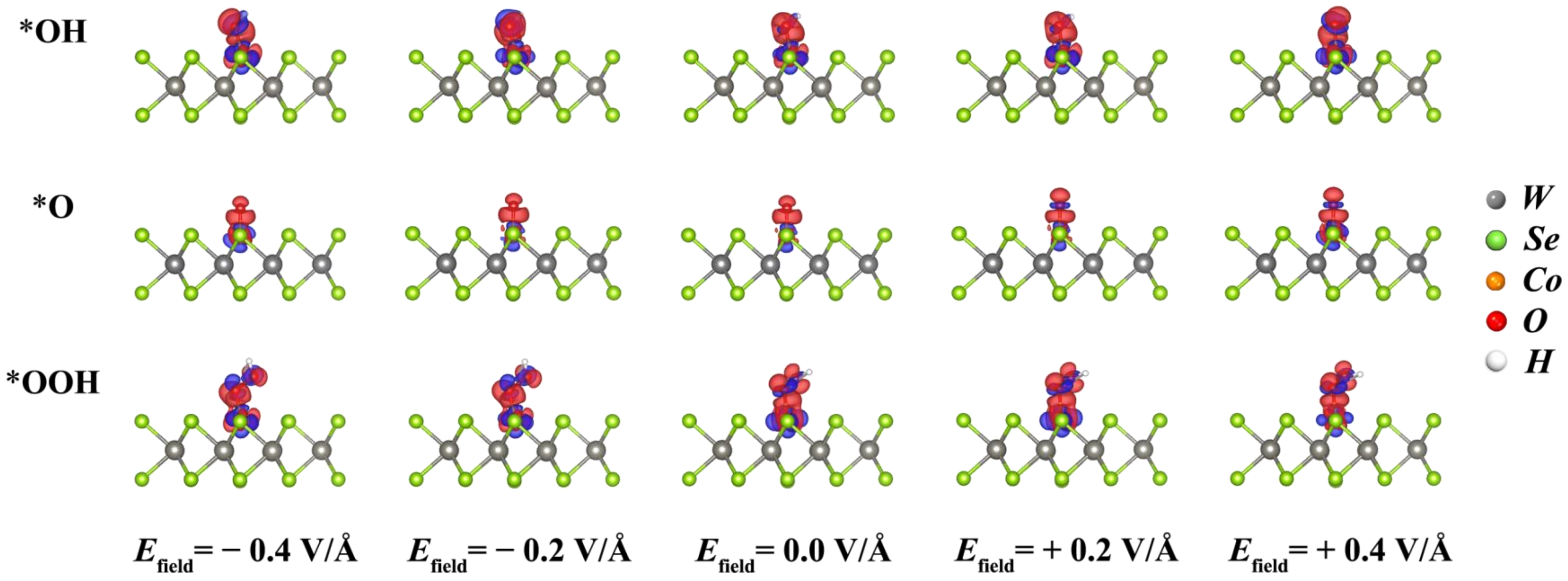

Supplementary Figure 107. Deformation charge densities of (*X*-*sub*)-Co SAs-WSe_2 at different OEEFs (E_{field} from −0.4 V/Å to +0.4 V/Å), where *OH, *O and *OOH (from top to bottom) is adsorbed on Co atom, respectively. The interface value is 0.01 $e^-/Bohr^3$. Red and blue areas denote electron accumulation and depletion, respectively.

Supplementary Table 21. Bader charge of (*M-top*)-Co SAs-WSe_2 (Q_{Co}) and the adsorbed *O, *OH, *OOH (Q_H, Q_{OH}, Q_O and Q_{OOH}), with E_{field} applied from −0.4 V/Å to +0.4 V/Å. Note that the H (1s), O (2s, 2p), Se (4s, 4p), W (5p, 6s, 5d) and Co (4s, 3d) electrons were treated as valance states during all DFT calculations.

(*X-sub*)-Co SAs-WSe_2	Bader charge	−0.4 V/Å	−0.2 V/Å	0 V/Å	0.2 V/Å	0.4 V/Å
with *OH adsorbed	Q_{Co}	8.832	8.841	8.848	8.853	8.860
	Q_O	6.071	6.076	6.082	6.090	6.096
	Q_H	1.398	1.423	1.448	1.473	1.500
	Q_{O+H}	7.469	7.499	7.530	7.563	7.596
with *O adsorbed	Q_{Co}	8.816	8.797	8.775	8.738	8.701
	Q_O	**6.594**	**6.642**	**6.699**	**6.768**	**6.836**
with *OOH adsorbed	Q_{Co}	9.000	9.146	9.386	9.406	9.011
	Q_{O1}	6.376	6.384	6.364	6.377	6.419
	Q_{O2}	6.356	6.323	6.370	6.435	6.407
	Q_H	0.601	0.647	0.629	0.612	0.696
	$Q_{O1+O2+H}$	13.333	13.354	13.363	13.424	13.522

Supplementary Table 22. Bader charge of the adsorbed *O (Q_O) in the gas-phase and explicit solvation models of (*M-top*)-Co SAs-WSe_2, with E_{field} applied from −0.4 V/Å to +0.4 V/Å.

models	Q_O	−0.4 V/Å	0.0 V/Å	+0.4 V/Å
(*M-top*)-Co SAs-WSe_2	gas-phase model	6.594	6.699	6.836
	explicit solvation model	6.470	6.477	6.482

Supplementary Table 23. Bader charge of (*X-sub*)-Co SAs-WSe_2 (Q_{Co}) and the adsorbed *O, *OH, *OOH (Q_H, Q_{OH}, Q_O and Q_{OOH}), with E_{field} applied from −0.4 V/Å to +0.4 V/Å. Note that the H (1s), O (2s, 2p), Se (4s, 4p), W (5p, 6s, 5d) and Co (4s, 3d) electrons were treated as valance states during all DFT calculations.

(*X-sub*)-Co SAs-WSe_2	Bader charge	−0.4 V/Å	−0.2 V/Å	0 V/Å	0.2 V/Å	0.4 V/Å
with *OH adsorbed	Q_{Co}	8.706	8.713	8.751	8.799	8.672
	Q_O	6.859	6.914	6.917	6.908	6.916
	Q_H	0.632	0.632	0.651	0.670	0.699
	Q_{O+H}	7.490	7.547	7.568	7.578	7.615
with *O adsorbed	Q_{Co}	8.531	8.567	8.54	8.543	8.460
	Q_O	**6.597**	**6.622**	**6.665**	**6.715**	**6.692**
with *OOH adsorbed	Q_{Co}	8.601	8.770	8.701	8.819	8.662
	Q_{O1}	6.452	6.410	6.503	6.444	6.487
	Q_{O2}	6.370	6.457	6.485	6.431	6.465
	Q_H	0.623	0.567	0.558	0.652	0.666
	$Q_{O1+O2+H}$	13.446	13.435	13.546	13.527	13.618

Supplementary References

[1] Li, H. *et al.* Activating and optimizing MoS_2 basal planes for hydrogen evolution through the formation of strained sulphur vacancies. *Nat. Mater.* **15**, 364 (2016).

[2] Tsai, C. *et al.* Electrochemical generation of sulfur vacancies in the basal plane of MoS_2 for hydrogen evolution. *Nat. Commun.* **8**, 15113 (2017).

[3] Li, G. *et al.* All The Catalytic Active Sites of MoS_2 for Hydrogen Evolution. *J. Am. Chem. Soc.* **138**, 16632-16638 (2016).

[4] Wang, V. et al. VASPKIT: A user-friendly interface facilitating high-throughput computing and analysis using VASP code. *Comput. Phys. Commun*. **267**, 108033 (2021).

[5] Bjørk, H. & Nørskov, J. Theoretical surface science and catalysis-calculations and concepts. *Adv. Catal*. **45**, 71-129 (2000).

[6] Tang, W., Sanville, E. & Henkelman, G. A grid-based Bader analysis algorithm without lattice bias. *J. Phys. Condens. Matter* **21**, 084204 (2009).

[7] Ling, F. et al. Optimizing edges and defects of supported MoS_2 catalysts for hydrogen evolution via an external electric field. *Phys. Chem. Chem. Phys*. **20**, 26083-26090 (2018).
